# Expression, Prognostic Value, and Functional Mechanism of Polarity-Related Genes in Hepatocellular Carcinoma

**DOI:** 10.3390/ijms232112784

**Published:** 2022-10-24

**Authors:** Dan-Hua Zhu, Yan-Hong Zhang, Xiao-Xi Ou-Yang, Xiao-Hua Meng, Qing-Yi Cao, Xiao-Peng Yu, Juan Lu, Lan-Juan Li, Kun-Kai Su

**Affiliations:** State Key Laboratory for Diagnosis and Treatment of Infectious Diseases, National Clinical Research Center for Infectious Diseases, National Medical Center for Infectious Diseases, Collaborative Innovation Center for Diagnosis and Treatment of Infectious Diseases, The First Affiliated Hospital, Zhejiang University School of Medicine, Hangzhou 310003, China

**Keywords:** hepatocellular carcinoma, cell polarity, elastic net, overall survival, biomarker

## Abstract

Hepatocellular carcinoma (HCC) is a common malignant tumor with high mortality and poor prognoses around the world. Within-cell polarity is crucial to cell development and function maintenance, and some studies have found that it is closely related to cancer initiation, metastasis, and prognosis. The aim of our research was to find polarity-related biomarkers which improve the treatment and prognosis of HCC. For the knowledge-driven analysis, 189 polarity-related genes (PRGs) were retrieved and curated manually from the molecular signatures database and reviews. Meanwhile, in the data-driven part, genomic datasets and clinical records of HCC was obtained from the cancer genome atlas database. The potential candidates were considered in the respect to differential expression, mutation rate, and prognostic value. Sixty-one PRGs that passed the knowledge and data-driven screening were applied for function analysis and mechanism deduction. Elastic net model combing least absolute shrinkage and selection operator and ridge regression analysis refined the input into a 12-PRG risk model, and its pharmaceutical potency was evaluated. These findings demonstrated that the integration of multi-omics of PRGs can help us in untangling the liver cancer pathogenesis as well as illustrate the underlying mechanisms and therapeutic targets.

## 1. Introduction

Liver disease is a global public health problem and causes approximately 2 million deaths worldwide every year [1]. Hepatocellular carcinoma (HCC) accounts for 75–85% of primary liver cancers and is a major global health problem. Most HCC cases are usually detected at an advanced stage and have a poor prognosis. Therefore, understanding the pathogenesis of HCC is crucial for treatment [2].

Cell polarity refers to the orchestrated establishment or maintenance of asymmetries within differentiated cells, and is essential for the development of multicellular organisms, wound healing, and immune response [3,4]. Some cells perform functions through the process of polarization, including epithelial cells, odontoblasts, neurons, immune cells, etc. [5]. Abnormal cell polarity may lead to developmental abnormalities, severe birth defects, or tumorigenesis [6,7,8,9]. Disrupted cell polarity is considered a hallmark of human cancer [10]. Lin et al. found that over-expression of nerve growth factors can accelerate liver cancer progression by inducing defective cell polarity, transition between epithelial and mesenchymal (EMT/MET), and cell cytoskeleton rearrangement, implying there might be an underlying common mechanism of cell polarity between different cell types [11]. Cell polarity is mainly categorized into apico-basal polarity, planar cell polarity, and anterior–posterior polarity. The latter two kinds of polarity were found mainly related to neuron maturation and different development stages [3,12]; meanwhile the apico-basal polarity and related cell–cell adhesion were found to be more involved in many types of cancer development [13].

Hepatocytes are responsible for most of the liver functions, including bile synthesis and secretion, toxin elimination, etc. [14]. Harmonious function of hepatocytes relies on its high-level polarity, which is considered the most complex even in known polarity critical cell types such as immune cells or neurons. Hepatocytes have several apical membranes and a basolateral plasma membrane. Bile canaliculi for bile secretion was formed by the apical membranes of adjacent hepatocytes. Sinusoids, which are comprised of the basolateral plasma membrane shared with neighboring cells, are the crucial location for substance exchange with blood [15]. Hepatocyte polarization is also essential for function such as biliary secretion, and loss of polarity leads to bile secretory failure and even hepatotoxicity [16,17]. Current study mainly focuses on the apico-basal polarity of hepatocytes in HCC development. 

Several polarity-related genes (PRGs) have been investigated in the polarity establishment and maintenance concert. Hepatitis B and C promote hepato-carcinogenesis by down-regulating E-cadherin and activating β-catenin. Hepatitis B virus entry into hepatocytes depends on hepatocyte polarization [18]. Hepatitis C viral protein NS5A disrupts cell polarity in the early stages of viral infection, triggering morphological changes in hepatocytes and increasing the risk of oncogenic transformation [19]. LKB1/STK11 is a kinase-activating kinase of AMPK, and this energy-sensing protein is activated during polarization and interacts with energy production-related polarization. LKB1/STK11 is frequently mutated or undergoes allelic loss in hepatocellular carcinoma [20]. However, due to the complexity of within-cell polarization and the pathogenesis of liver diseases, the molecular role and mechanism of hepatocyte polarization in liver diseases still require further elucidation.

In this study, we focused on the PRGs with accumulated prior knowledge, and explored public multi-omics datasets and clinical records to investigate the association between transcriptomics, (epi-)genetics and prognosis of PRGs, aiming to illustrate their potential role in liver cancer pathogenesis and to explore causative mechanisms for revealed associations.

## 2. Results

### 2.1. Quantitative Screening of Curated Polarity-Related Genes

**Figure 1:**
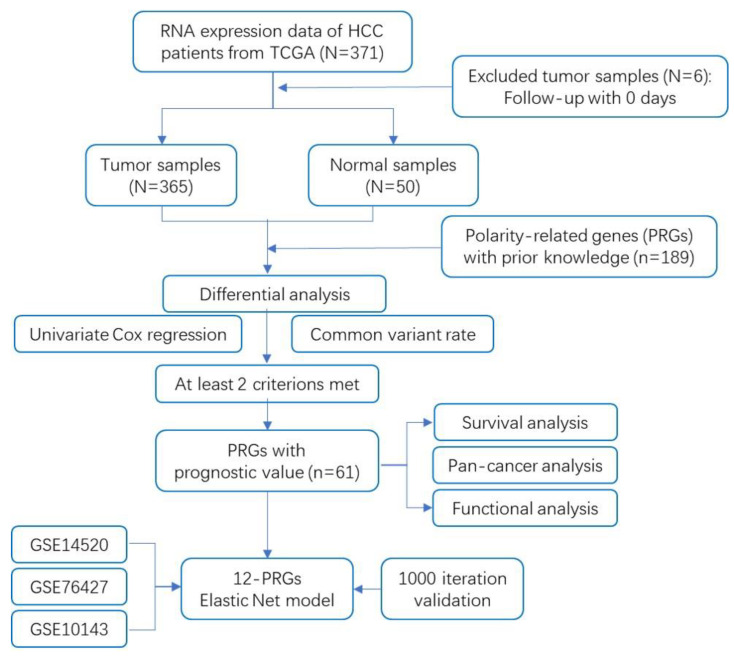
Flowchart of data collection and analysis.

The flow chart of this study is shown in Figure 1. In total, the RNA-sequencing data with detailed prognostic characteristics of 371 liver hepatocellular carcinoma (LIHC) samples were obtained from the cancer genome atlas (TCGA) database. Based on prior accumulated knowledge, 189 PRGs were extracted from the manually curated molecular signatures database (MSigDB) and other rigorous reviews. The retrieval strategy is described in the Methods section. Sixty-one out of 189 genes were screened out based on their different expression, harboring known common variants or prognostic significance, out of which at least two criteria were met. Finally, elastic net model combing least absolute shrinkage and selection operator (LASSO) and ridge regression analysis reduced the 61 variables down to a final 12-PRG signature model. Detailed parameters of each screening step are described in following sections.

### 2.2. Expression Analysis of Prognostic Polarity-Related Genes and Pathways

**Figure 2:**
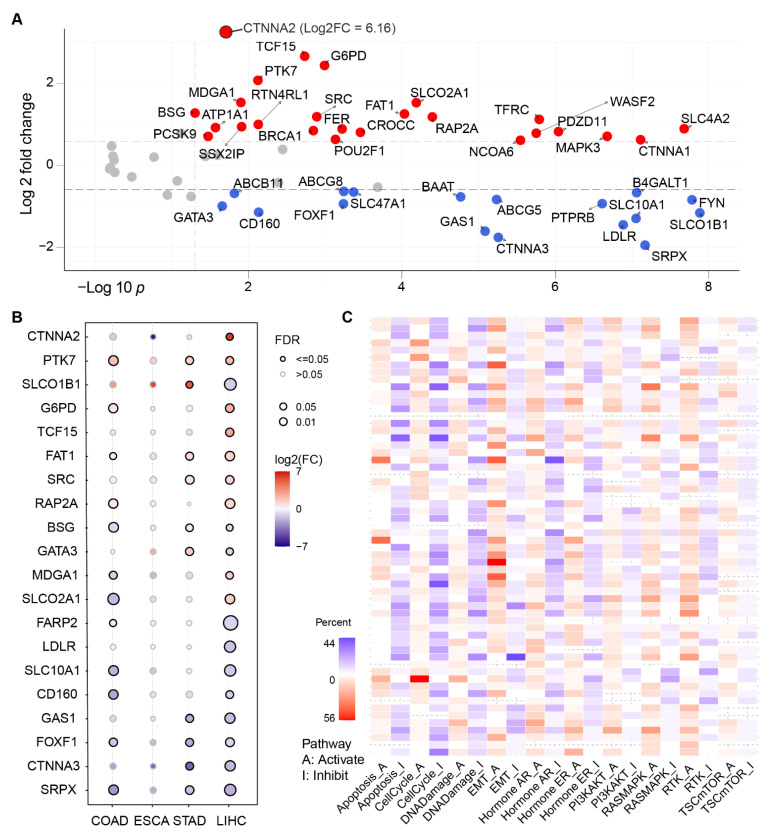
The differential expression analysis of the selected polarity-related genes (PRGs) with clinical value in different tumor tissues and paracancerous tissues of the digestive system from the cancer genome atlas (TGCA) database. (**A**) Volcano plot of 61 PRGs expression change between tumor and matching normal tissues from liver hepatocellular carcinoma (LIHC) with fold change >1.5 and FDR *p* < 0.05. CTNNA2 was separately illustrated. (**B**) The mRNA expression level of selected polarity-related genes across colon adenocarcinoma (COAD), esophageal carcinoma (ESCA), stomach adenocarcinoma (STAD), and LIHC datasets. The larger circle, the more statistically significant. (**C**) EMT and cell cycle pathway are the mostly disrupted by the selected polarity genes across all cancer types deposited in TCGA. The color in each cell indicates that the percentage of altered pathway occurrence in evaluated cancer types. Red for activation, blue for inhabitation.

Expression of curated PRGs was illustrated as a volcano plot in Figure 2A. Twenty-two PRGs exhibited significant differential expression between 50 matched tumor and normal tissues from liver hepatocellular carcinoma (LIHC) cohort. Detailed information of 189 PRGs expression can be found in Supplementary Table S1. Notably, three members from the catenin super family, CTNNA1, CTNNA2, CTNNA3, were found to be dysregulated in tumor tissues. CTNNA2 was the mostly upregulated in all PRGs, yet showed a different direction to other family members.

To understand the expression changes of the selected 61 PRGs, we plotted the mRNA expression of PRGs in four cancer types from the digestive system. In the panorama of gene expression, we observed that LIHC samples hosted more DEGs, compared with the other three functionally related organs in the digestive system. To some extent, the functional relationship was depicted by the similarity of PRG expression patterns. For example, the functionally mostly distal organ esophageal demonstrated the most deviation from the liver in Figure 2B.

Beyond single-gene comparation in digestive system samples, pathway analysis was expanded to the scope of all 33 kinds of cancers. Results exhibited that epithelial-to-mesenchymal transition (EMT), cell cycle, DNA damage, and apoptosis were the mostly dysregulated pathways of PRGs against the background (Figure 2C). In particular, GAS1 and CDH1 from EMT pathway topped across all 33 cancers as the mostly activated and inhibited, respectively, in Supplementary Figure S1 and Table S2.

### 2.3. Gene Set Variation Analysis (GSVA) of Polarity-Related Genes

**Figure 3:**
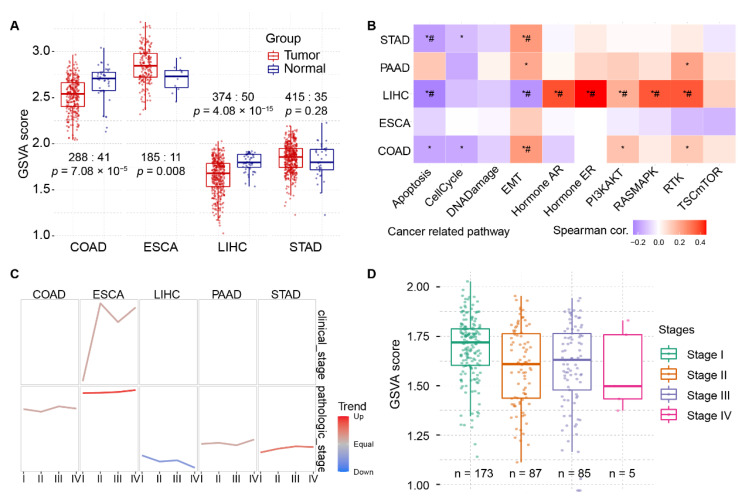
Gene set variation analysis (GSVA) of the selected PRGs with clinical value between different pathologic stages in LIHC and other digestive system cancers. (**A**) Original GSVA score between tumor and paracancerous tissues. (**B**) GSVA-altered pathways in different cancer types from digestive system. (**C**) GSVA trend analysis of 61 polarity-related genes across digestive system cancers. (**D**) Detailed trend analysis in LIHC cohort showed difference between pathological stages. *, *p* < 0.05; #, FDR < 0.05.

Besides single-gene or pathway analysis, gene set variation analysis (GSVA) was performed to characterize pathways or signature summaries from the 61-PRG dataset. The GSVA score was calculated according to GSVA package, and it showed different change patterns across LIHC, COAD, ESCA, and STAD in Figure 3A. LIHC and COAD showed downregulated GSVA score in tumor samples, whereas ESCA showed the opposite pattern. Similar to the single-gene analysis, LIHC showed more pathways affected by PRGs. Pathway of apoptosis, EMT, hormone AR/ER, PI3kAT, RASMAPK and RTK were correlated to GSVA alteration significantly in Figure 3B. Consistent with the tumor/normal changes, when diving into substages of LIHC tumor samples, a downward trend of GSVA score except for stage III was found in Figure 3C. In this step, ESCA showed a surprisingly opposite trend in pathologic stages and clinical stages. The detailed distribution of GSVA score for stages in LIHC were shown in Figure 3D.

### 2.4. Genetic and Epigenetic Alteration of PRGs

**Figure 4:**
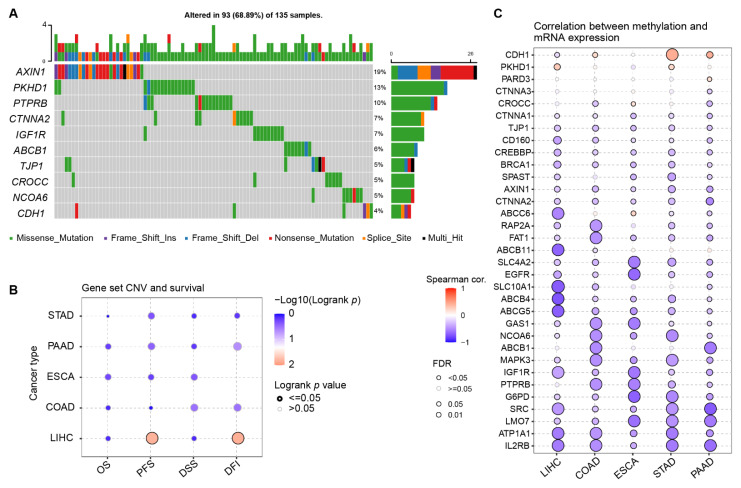
Mutation analysis of the selected PRGs with clinical value in LIHC and other digestive system cancers. (**A**) Single nucleotide variants (SNV) of the top 10 PRGs in LIHC. (**B**) Survival analysis of gene set copy number variants (CNV) across five digestive cancer types. (**C**) Correlation between methylation and mRNA expression of the selected PRGs.

To rank the most protistic or functional PRGs, single nucleotide variant (SNV) and methylation data from TCGA was included in the investigation. The oncoplot in Figure 4A showed the mutation rate in the 135 HCC patients who has at least one mutation in at least one PRG from LIHC cohort. The top 10 PRGs with the most abundant SNV in LIHC were AXIN1, PKHD1, PTPRB, CTNNA2, IGF1R, ABCB1, TJP1, CROCC, NCOA6, and CDH1. In particular, almost 19% of the 135 patients had mutations across the AXIN1 gene. In addition, if AXIN1 was singled out of the 61 PRGs, LIHC demonstrated the highest AXIN1 mutation rate at 26% in all TCGA cancer types in Supplementary Figure S3. In association with survival data, copy number variants (CNV) from LIHC exhibited significant correlation with PFS and DFI in Figure 4B. For all of 61 PRGs, expression was almost negatively correlated to the gene methylation in Figure 4C. All evidence suggests that PRGs are of biological function in HCC development or recurrence.

### 2.5. Protein–Protein Interaction and Prognostic Survial Analysis of PRGs

**Figure 5:**
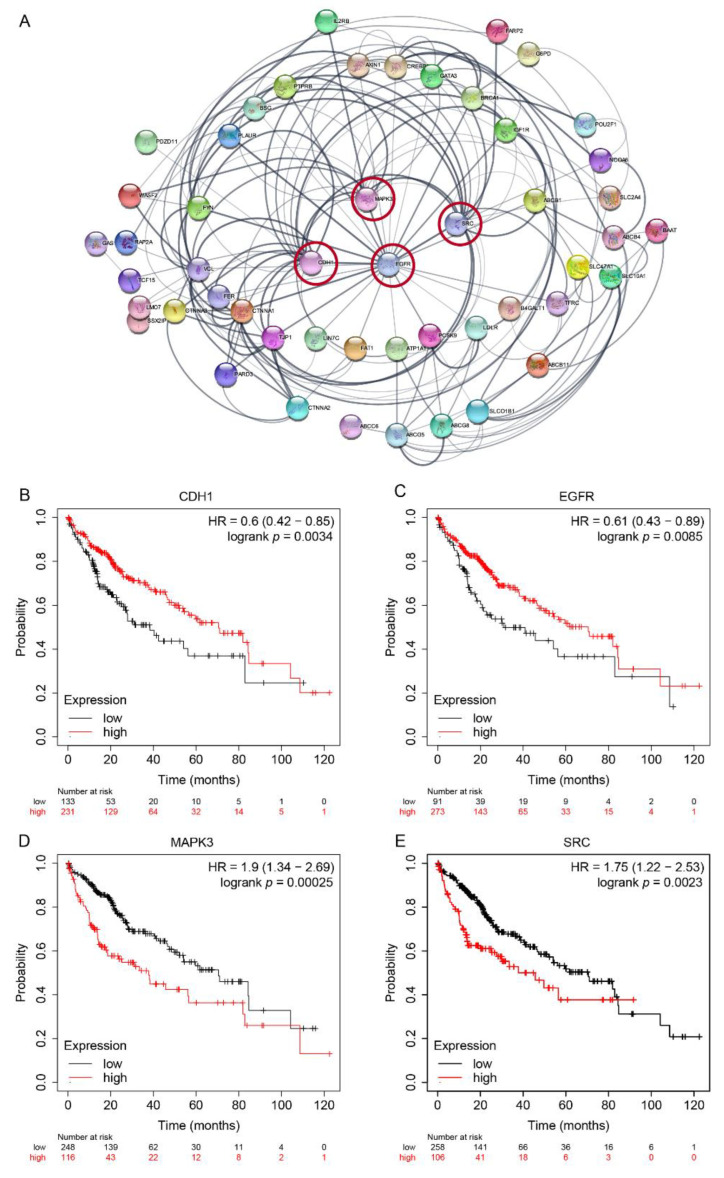
Network and survival curve of polarity-related genes with clinical value. (**A**) Protein–protein interaction (PPI) network of prognostic polarity-related genes. Thickness of connection denotes strongness of interaction evidence. The nodes with highest degree of connectivity were circled. (**B**–**E**) Survival curves of selected top four genes emerged from PPI network.

The curated 61 PRGs were applied to the STRING database for potential functional relationship analysis. Figure 5A showed the known protein–protein interaction (PPI) between each gene pair. Genes were ranked by the degree of connection. We found that EGFR, CDH1, SRC, MAPK3, TJP1, CTNNA1, VCL, FYN, ABCB1, and ABCG5 were the mostly connective genes to each other in the network, and detailed connective values were shown in Supplementary Table S3. Meanwhile, all 61 PRGs were detected for survival analysis. The overall survival curves of selected genes, CDH1, EGFR, MAPK3, and SRC, were shown in Figure 5B–D. Higher CDH1 or EGFR, or lower MAPK3 or SRC lead to better outcomes according to the follow-up record in this LIHC cohort. Survival curves of other PRGs can be found in Supplementary Figure S2.

### 2.6. Twelve-PRG Signature Constructed by Elastic Net Survial Analysis

**Figure 6:**
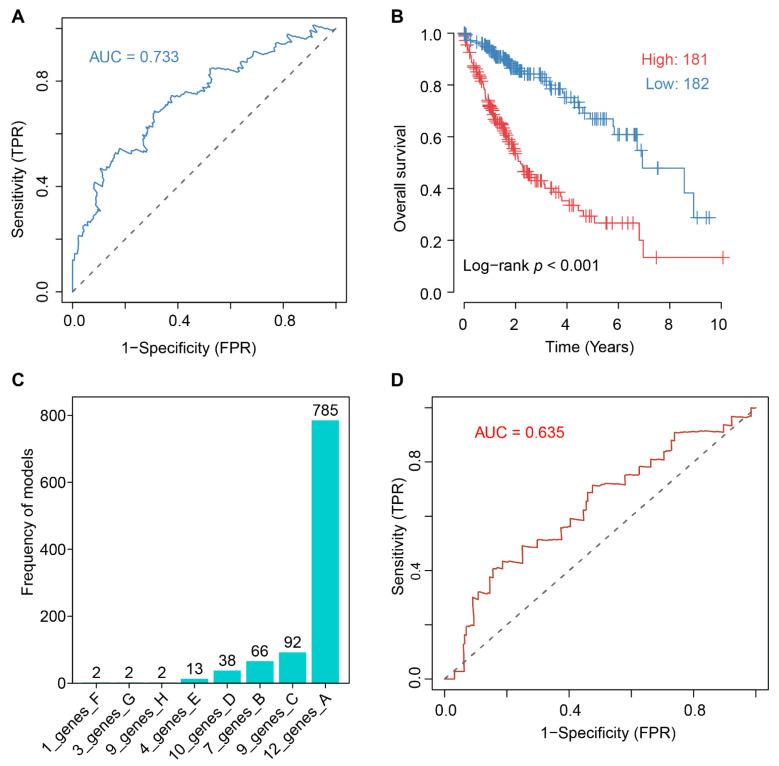
Elastic net-based survival approach for construction of risk score model. (**A**) Receiver operating characteristic (ROC) of the 12-PRG risk score model. (**B**) Performance of risk score model to discriminate included 361 samples. (**C**) Recurrence of models generated by random seeding in 1000-iteration simulation of elastic net modeling. (**D**) Validation of 12-PRG model on GSE14520 cohort.

We performed the elastic net modeling, which combines least absolute shrinkage and selection operator (LASSO)-based and ridges regression of survival analysis to determine PRGs associated with the HCC outcome. The elastic net algorithm reduced the input of 61 selected PRGs to a 12-PRG signature, and the receiver operating curve (ROC) was shown in Figure 6A. The detailed partial likelihood deviance changes when adjusting the penalty parameter lambda in the algorithm were shown in Supplementary Figures S4 and S5. The performance of the models to predict the outcomes of HCC patients was illustrated in Figure 6B.

The risk model could be explicitly displayed as the following formula: Risk score = ABCB1 × 0.0006848492 + AXIN1 × 0.007959001 + CDH1 × 0.0005628617 + CTNNA1 × 0.0002723052 + G6PD × 0.002.875187 + PARD3 × 0.008414534 + RAP2A × 0.004491595 + SLC4A2 × 0.00005756535 + SPAST × 0.0748737 − CD160 × 0.003580824 − LMO7 × 0.01.638609 − PTPRB × 0.03787086, in which the gene symbol denotes its expression.

To evaluate the robustness of the model, a 1000 iteration of modeling were performed to check the recurrence of the 12-PRG models. The 78.5% (785 out of 1000) recurrence in Figure 6C exhibited the outperformance of the current 12-PRG model against others. The area under curve (AUC) of ROC of GSE14520 (*p* = 0.018), GSE76427 (*p* = 0.043), and GSE10143 (*p* = 0.022) in Figure 6D, Supplementary Figure S6 and Table S4.

### 2.7. Drug Sensitivity Prediction and Validation against the 12 PRGs

**Figure 7:**
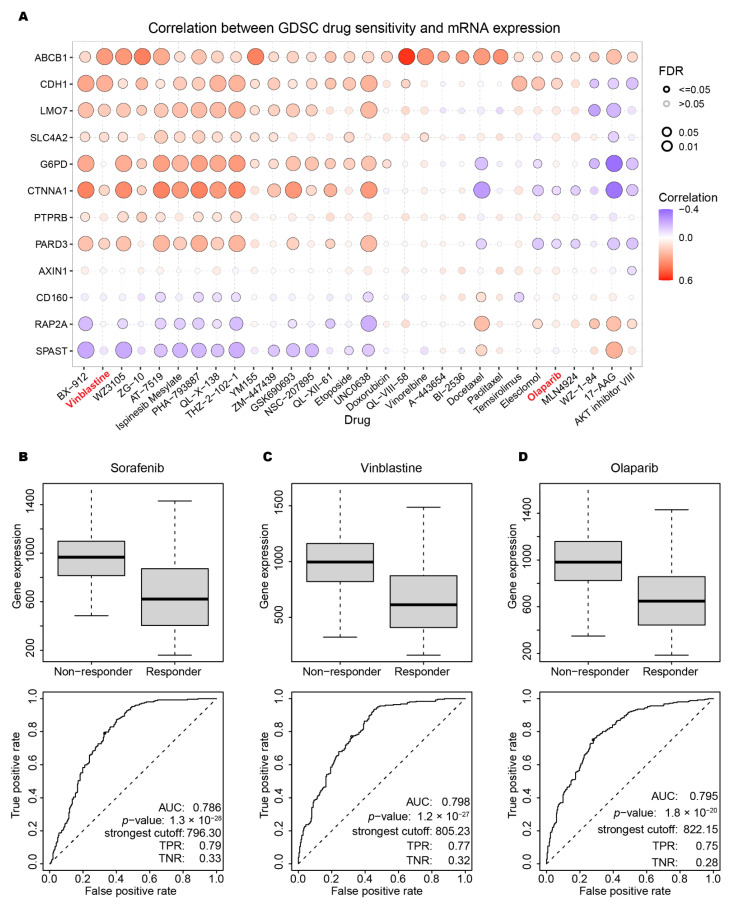
Drugs potency for targeting the 12 PRGs. (**A**) Bubble plot for the top 30 drugs associated with the PRG expression pattern. (**B**–**D**) The approved drug sorafenib, vinblastine, and Olaparib performance on cell lines derived from liver tumor.

After refining 12 out of the original 61 PRGs, we explored the possible approved drugs or promising molecules with neutralizing effect. The Genomics of Drug Sensitivity in Cancer (GDSC) and Cancer Therapeutics Response Portal (CTRP) dataset was utilized to detect the correlation of drug IC50 and targeted 12 PRGs expression. Bubble plot in Figure 7A showed the correlation and FDR *p* value of therapeutic response between 12 PRGs and the top 30 drugs. Sorafenib, vinblastine and olaparib were selected to show the detailed response. As an FDA-approved drug specifically for HCC, sorafenib-treated cell lines can be divided into non-responder and responder groups. The mean expression of the 12 PRGs was significantly down-regulated in responder groups, which indicated that the risk proposed by 12 PRGs was reduced by the administration of sorafenib. By using the AUC of 0.786 of sorafenib in Figure 7B as a benchmark, we examined all the top 30 drugs showing correlation. Vinblastine and olaparib, which were not specifically designed or approved for HCC treatment yet, emerged as comparable candidates to sorafenib from the point of our 12-PRGs signature. The results in Figure 7C,D implied that these two compounds might be promising candidates in application in HCC administration in the future. Results for all the approved HCC drugs and top 30 GDSC drugs was found in Supplementary Table S5. Most of CTRP drugs did not show exciting results in individual investigation, as in in Supplementary Figure S7.

## 3. Discussion

The prognosis of HCC patients is poor, and usually left with very limited treatment options [21]. Hepatocyte polarity is of more and more concern in the research of HCC. In this study, we used the multi-omics data from TCGA to evaluate the knowledge-deduced PRGs in a data-driven manner. 

It is of no surprise that EMT, apoptosis and other pathways were found correlated in HCC in both single and GSVA analysis in Figure 2C and Figure 3B. EMT and its reverse counterpart MET grant cells, especially tumor cells, to invade and metastasize. EMT and polarity-related pathways share many genes because they are both related to the cell skeleton or structure maintenance [22,23,24]. Depolarization of cells could be a sign of disfunction which triggers apoptosis mechanism [25].

However, the function of PRGs in certain pathways could be controversial, and this leads to the phenomena that a pathway is both activated or inhibited by PRG dysregulation. In general, all curated PRGs participate in polarity establishment, maintenance, or other related molecular procedures. However, the expression trend of certain PRGs could be ambiguous or controversial. Take FAT atypical cadherin 1 (FAT1) as an example. Its expression differs between cancers or even datasets of certain cancers [26,27,28,29,30]. CTNNA is a family of genes encoding proteins that play an important role in cell–cell adhesion by interacting with cadherins-1 (CDH1) and the actin filaments. CDH1 and actin are both important factors in cell polarity, but usually located in different areas inside the cell. Biallelic truncating mutations result in the loss of CTNNA2 in neurons, and lead to defects in neurite stability and migration [31]. Therefore, the controversial results like CTNNA family could be arbitrary if only expression data was considered to screen useful genes of interest in this kind of analysis. In addition to the expression analysis, we introduce two other factors of mutation rate and survival analysis for potential prognostic PRGs in the following steps. 

Although there are totally 33 cancer types deposited in the TCGA data hub, only 22 types have 150 or more samples collected. Figure 2B showed that ESCA expression patterns of PRGs differed from LIHC, the most in Figure 2B and Figure 3A–C, which gave us a hint that the pattern could be parallel to the distance physically or functionally between organs. This is because normal samples from the pancreas, the most related organ of liver, were insufficient for this analysis. It still needs to be investigated in more comprehensive pan-cancer analysis to a solid conclusion.

AXIN1 is among the most frequently mutated genes in many types of cancer. Its highest mutation rate of 7.12% is found in LIHC in Supplementary Figure S1. Therefore, AXIN1 has great potential to serve as a target or prognostic biomarker in HCC treatment. This gene encodes a cytoplasmic protein containing a regulation domain of G-protein signaling a disheveled and axin domain. Its mature product degrades catenin beta-1 (CTNNB1) and plays the role of a negative regulator of the Wnt signaling pathway [32]. In a conditional knockout mice model, loss-of-function-mutated AXIN1 is found to trigger downstream of YAP/TAZ-centered hippo signaling pathway in hepatocarcinogenesis [33].

CDH1 is a crucial gene involved in cell polarity, EMT, and other related pathways in HCC. From the TCGA–LIHC data, we found that CDH1 is a protective factor for HCC patients, which is consistent with previous studies [34,35]. Again, although CDH1 and other genes were picked by degree of connection in Figure 5A, this could be biased because the connection relies on the amount of research accumulated and curated for that certain gene [36]. Therefore, dependency on only one dimension of genomics data could lead to biased conclusion. To avoid this bias, we conducted a screening strategy to cover three dimensions of genomic data in addition to the manual curation of prior knowledge as described in the Methods section.

The curse of dimension is still a big obstacle in biomedical research due to the limited number of samples compared with the number of features [37]. Sadly, with the improvement of the high-throughput technology, this problem will worsen in the future. One comprising approach is to use the LASSO-like or ridge regression algorithm to reduce the features to a reasonable scale in a clinically acceptable manner. Here, after a proper retrieval strategy, back-to-back curation, and multi-omics analysis, 61 PRGs was shrunken to a final 12-PRG risk score model. The use of the elastic net algorithm which combines LASSO and the ridge regression model allowed us to integrate PRGs from multiple evaluations into one model, which performed better than that of single PRG or single strategy alone. Elastic net evaluates the trade-off between LASSO and ridge by adjusting the alpha parameter, and simply, LASSO and ridge are special cases when the alpha is set to 1 or 0, respectively. The ability of minimizing the bias, elastic net model was used in accumulating studies for small size cohorts [38].

From the GDSC drug screening data, we primarily got access to pharmaceutical potency of the 12 PRGs. Two approved cancer drugs, vinblastine and olaparib, demonstrated encouraging results in cell lines derived from liver tumor, as shown in Figure 7C,D. This could be indirect evidence for possible expansion of those existing drug markets. However, hepatocytes behave quite differently in terms of polarity characteristics in the traditional in vitro 2D culture condition [39,40]. An in vitro collagen sandwich system sheds light on the dilemma, in which primary hepatocytes are isolated and cultured inside two layers of collagen, showing the exciting ability to re-establish cell polarity and maintain hepatic functions [41]. Emerging 3D or organoid culturing system could provide more accurate drug simulation data as in vivo hepatocyte in the future.

## 4. Materials and Methods

### 4.1. Patients and Datasets

TCGA is a free comprehensive data portal for cancer genome data, including mRNA expression, mutation profile, and methylation data (https://cancergenome.nih.gov (accessed on 30 August 2022)). All 33 cancer types from the TCGA database were retrieved in pan-cancer analysis to obtain the landscape view of PRGs. Patients with LIHC contains data from 371 HCC tissues and 50 adjacent nontumorous liver tissues. The available genomic data and matching follow-up data (pathologic stage, histologic grade, overall survival time, gender, and age) from LIHC were utilized for liver-specific PRG screening. For inter-cancer comparation step, to make the results more readable in certain analysis, only cancer types of the digestive system, like colon adenocarcinoma (COAD), esophageal carcinoma (ESCA), pancreatic adenocarcinoma (PAAD), stomach adenocarcinoma (STAD), and LIHC were exhibited. Notably, in some cases, PAAD and other cancers might not generate results because of the insufficient matching of normal samples available.

### 4.2. Manual Screening of Polarity-Related Genes with Prior Knowledge 

We retrieved the PRGs from the MSigDB database (https://www.gsea-msigdb.org/gsea/msigdb/index.jsp (accessed on 30 August 2022)) [42]. In our retrieval strategy, keyword “Polarity OR Polarization” was used and filtered by organism of “Homo sapiens” to extract all possible manually curated gene sets. Based on a back-to-back evaluation, the pathway specifically related to immune or neuro cell activation was excluded first, and the pathway related to planar, anterior, or posterior polarity was also excluded. After the manual screening, the PRGs from “GOBP_ESTABLISHMENT_OF_EPITHELIAL_CELL_APICAL_BASAL_POLARITY”, “GOBP_MAINTENANCE_OF_APICAL_BASAL_CELL_POLARITY”, “HALLMARK_APICAL_SURFACE”,“KEGG_ADHERENS_JUNCTION”, “HALLMARK_APICAL_SURFACE”, and others curated from rigorous reviews [15,43,44] were included for following study, and more details were included in Supplementary Table S1.

### 4.3. Differential Expressed Gene Analysis of Polarity-Related Genes

RNA-seq expression of 50 normal liver samples and the matching tumor samples from TCGA-LIHC patients was used for differentially expressed gene analysis. DEGs were defined by R package edgeR with the criterion of fold change >1.5 and FDR *p* < 0.05. Then, previously selected 189 curated PRGs were intersected with DEGs for expression analysis. The volcano plot was illustrated by EnhancedVolcano package. 

To evaluate the PRGs in a pathway level, the cancer proteome atlas (TCPA) database covering 10 cancer-related pathways (TSC/mTOR, RTK, RAS/MAPK, PI3K/AKT, Hormone ER, hormone AR, EMT, DNA damage response, cell cycle, apoptosis pathways) by proteomic analysis by using 181 high-quality antibodies was retrieved [45]. The pathway score is to sum all the relative protein level of all positive minus negative regulatory components in a particular pathway. TCPA samples were divided into two groups based on expression of gene of interest, and the pathway potentially affected is selected by *t* test applied to pathway score difference of two groups [46].

GSVA is a gene set enrichment analysis which estimates the variation of gene set activity (represented as GSVA score) over a specific cancer’s sample population in an unsupervised manner. [47] The GSVA score represents the integrated level of the expression of gene set, and was performed by applying GSVA package on the downloaded LIHC expression dataset.

### 4.4. Genetical and Epigenetical Alteration of Polarity-Related Genes

Common mutations which emerged in at least 1% of the cohort were considered as a criterion for the PRGs selection. The Oncoplot package was used for the exhibition of SNV. The methylation level was linked to the mRNA expression for possible epigenetic explanation.

### 4.5. Pan-Cancer Analysis of Gene Set Cancer Analysis

GSCA (http://bioinfo.life.hust.edu.cn/GSCA/ (accessed on 30 August 2022)) is an interactive web application based on the TCGA database for pan-cancer investigation [48]. Between LIHC and other cancer types, gene differential expression, overall survival, single nucleotide variation, CNV, methylation and pathway activity were compared by the facility of the GSCA website.

### 4.6. Construction of the PRGs Protein–Protein Interaction Network

The STRING database version 11 (http://string-db.org/) aims to provide a global view of all the available interaction data between biomedical terms, especially genes and related terms [36]. To obtain known and predicted associations, PRGs were submitted to STRINGDB for possible upstream, downstream, or interaction relationship. The results are displayed in the form of radial layout by Cytoscape 7.2. Plugin cytoHubba was used to rank the degree of connection between nodes.

### 4.7. Kaplan–Meier Plotter for Survival Analysis 

The Kaplan–Meier plotter (https://kmplot.com/) curated gene expression data and survival information from TCGA, GEO, and other recognized resources for prognostic evaluation [49]. PRGs candidates were applied on KMPlot to investigate their potential prognostic.

### 4.8. Construction of a Prognostic Risk Model by Elastic Net Algorithm

A two-out-of-three strategy was applied to 189 curated PRGs to get a more solid candidate set which met at least two criteria: (1) differentially expressed in 50 tumor-normal matching samples analysis, (2) harboring common mutations happening more than 1% of 371 subjects and (3) exhibiting potential prognostic value in single COX analysis. Elastic net model, analysis was then employed to establish a prognostic model by using the expression profile of the 61 screened PRGs. Elastic net combines the LASSO and ridge regression approaches by evaluating the trade-off between the two approaches. A 12-gene signature was identified based on the optimal value of AUC and λ in elastic net model. 

### 4.9. Model Validation of the 12-PRGs Model

Two approaches were performed to validate the robustness of the 12-PRGs model. First, to avoid the randomness introduced by the seeding steps in the elastic net, the model construction was iterated 1000 times to check the recurrence of each possible models, and the mostly recurrent one is the most promising [50]. Secondly, HCC studies with clinical follow-up information and a reasonable cohort size were used as independent datasets. GSE14520 [51,52], GSE10143 [53] and GSE76427 [54] were retrieved and their ROC [55] was calculated to evaluate the model generated from TCGA-LIHC cohort.

### 4.10. Screening of Drugs Potentially Targeting the 12 PRGs Signature

GDSC and CTRP projects access to more than 1000 cell lines to evaluate interactions with more than 500 drugs in a high-throughput manner [56]. The GDSC and CTRP data was used to primarily evaluate current approved HCC drugs and potential innovative compounds according to the lower vs. upper tercile of IC50 in vitro [57].

## 5. Conclusions

In this study, we conducted an in-depth exploration of polarity-related genes in HCC, and a 12-gene risk score model was built to stratify HCC patients. This will facilitate the future personalized treatment plan to prolong individual survival time. The 12 PRGs also provided the community a bundle of refined targets for a better understanding of hepatocyte polarity in HCC research.

## Data Availability

All the datasets are freely available. TCGA data was downloaded from https://portal.gdc.cancer.gov/. GSE data was downloaded from https://www.ncbi.nlm.nih.gov/geo as in Supplementary Table S4.

## Supplementary Materials

The following supporting information can be downloaded at: https://www.mdpi.com/article/10.3390/ijms232112784/s1.
