# Supplementary material for: Expression, Prognostic Value, and Functional Mechanism of Polarity-Related Genes in Hepatocellular Carcinoma
Source: Int J Mol Sci. 2022 Oct 24;23(21):12784. doi: 10.3390/ijms232112784 (PMC9655479; doi:10.3390/ijms232112784)
Supplement: Supplementary file 1 [file ijms-23-12784-s001.zip › Supplementary Figure_S2_Survival curves of 61 PRGs.pdf]

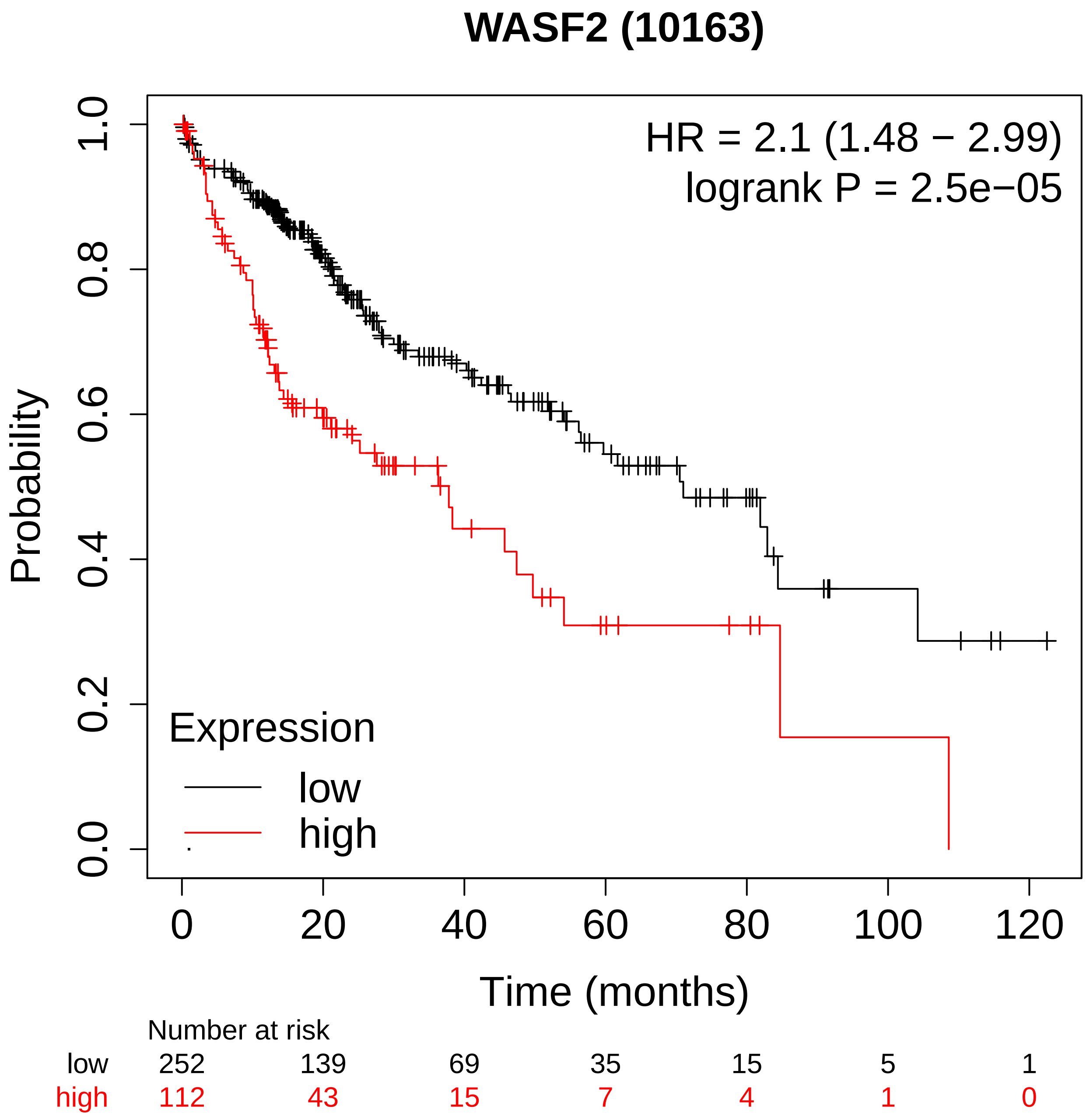
WASF2 (10163)
HR = 2.1 (1.48 − 2.99)
logrank P = 2.5e−05
Probability
1.0
0.8
0.6
0.4
0.2
0.0
Expression
low
high
0
20
40
60
80
100
120
Time (months)
Number at risk
low 252 139 69 35 15 5 1
high 112 43 15 7 4 1 0

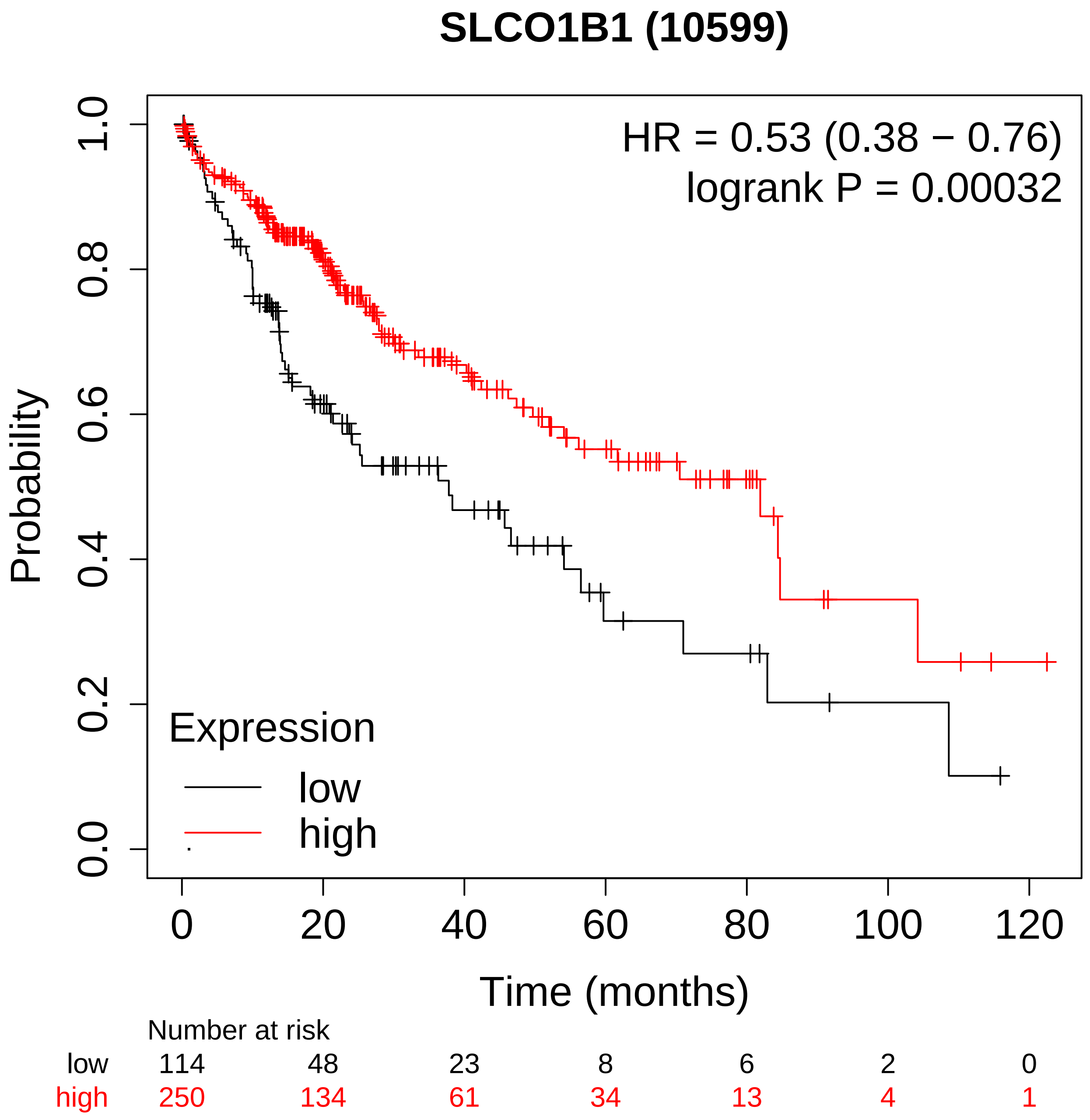

SLCO1B1 (10599)
HR = 0.53 (0.38 − 0.76)
logrank P = 0.00032
Probability
1.0
0.8
0.6
0.4
0.2
0.0
Expression
low
high
0
20
40
60
80
100
120
Time (months)
Number at risk
low 114 48 23 8 6 2 0
high 250 134 61 34 13 4 1

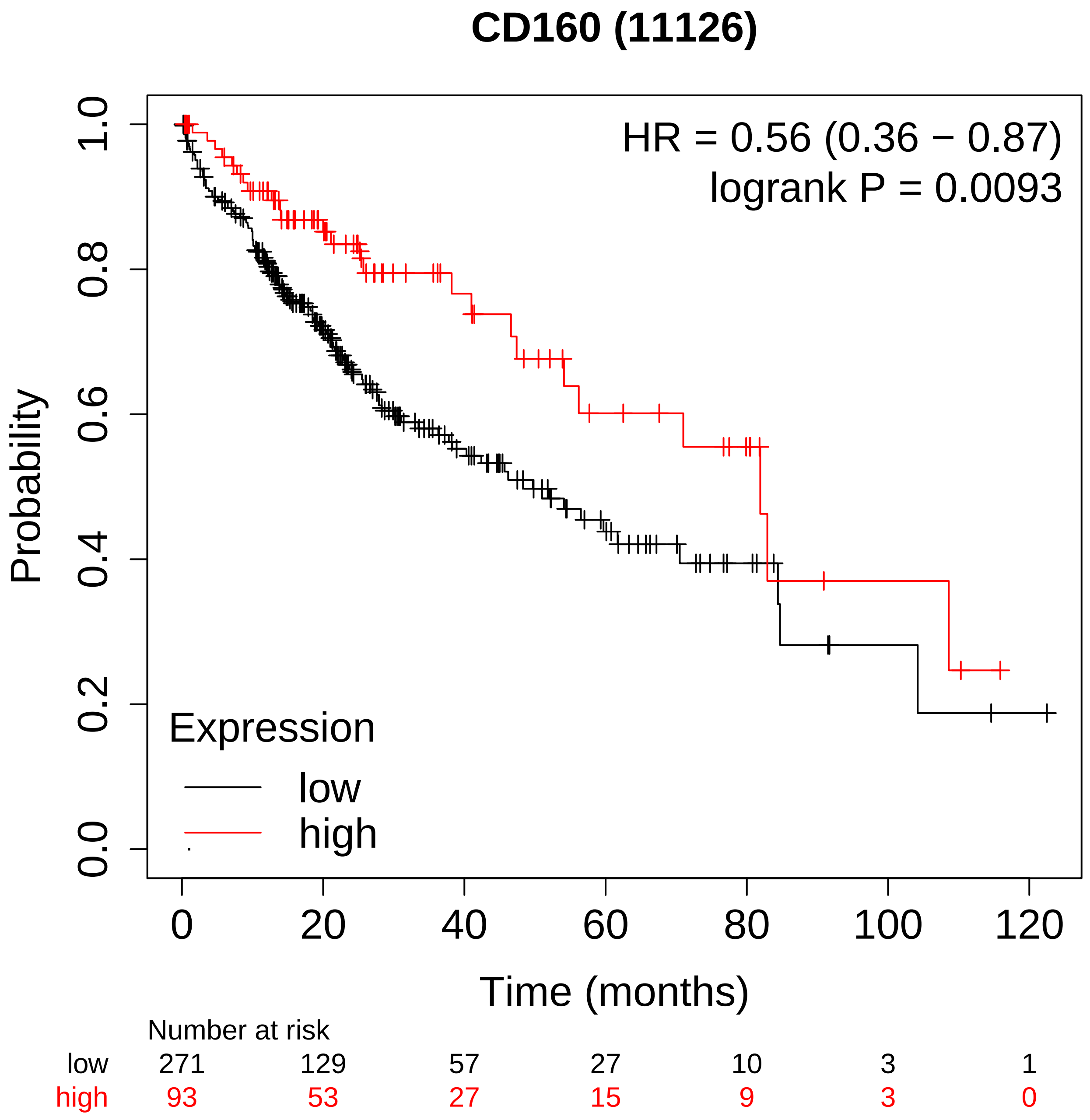
CD160 (11126)
HR = 0.56 (0.36 − 0.87)
logrank P = 0.0093
Probability
1.0
0.8
0.6
0.4
0.2
0.0
Expression
low
high
0
20
40
60
80
100
120
Time (months)
Number at risk
low 271 129 57 27 10 3 1
high 93 53 27 15 9 3 0

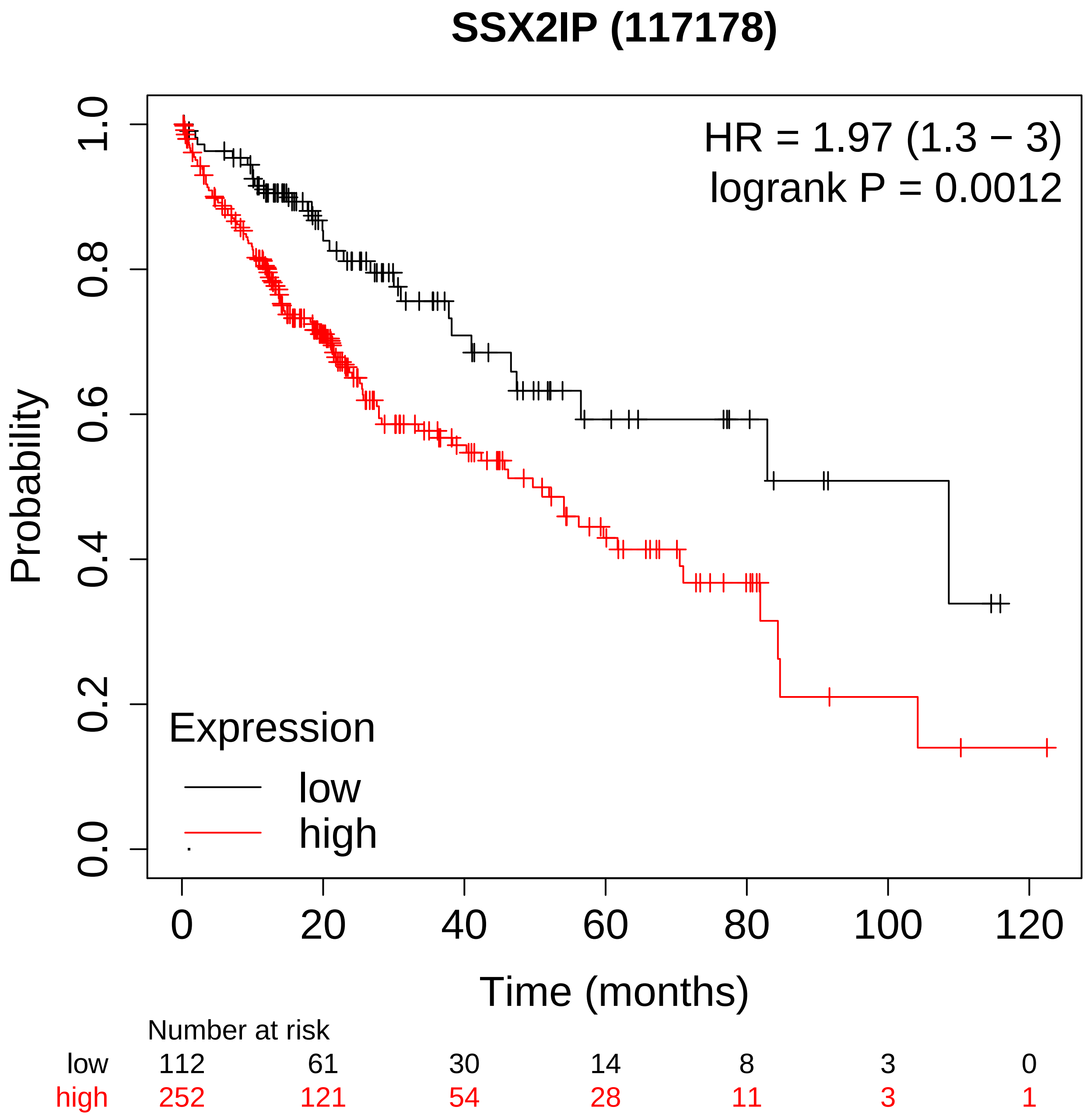

SSX2IP (117178)
HR = 1.97 (1.3 − 3)
logrank P = 0.0012
Probability
0.0
0.2
0.4
0.6
0.8
1.0
Expression
low
high
0
20
40
60
80
100
120
Time (months)
Number at risk
low 112 61 30 14 8 3 0
high 252 121 54 28 11 3 1

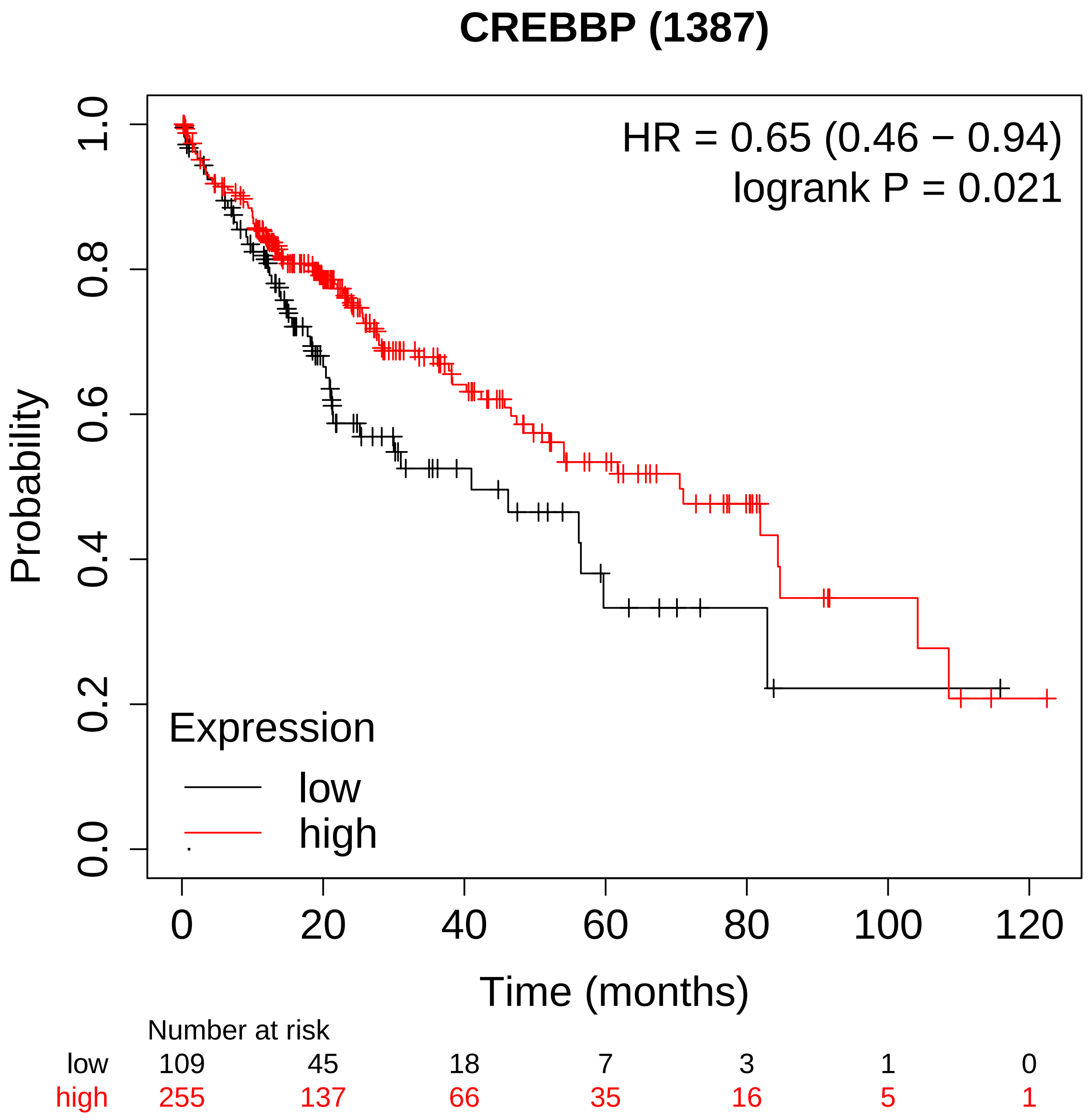

CREBBP (1387)
HR = 0.65 (0.46 − 0.94)
logrank P = 0.021
Probability
0.0
0.2
0.4
0.6
0.8
1.0
Expression
low
high
0
20
40
60
80
100
120
Time (months)
Number at risk
low 109 45 18 7 3 1 0
high 255 137 66 35 16 5 1

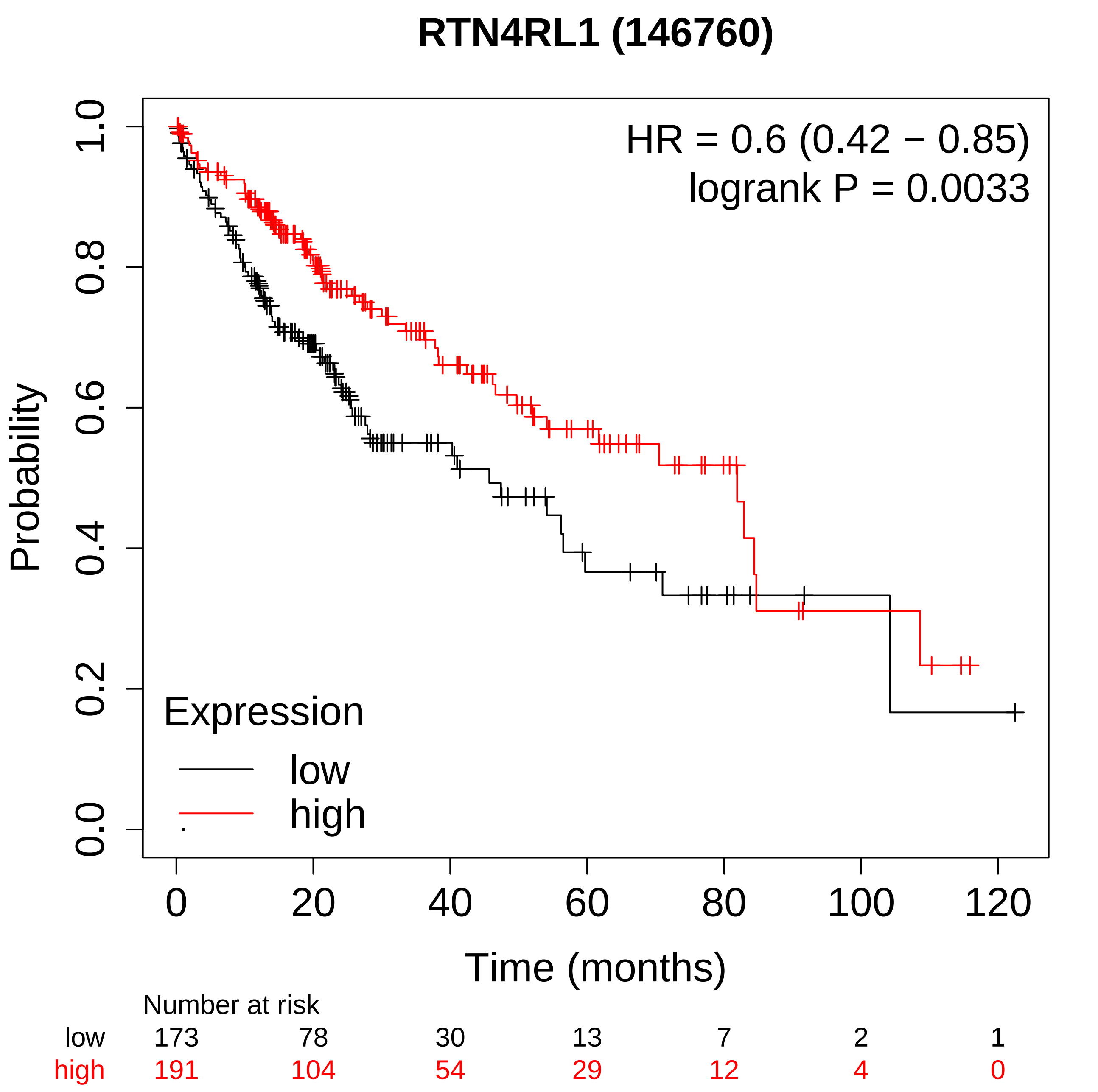

RTN4RL1 (146760)
HR = 0.6 (0.42 − 0.85)
logrank P = 0.0033
Probability
0.0
0.2
0.4
0.6
0.8
1.0
Expression
low
high
0
20
40
60
80
100
120
Time (months)
Number at risk
low 173 78 30 13 7 2 1
high 191 104 54 29 12 4 0

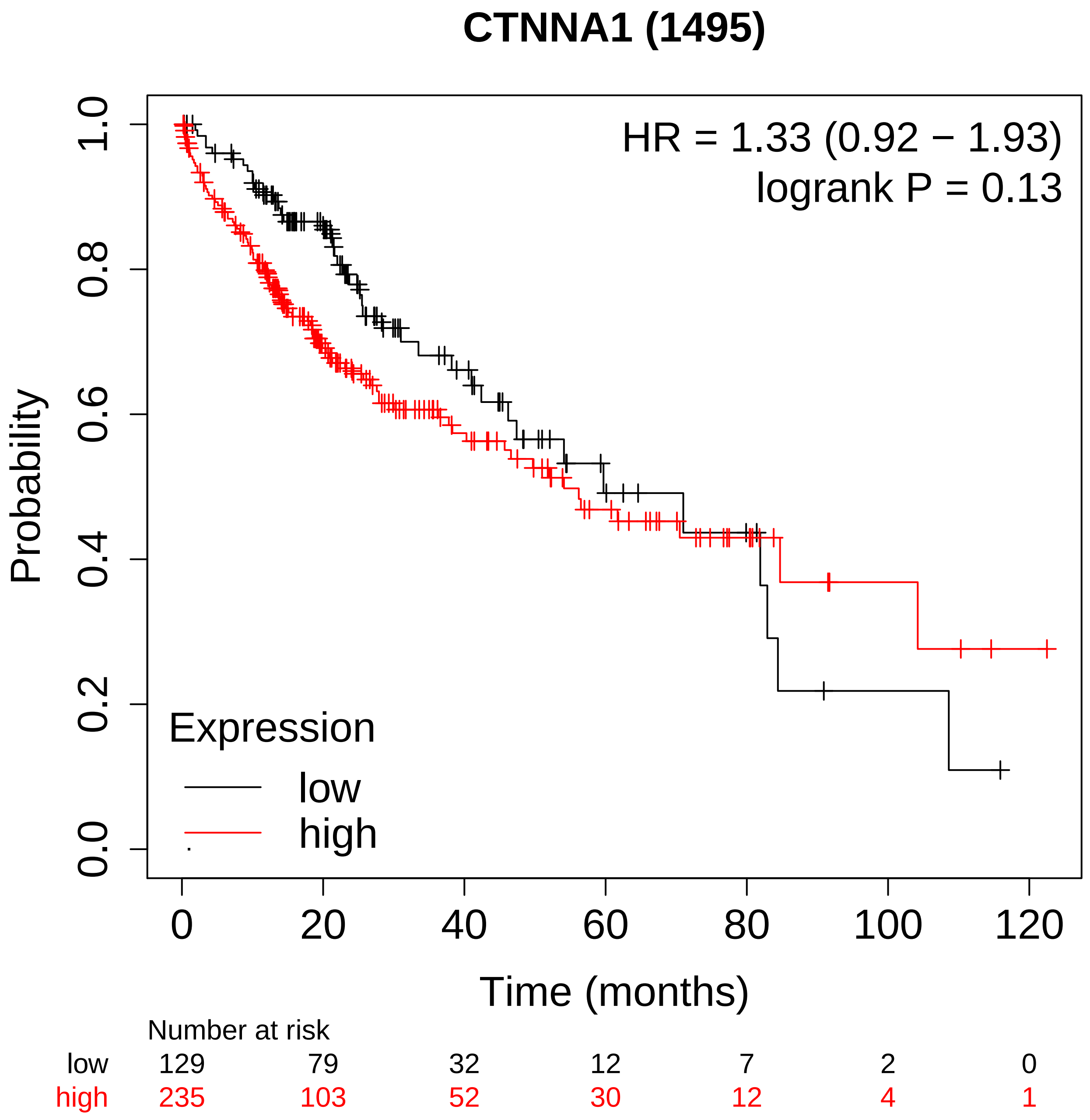
CTNNA1 (1495)
HR = 1.33 (0.92 − 1.93)
logrank P = 0.13
Probability
0.0
0.2
0.4
0.6
0.8
1.0
Expression
low
high
0
20
40
60
80
100
120
Time (months)
Number at risk
low 129 79 32 12 7 2 0
high 235 103 52 30 12 4 1

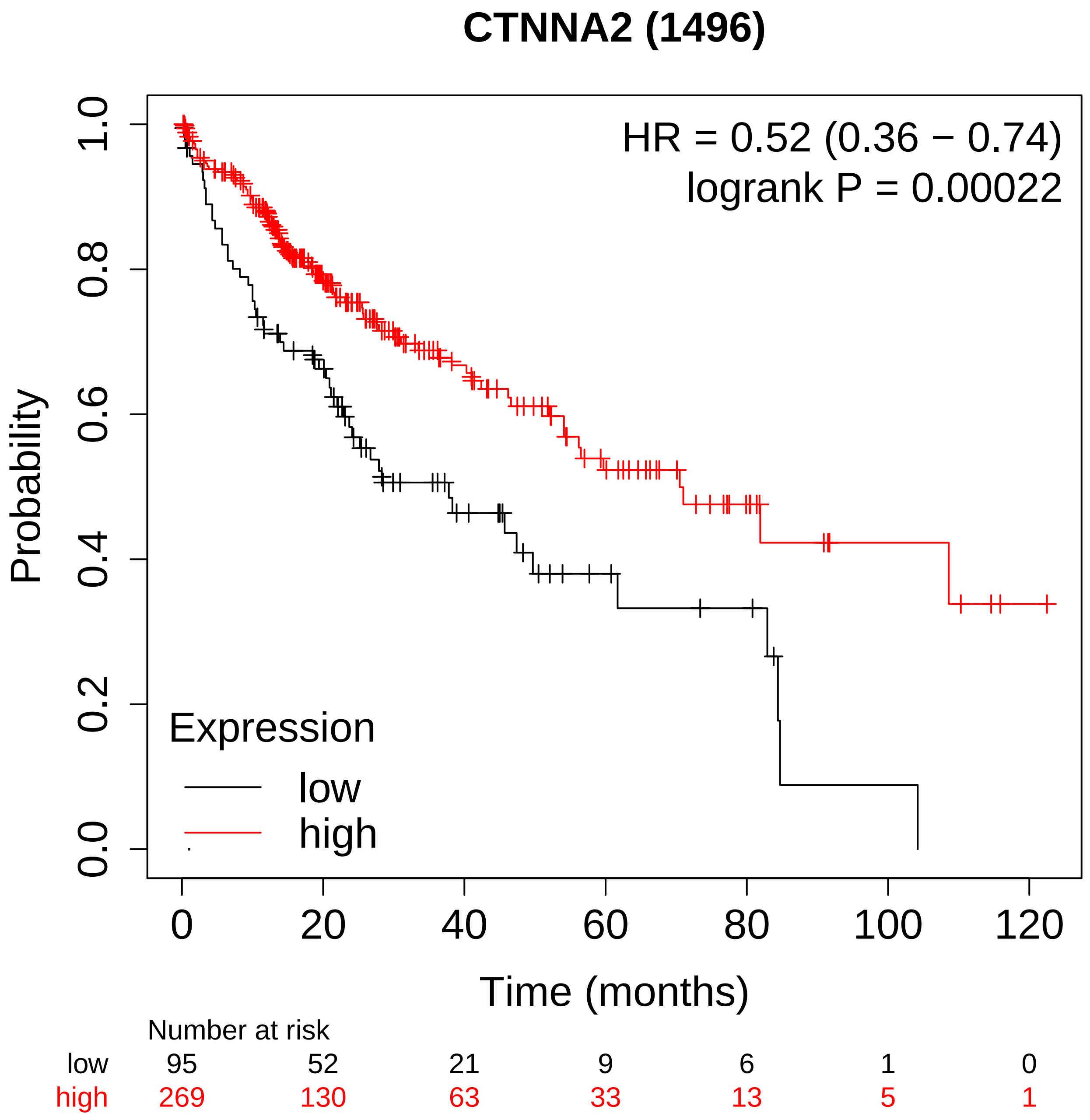

CTNNA2 (1496)
HR = 0.52 (0.36 − 0.74)
logrank P = 0.00022
Probability
0.0
0.2
0.4
0.6
0.8
1.0
Expression
low
high
0
20
40
60
80
100
120
Time (months)
Number at risk
low 95 52 21 9 6 1 0
high 269 130 63 33 13 5 1

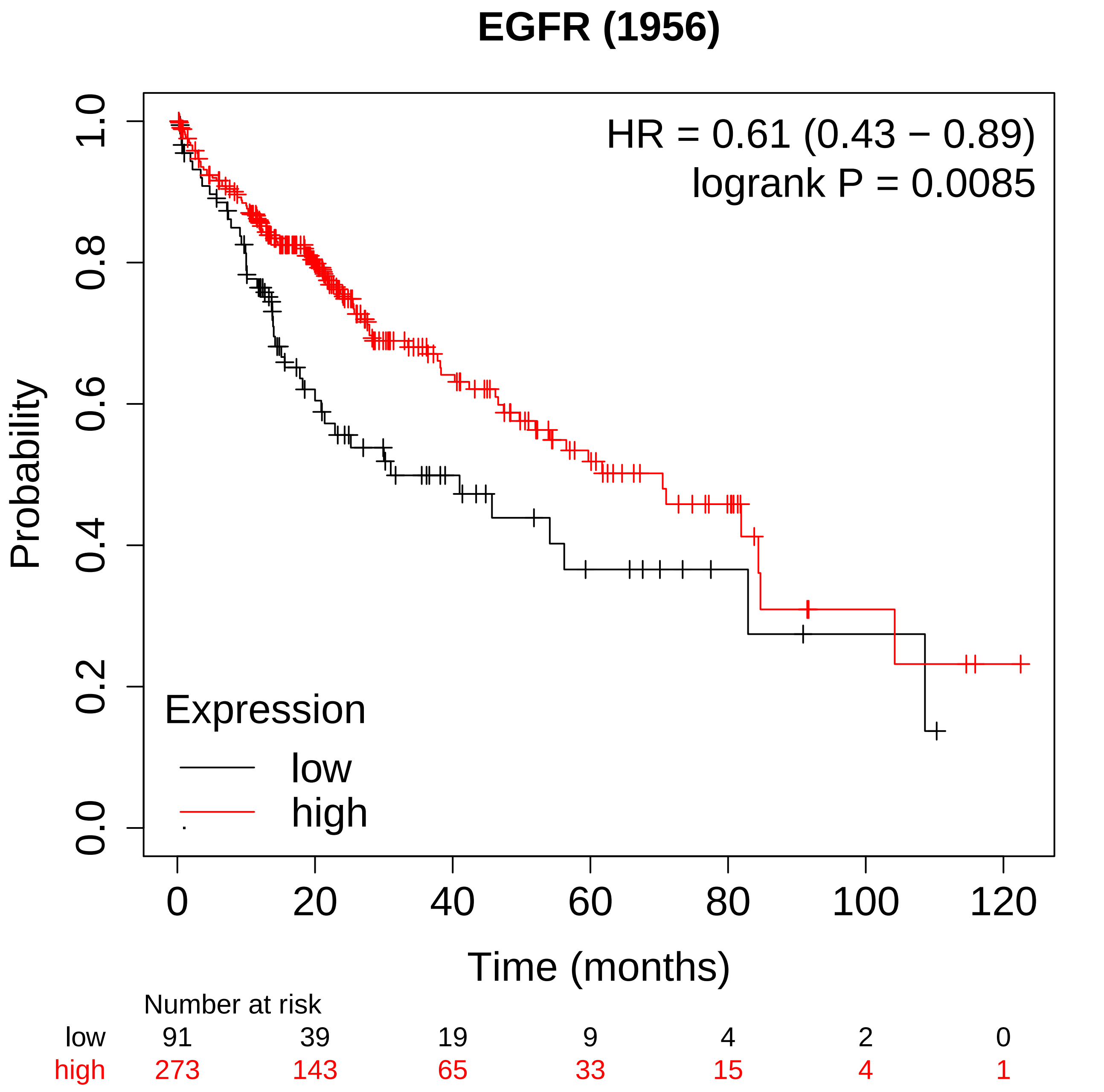
EGFR (1956)
HR = 0.61 (0.43 − 0.89)
logrank P = 0.0085
Probability
1.0
0.8
0.6
0.4
0.2
0.0
Expression
low
high
0
20
40
60
80
100
120
Time (months)
Number at risk
low 91 39 19 9 4 2 0
high 273 143 65 33 15 4 1

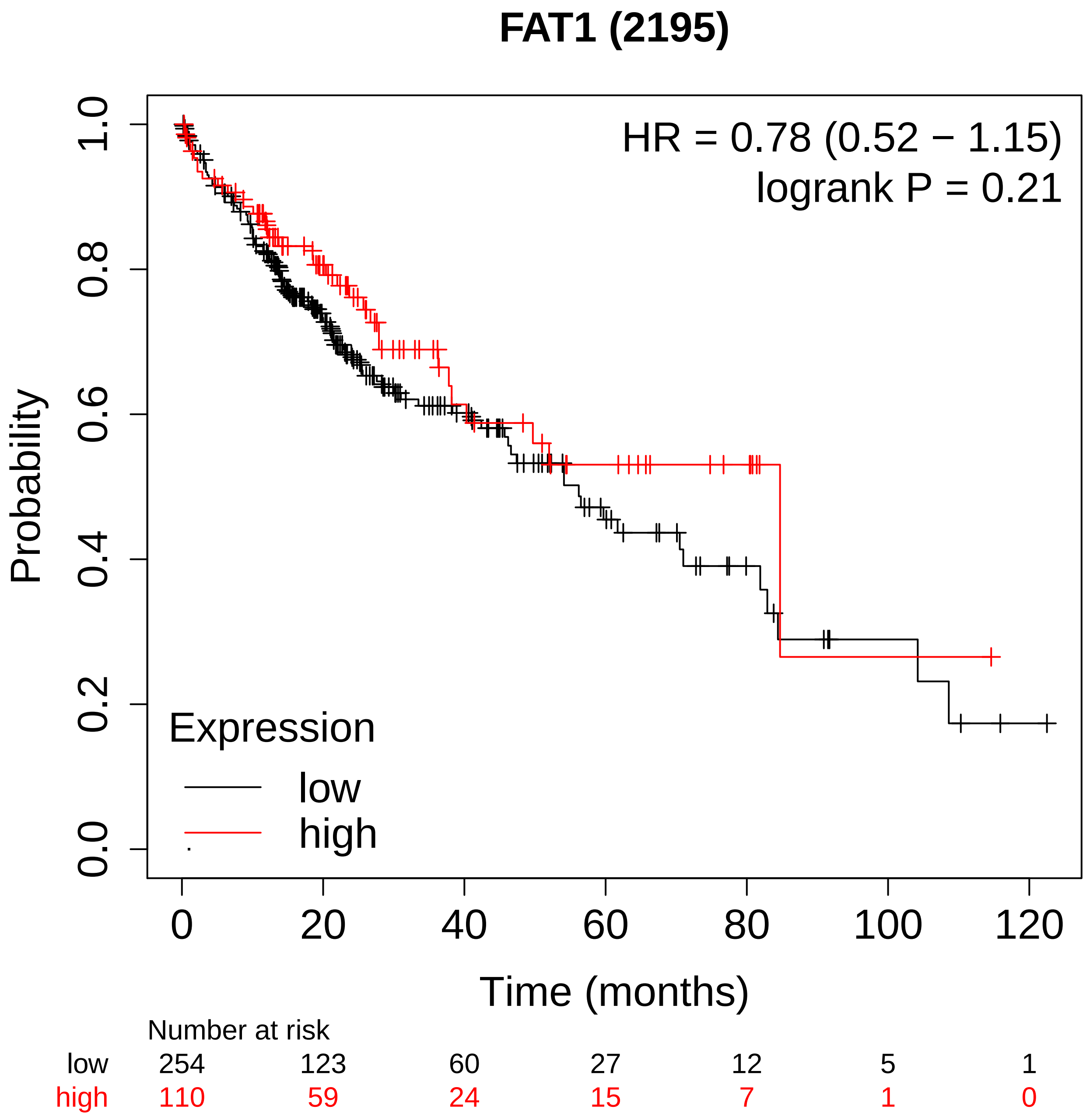
FAT1 (2195)
HR = 0.78 (0.52 − 1.15)
logrank P = 0.21
Probability
0.0
0.2
0.4
0.6
0.8
1.0
Expression
low
high
0
20
40
60
80
100
120
Time (months)
Number at risk
low 254 123 60 27 12 5 1
high 110 59 24 15 7 1 0

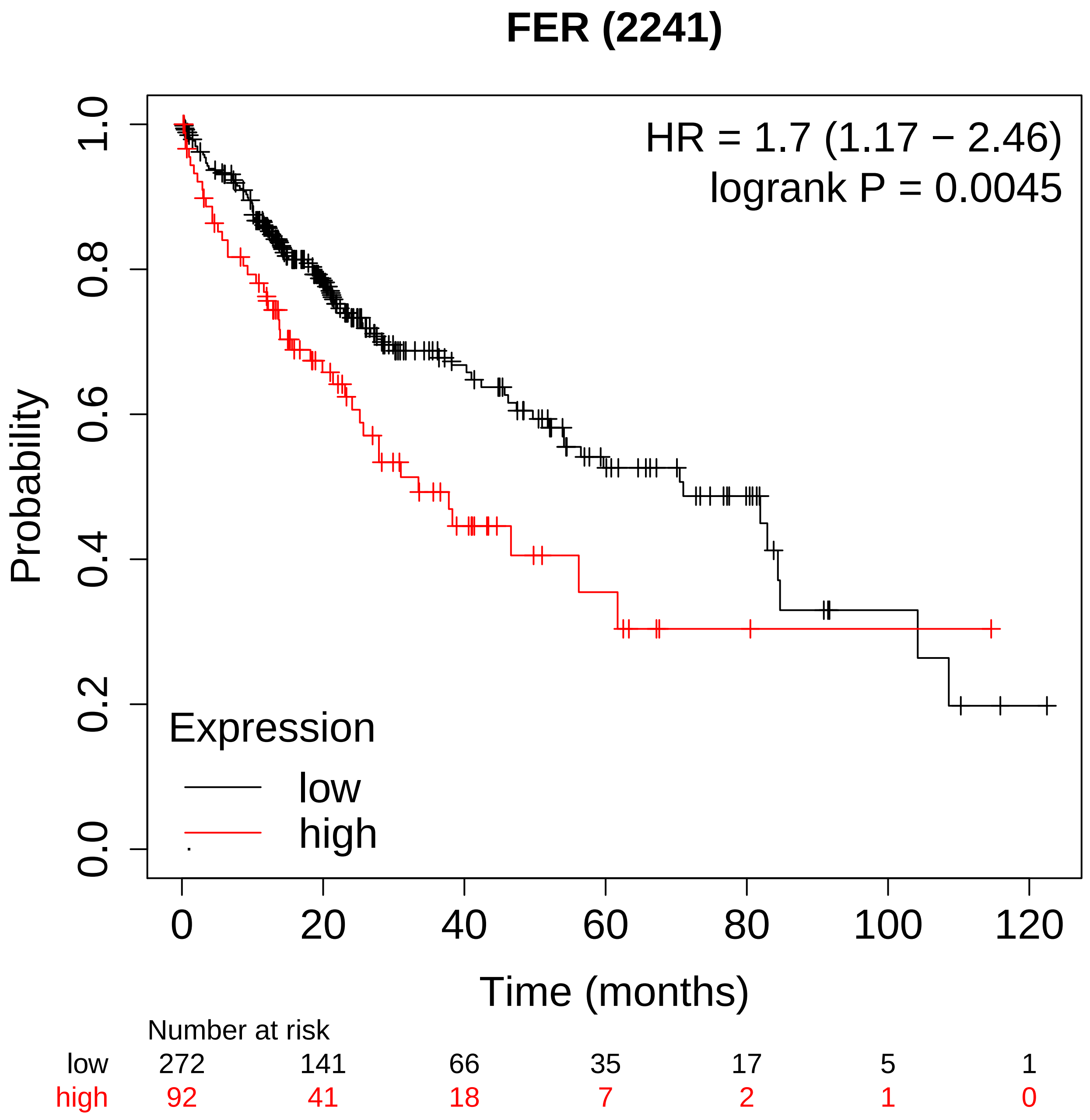
FER (2241)
HR = 1.7 (1.17 − 2.46)
logrank P = 0.0045
Probability
0.0
0.2
0.4
0.6
0.8
1.0
Expression
low
high
0
20
40
60
80
100
120
Time (months)
Number at risk
low 272 141 66 35 17 5 1
high 92 41 18 7 2 1 0

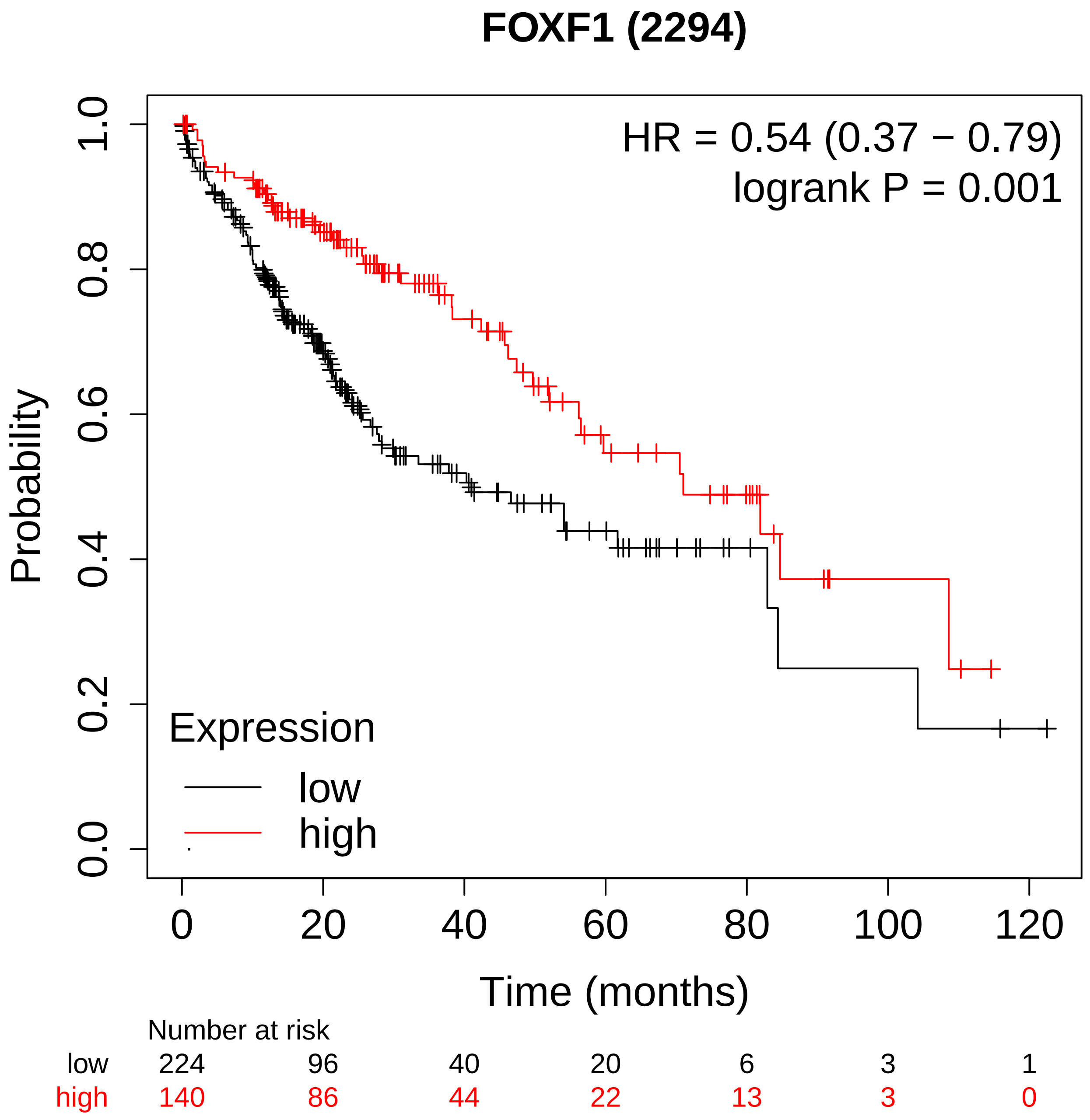

FOXF1 (2294)
HR = 0.54 (0.37 − 0.79)
logrank P = 0.001
Probability
0.0
0.2
0.4
0.6
0.8
1.0
Expression
low
high
0
20
40
60
80
100
120
Time (months)
Number at risk
low 224 96 40 20 6 3 1
high 140 86 44 22 13 3 0

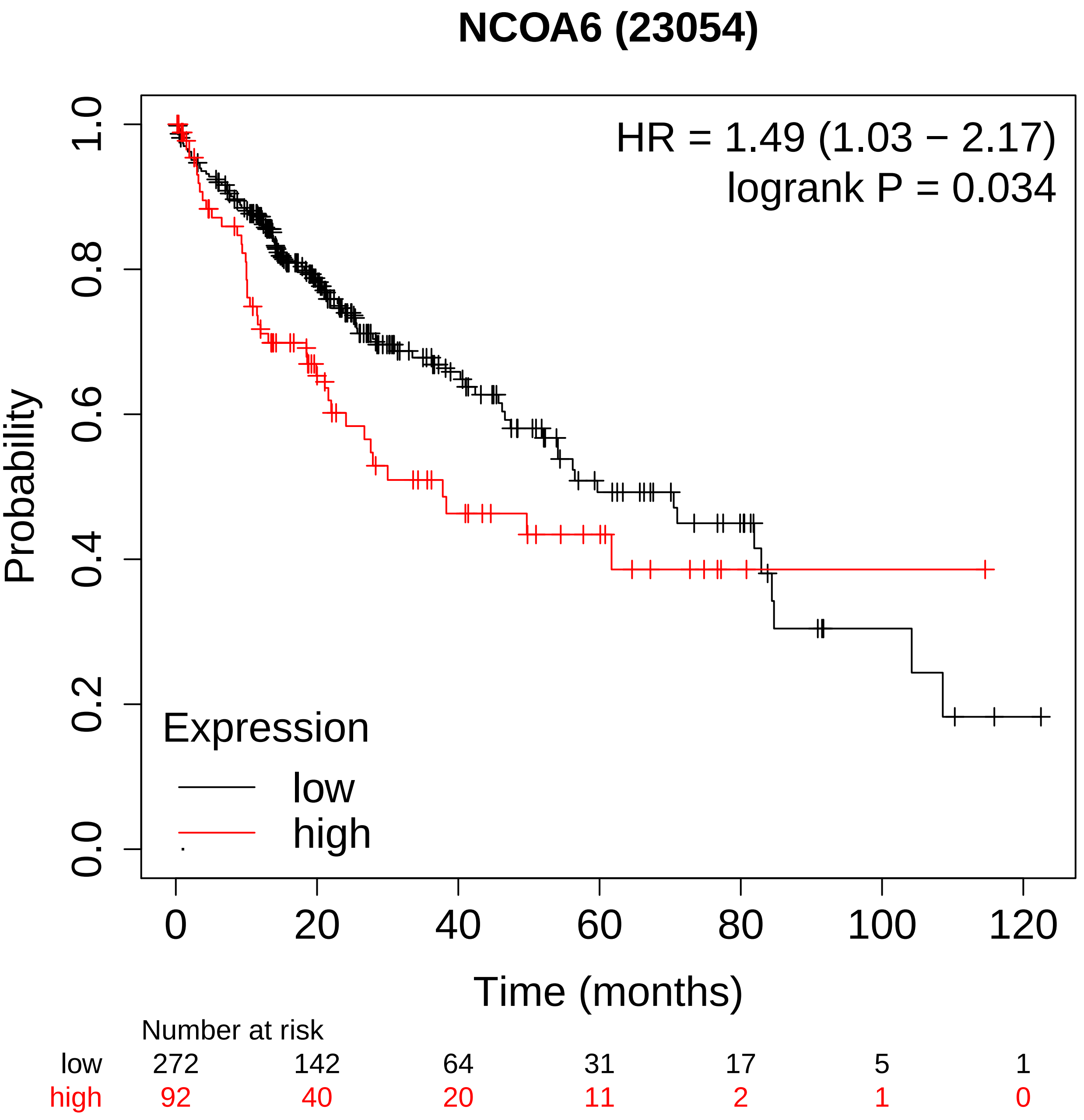
NCOA6 (23054)
HR = 1.49 (1.03 − 2.17)
logrank P = 0.034
Probability
1.0
0.8
0.6
0.4
0.2
0.0
Expression
low
high
0
20
40
60
80
100
120
Time (months)
Number at risk
low 272 142 64 31 17 5 1
high 92 40 20 11 2 1 0

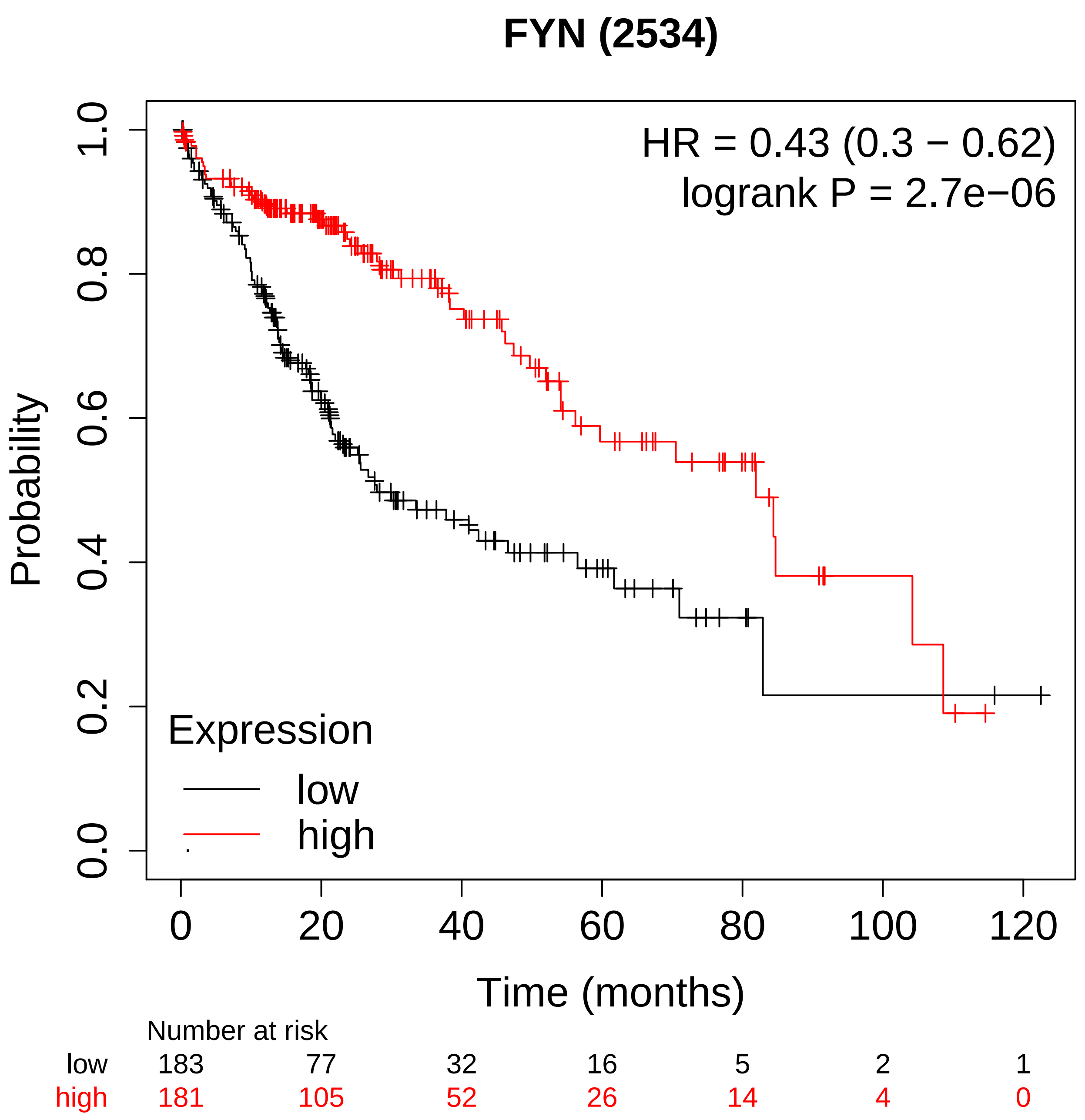

FYN (2534)
HR = 0.43 (0.3 − 0.62)
logrank P = 2.7e−06
Probability
0.0
0.2
0.4
0.6
0.8
1.0
Expression
low
high
0
20
40
60
80
100
120
Time (months)
Number at risk
low 183 77 32 16 5 2 1
high 181 105 52 26 14 4 0

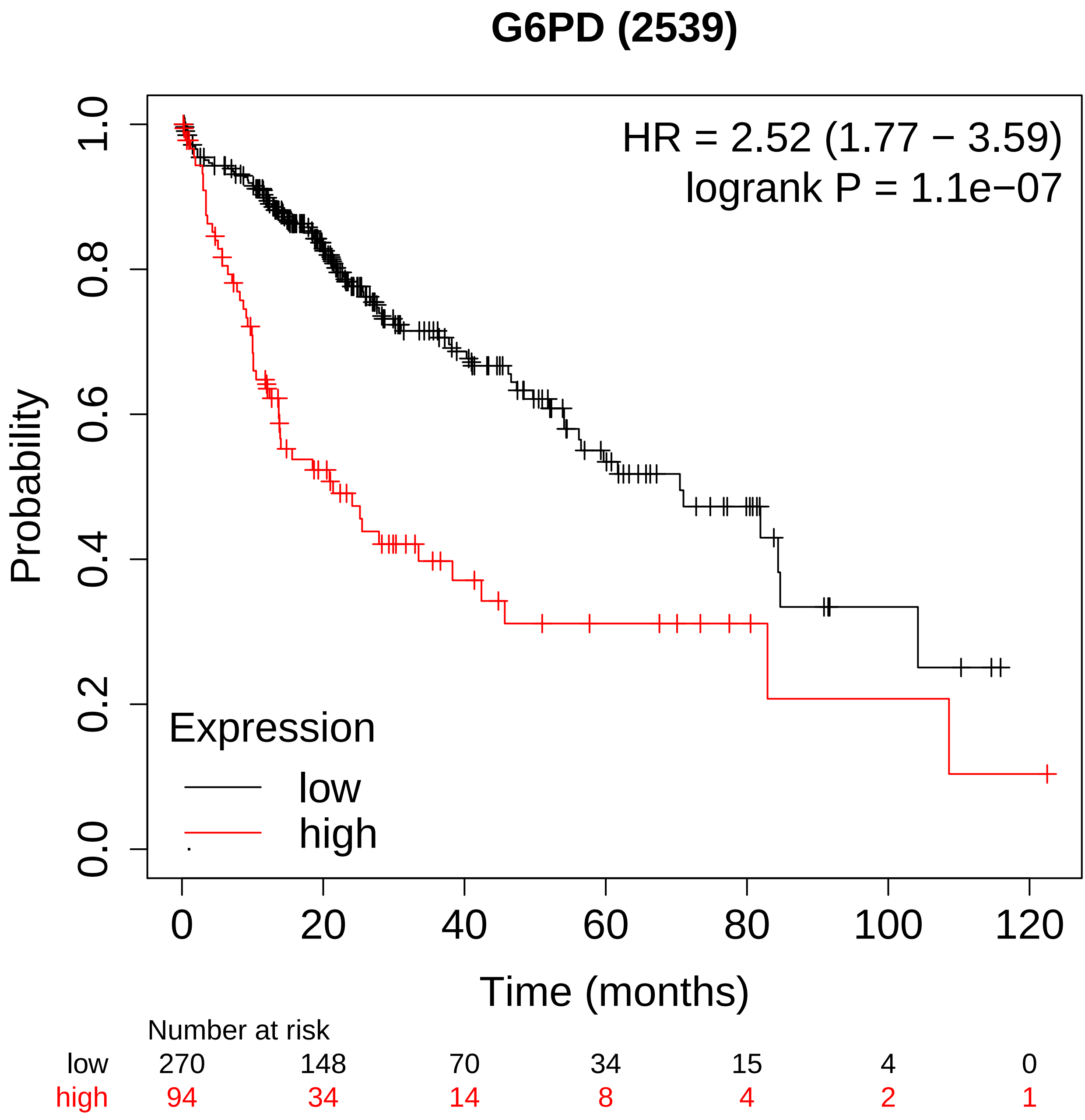
G6PD (2539)
HR = 2.52 (1.77 − 3.59)
logrank P = 1.1e−07
Probability
0.0
0.2
0.4
0.6
0.8
1.0
Expression
low
high
0
20
40
60
80
100
120
Time (months)
Number at risk
low 270 148 70 34 15 4 0
high 94 34 14 8 4 2 1

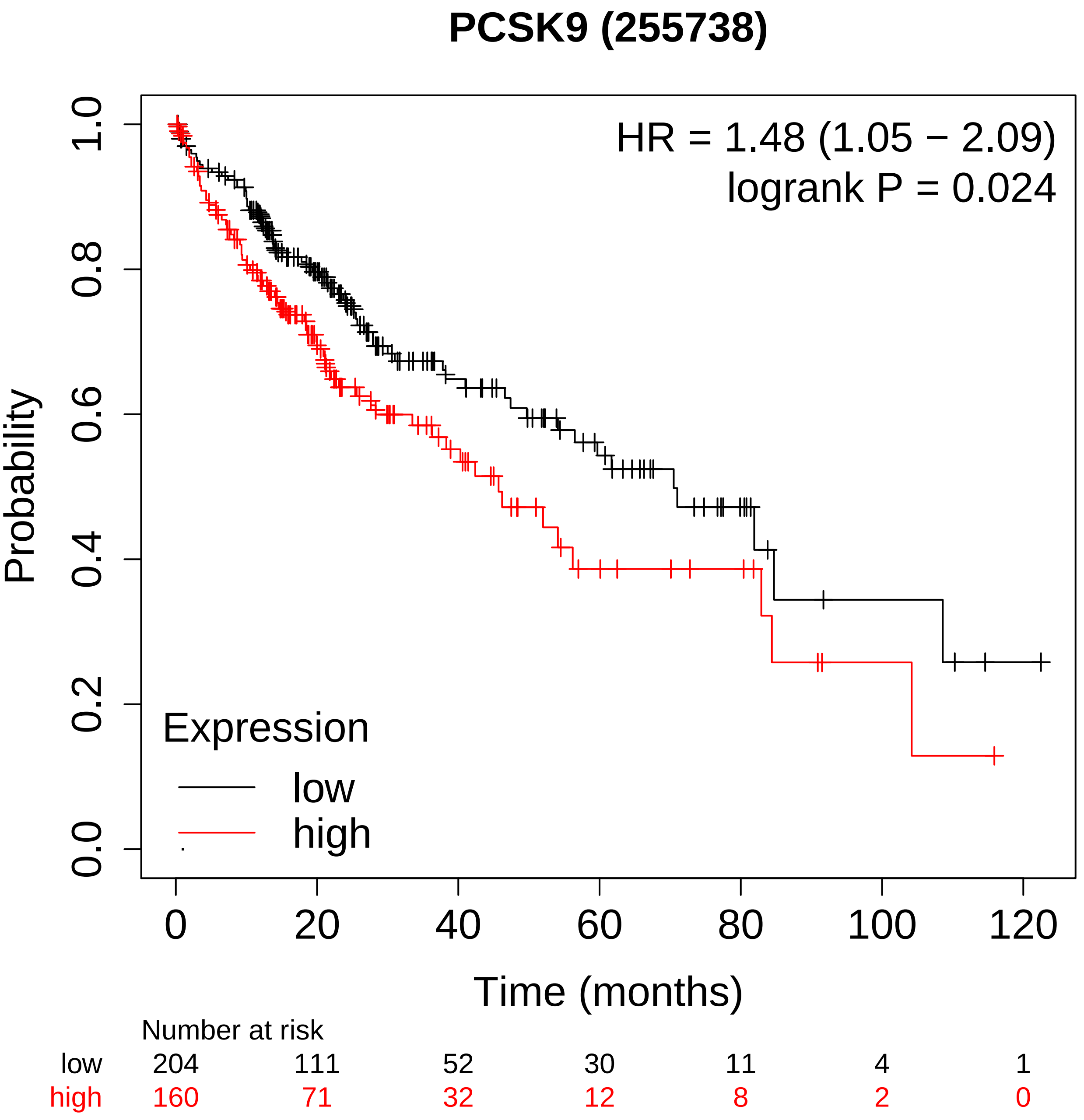

PCSK9 (255738)
HR = 1.48 (1.05 − 2.09)
logrank P = 0.024
Probability
0.0
0.2
0.4
0.6
0.8
1.0
Expression
low
high
0
20
40
60
80
100
120
Time (months)
Number at risk
low 204 111 52 30 11 4 1
high 160 71 32 12 8 2 0

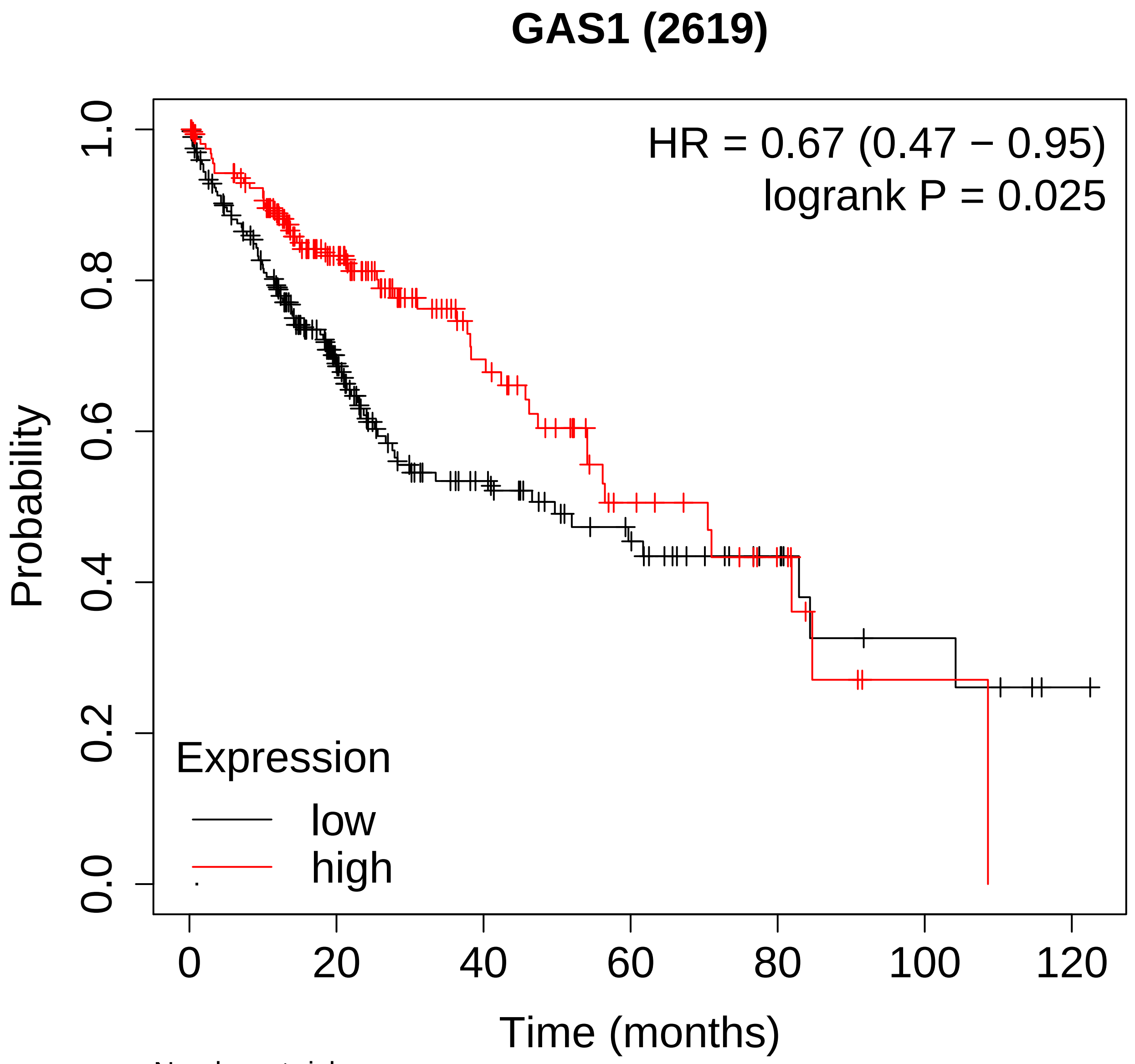

Number at risk

low	202	94	43	24	11	5	1
high	162	88	41	18	8	1	0

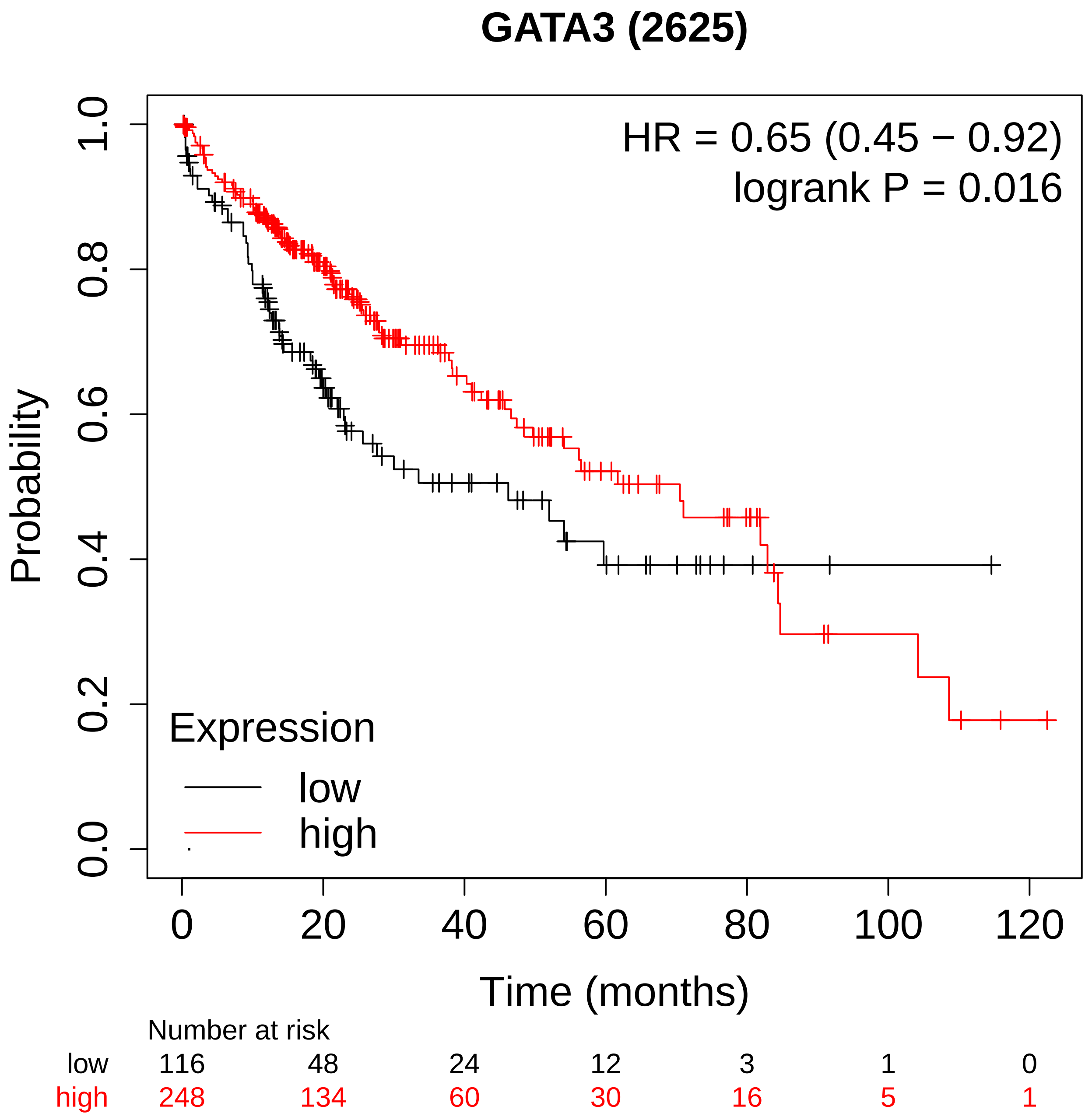

GATA3 (2625)
HR = 0.65 (0.45 − 0.92)
logrank P = 0.016
Probability
0.0
0.2
0.4
0.6
0.8
1.0
Expression
low
high
0
20
40
60
80
100
120
Time (months)
Number at risk
low 116 48 24 12 3 1 0
high 248 134 60 30 16 5 1

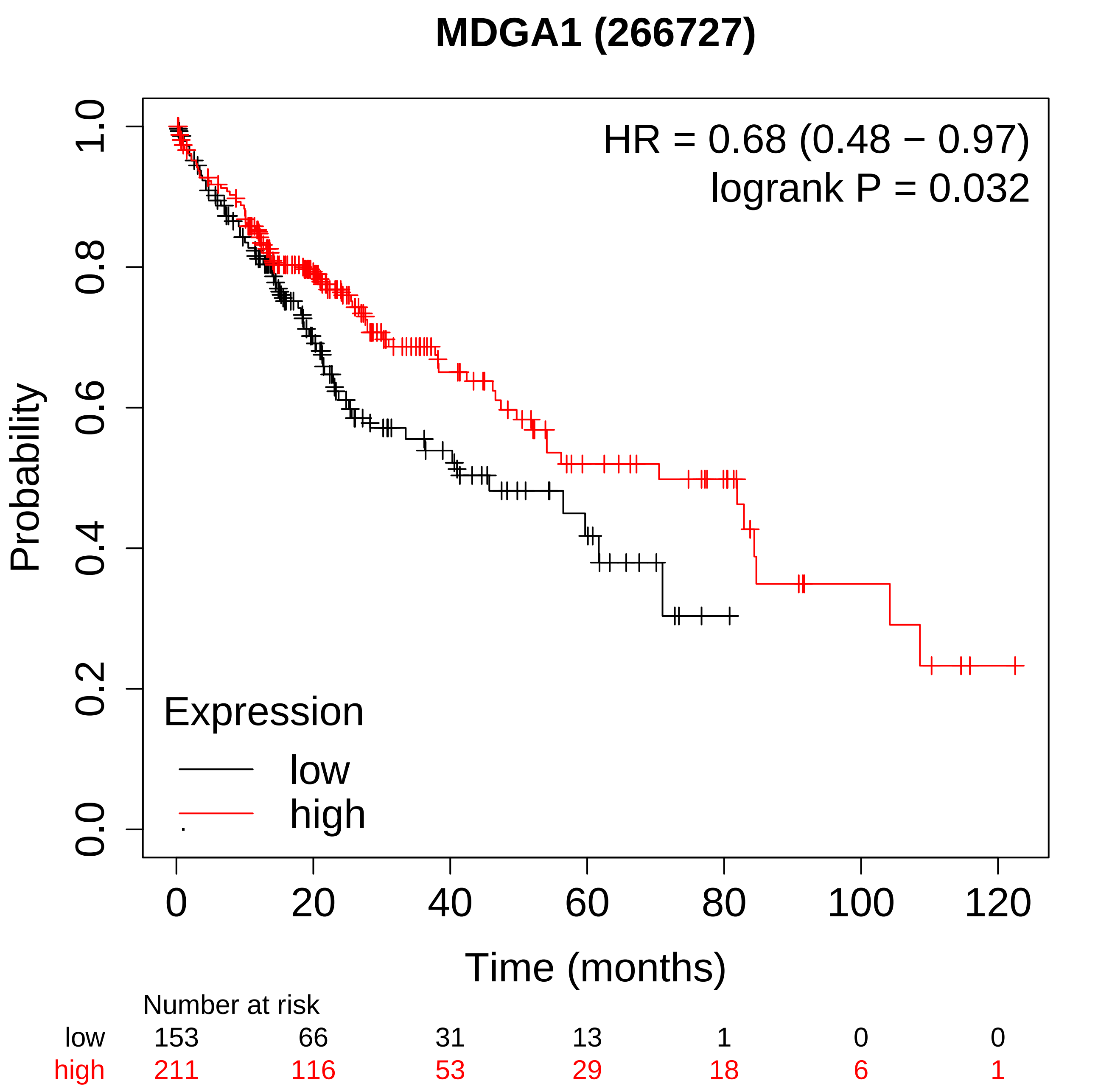

MDGA1 (266727)
HR = 0.68 (0.48 − 0.97)
logrank P = 0.032
Probability
1.0
0.8
0.6
0.4
0.2
0.0
Expression
low
high
0
20
40
60
80
100
120
Time (months)
Number at risk
low 153 66 31 13 1 0 0
high 211 116 53 29 18 6 1

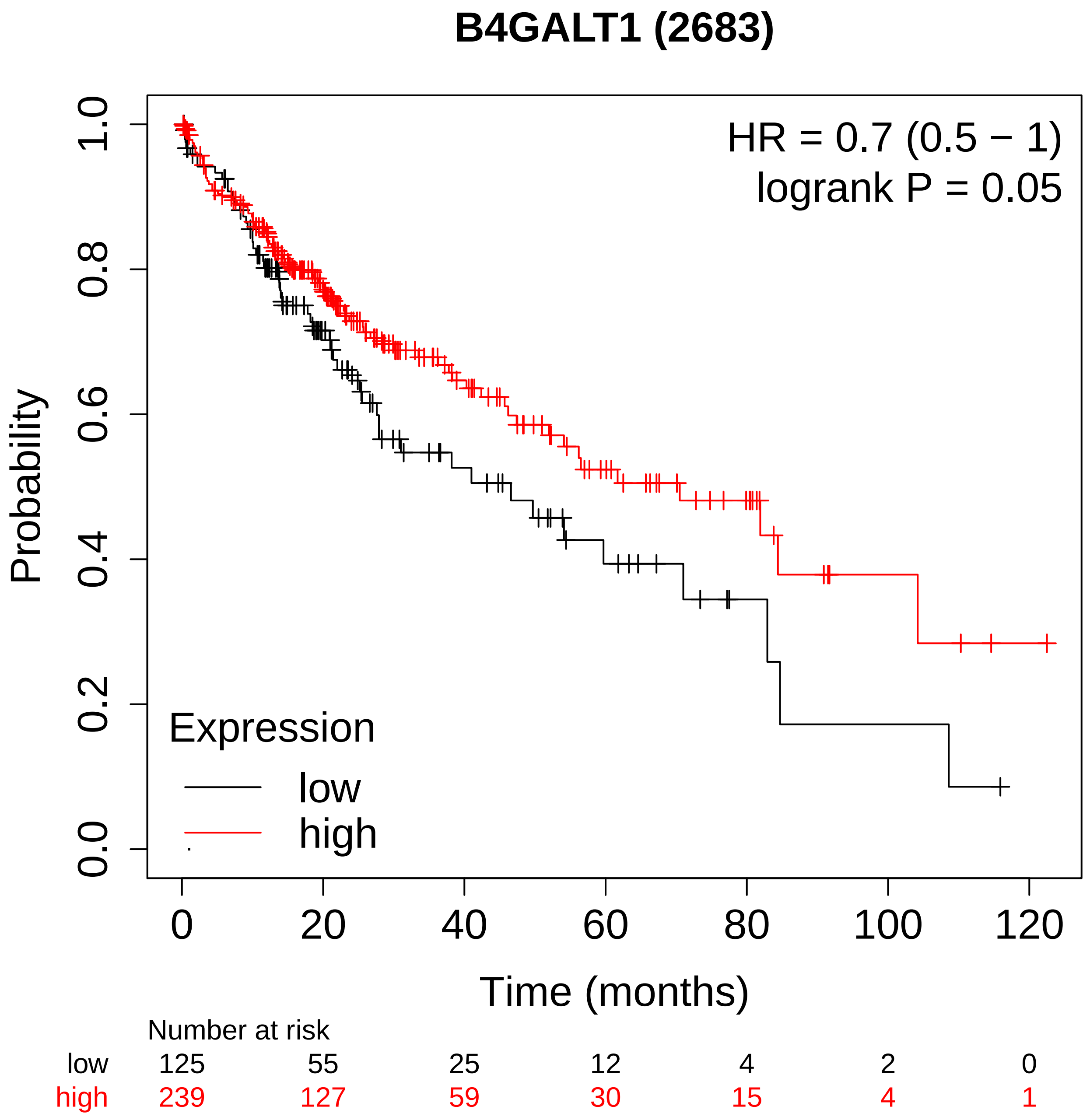
B4GALT1 (2683)
HR = 0.7 (0.5 − 1)
logrank P = 0.05
Probability
0.0
0.2
0.4
0.6
0.8
1.0
Expression
low
high
0
20
40
60
80
100
120
Time (months)
Number at risk
low 125 55 25 12 4 2 0
high 239 127 59 30 15 4 1

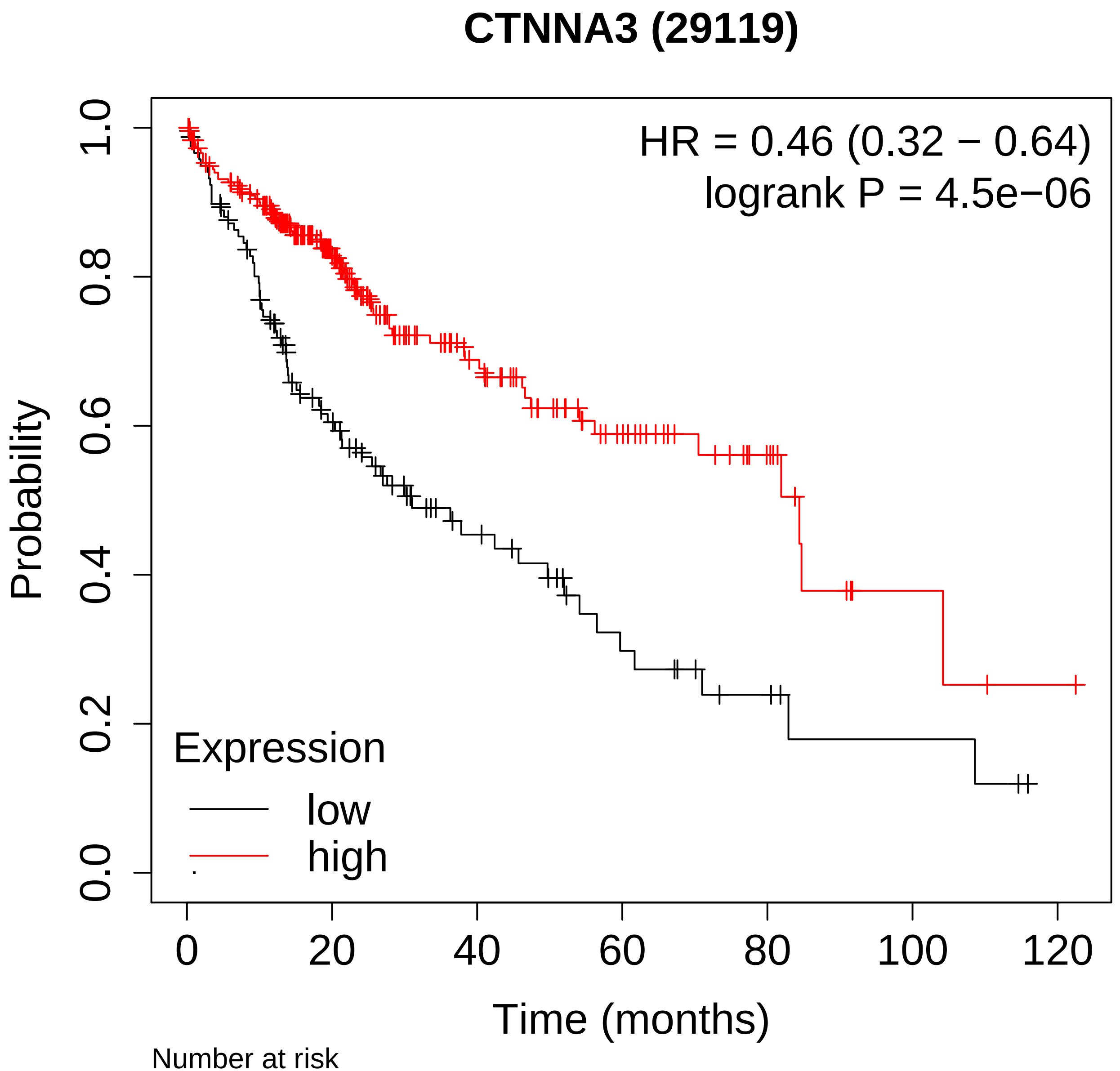

Number at risk

low	122	54	25	12	6	3	0
high	242	128	59	30	13	3	1

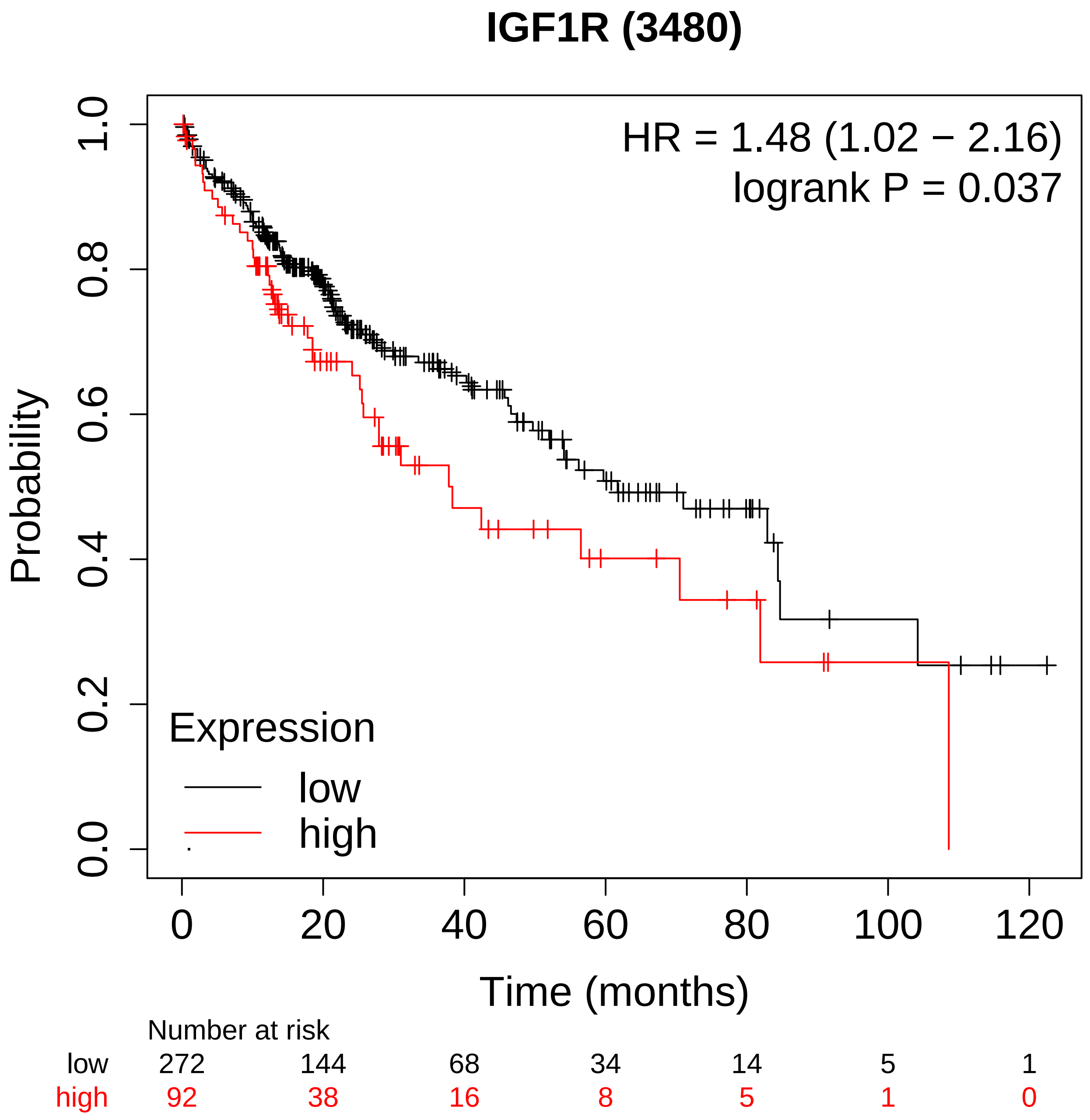

IGF1R (3480)
HR = 1.48 (1.02 − 2.16)
logrank P = 0.037
Probability
0.0
0.2
0.4
0.6
0.8
1.0
Expression
low
high
0
20
40
60
80
100
120
Time (months)
Number at risk
low 272 144 68 34 14 5 1
high 92 38 16 8 5 1 0

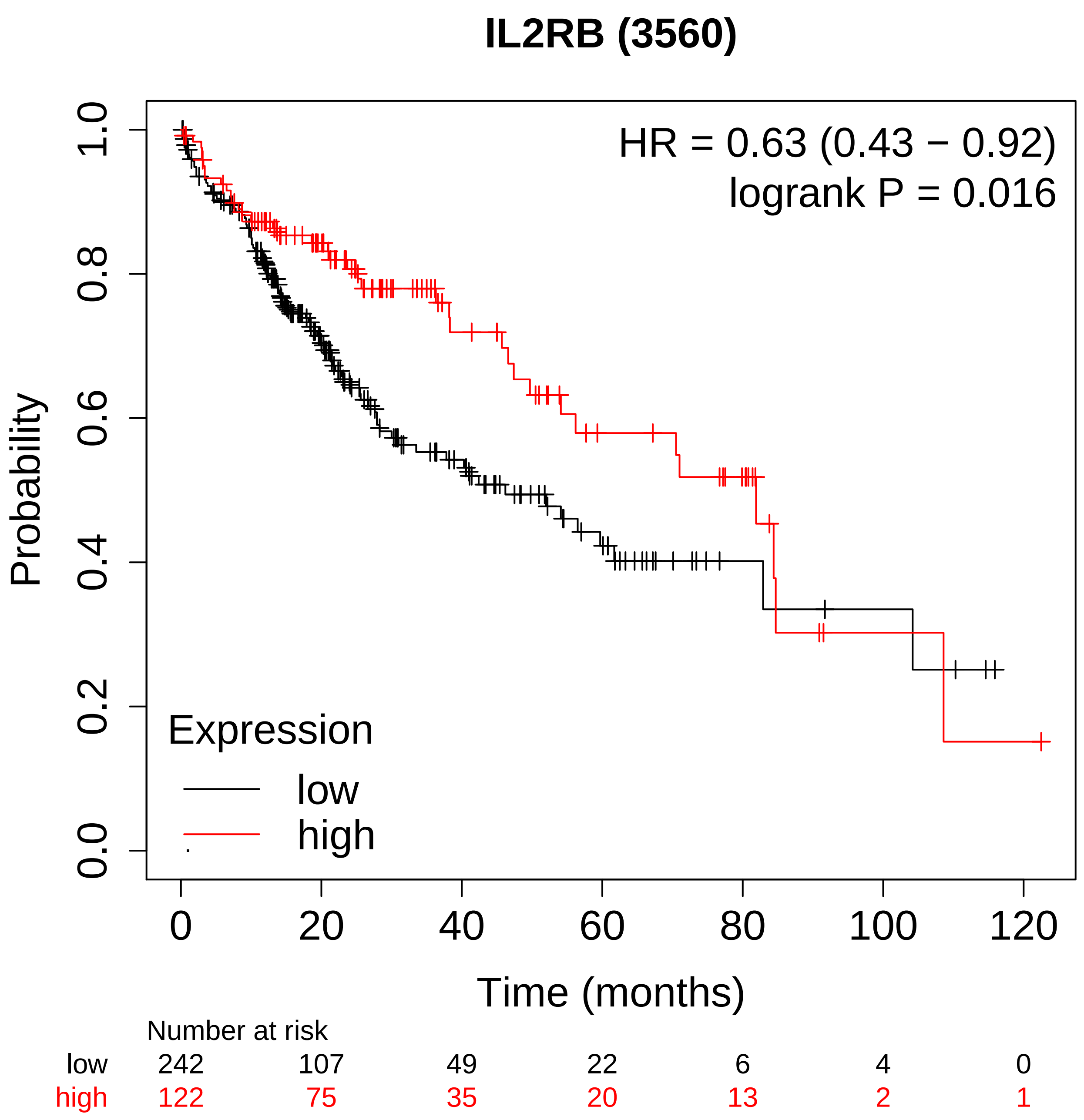
IL2RB (3560)
HR = 0.63 (0.43 − 0.92)
logrank P = 0.016
Probability
0.0
0.2
0.4
0.6
0.8
1.0
Expression
low
high
0
20
40
60
80
100
120
Time (months)
Number at risk
low 242 107 49 22 6 4 0
high 122 75 35 20 13 2 1

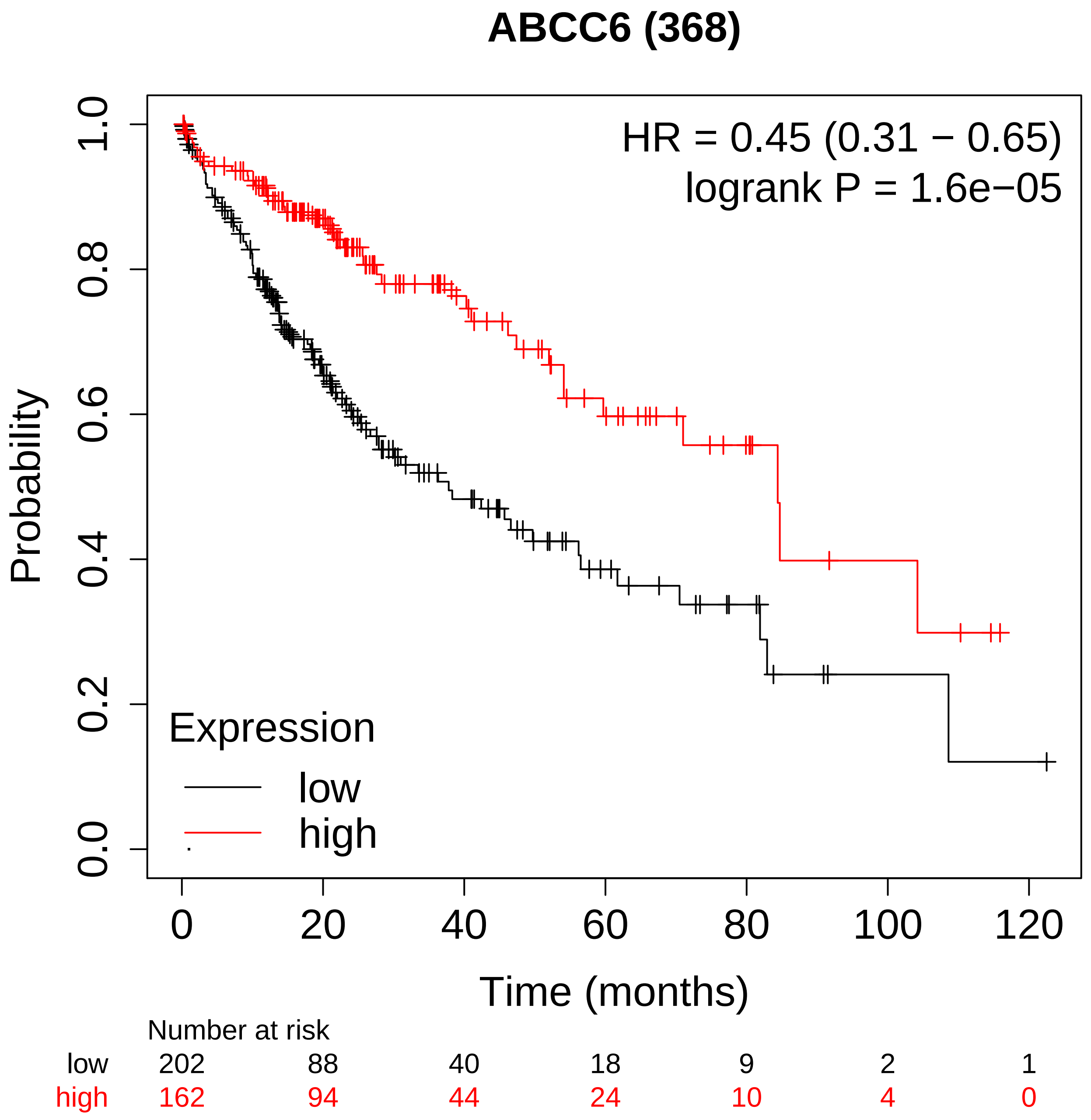
ABCC6 (368)
HR = 0.45 (0.31 − 0.65)
logrank P = 1.6e−05
Probability
1.0
0.8
0.6
0.4
0.2
0.0
Expression
low
high
0
20
40
60
80
100
120
Time (months)
Number at risk
low 202 88 40 18 9 2 1
high 162 94 44 24 10 4 0

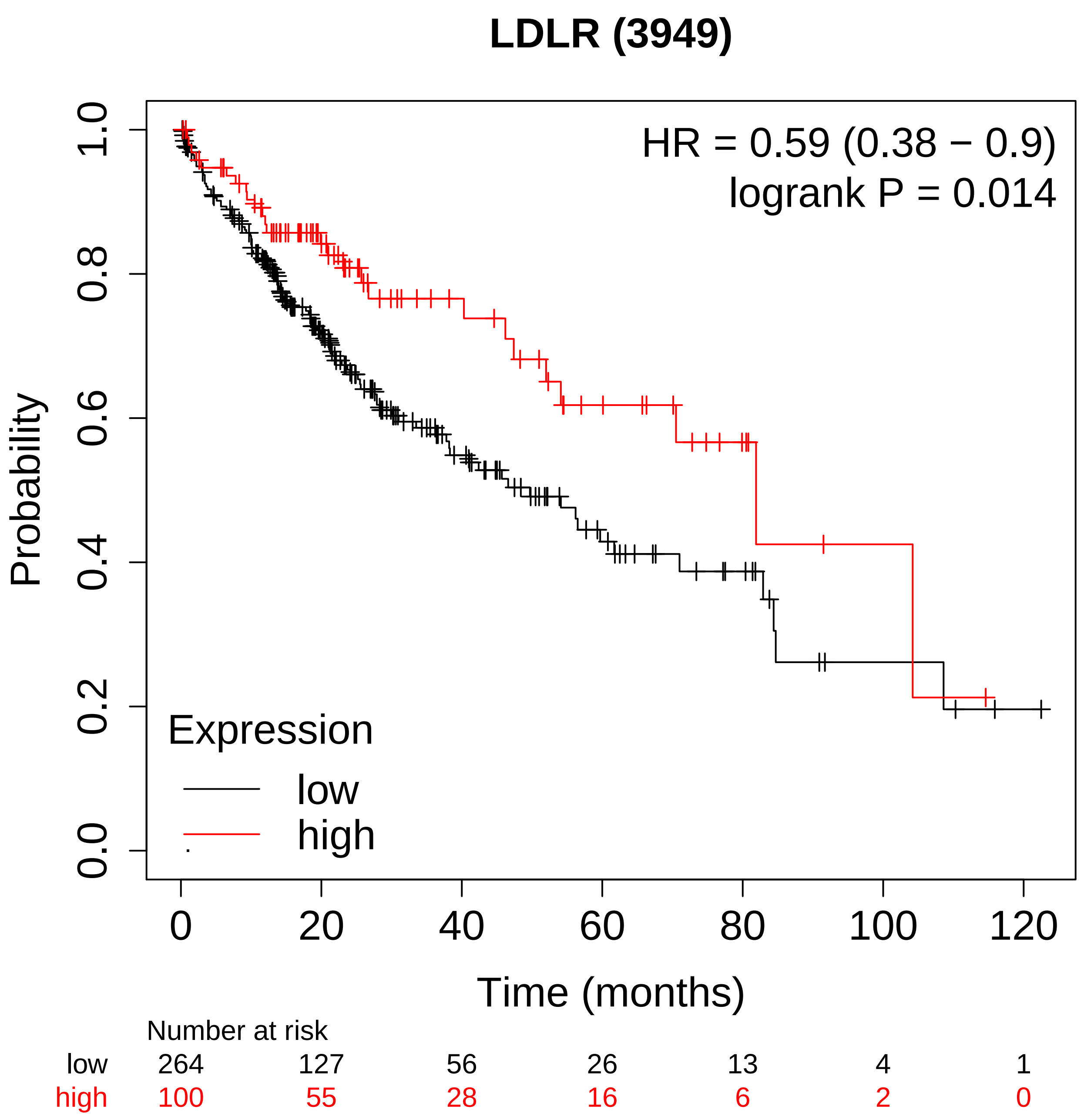

LDLR (3949)
HR = 0.59 (0.38 − 0.9)
logrank P = 0.014
Probability
1.0
0.8
0.6
0.4
0.2
0.0
Expression
low
high
0
20
40
60
80
100
120
Time (months)
Number at risk
low 264 127 56 26 13 4 1
high 100 55 28 16 6 2 0

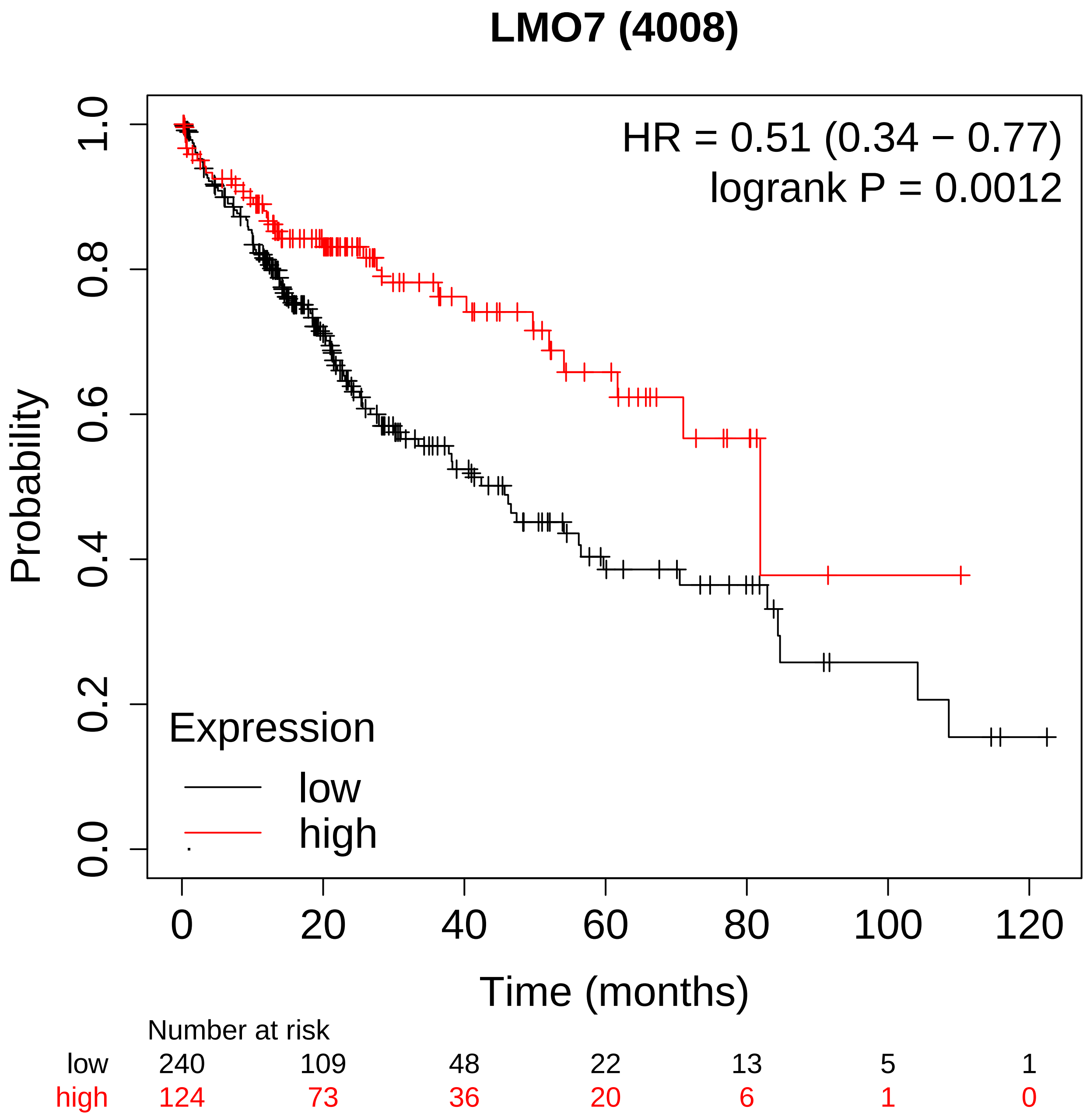

LMO7 (4008)
HR = 0.51 (0.34 − 0.77)
logrank P = 0.0012
Probability
0.0
0.2
0.4
0.6
0.8
1.0
Expression
low
high
0
20
40
60
80
100
120
Time (months)
Number at risk
low 240 109 48 22 13 5 1
high 124 73 36 20 6 1 0

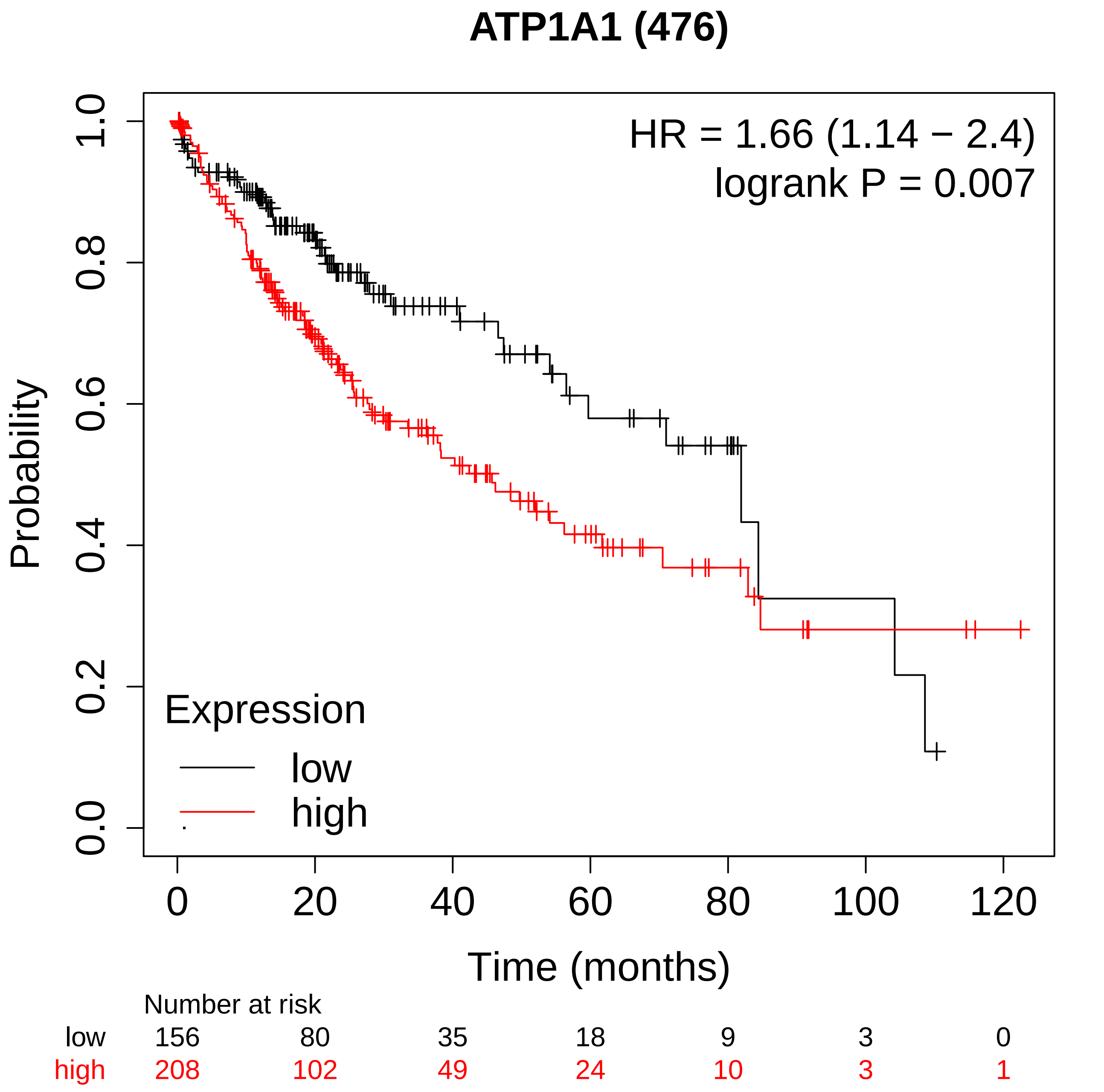

ATP1A1 (476)
HR = 1.66 (1.14 − 2.4)
logrank P = 0.007
Probability
0.0
0.2
0.4
0.6
0.8
1.0
Expression
low
high
0
20
40
60
80
100
120
Time (months)
Number at risk
low 156 80 35 18 9 3 0
high 208 102 49 24 10 3 1

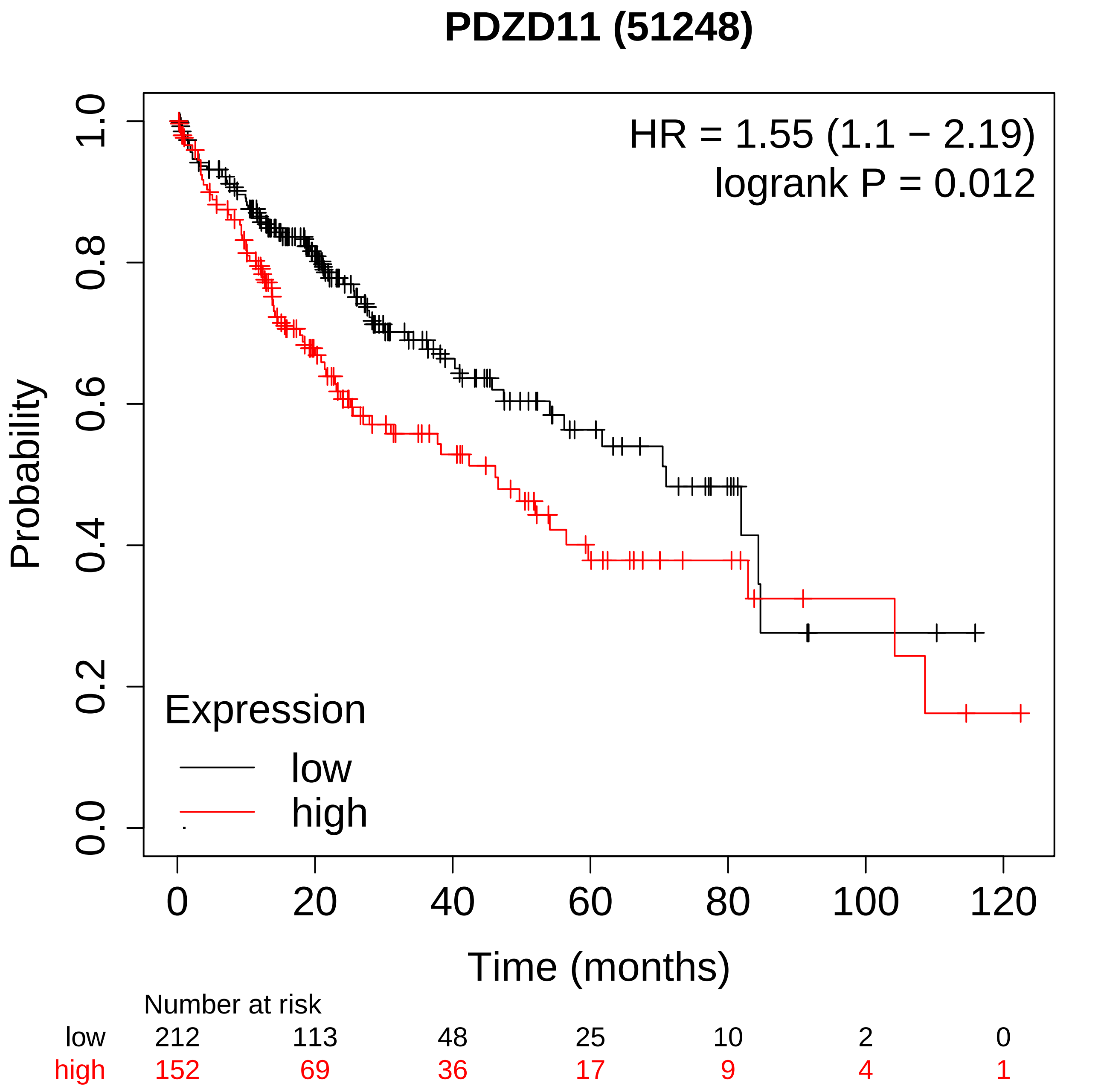
PDZD11 (51248)
HR = 1.55 (1.1 − 2.19)
logrank P = 0.012
Probability
0.0
0.2
0.4
0.6
0.8
1.0
Time (months)
0
20
40
60
80
100
120
Expression
low
high
Number at risk
low 212 113 48 25 10 2 0
high 152 69 36 17 9 4 1

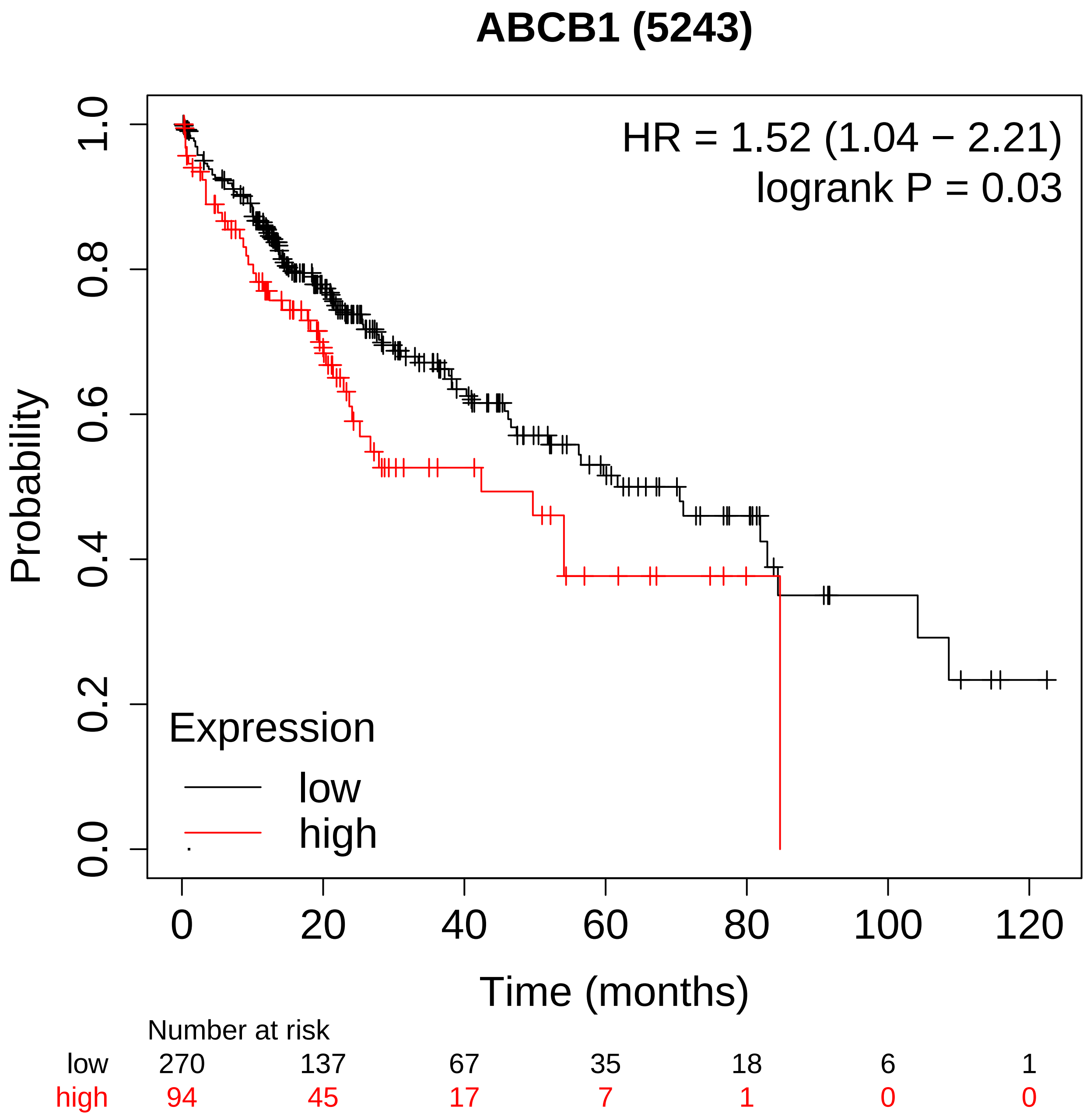
ABCB1 (5243)
HR = 1.52 (1.04 − 2.21)
logrank P = 0.03
Probability
0.0
0.2
0.4
0.6
0.8
1.0
Expression
low
high
0
20
40
60
80
100
120
Time (months)
Number at risk
low 270 137 67 35 18 6 1
high 94 45 17 7 1 0 0

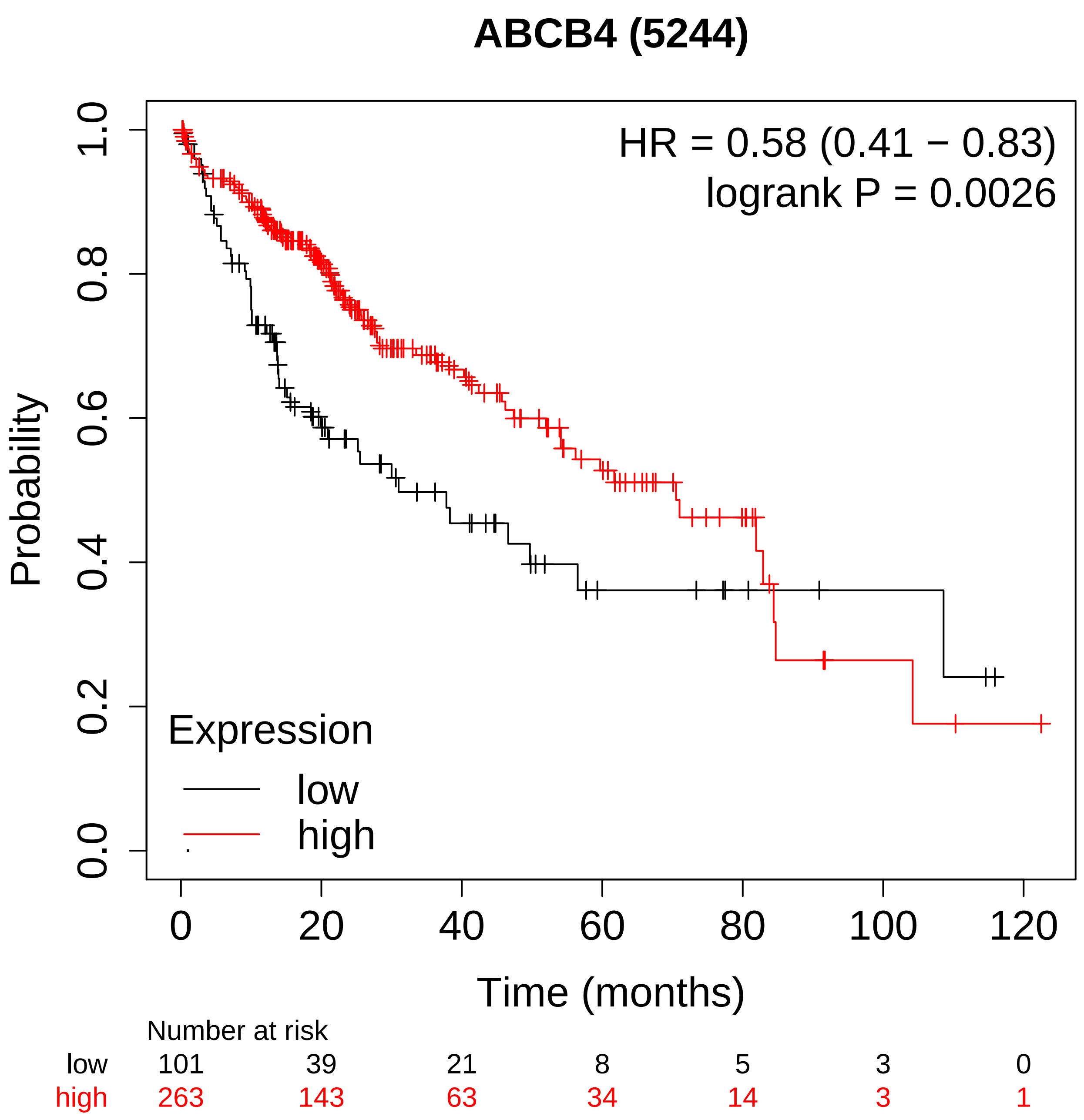
ABCB4 (5244)
HR = 0.58 (0.41 − 0.83)
logrank P = 0.0026
Probability
0.0
0.2
0.4
0.6
0.8
1.0
Expression
low
high
0
20
40
60
80
100
120
Time (months)
Number at risk
low 101 39 21 8 5 3 0
high 263 143 63 34 14 3 1

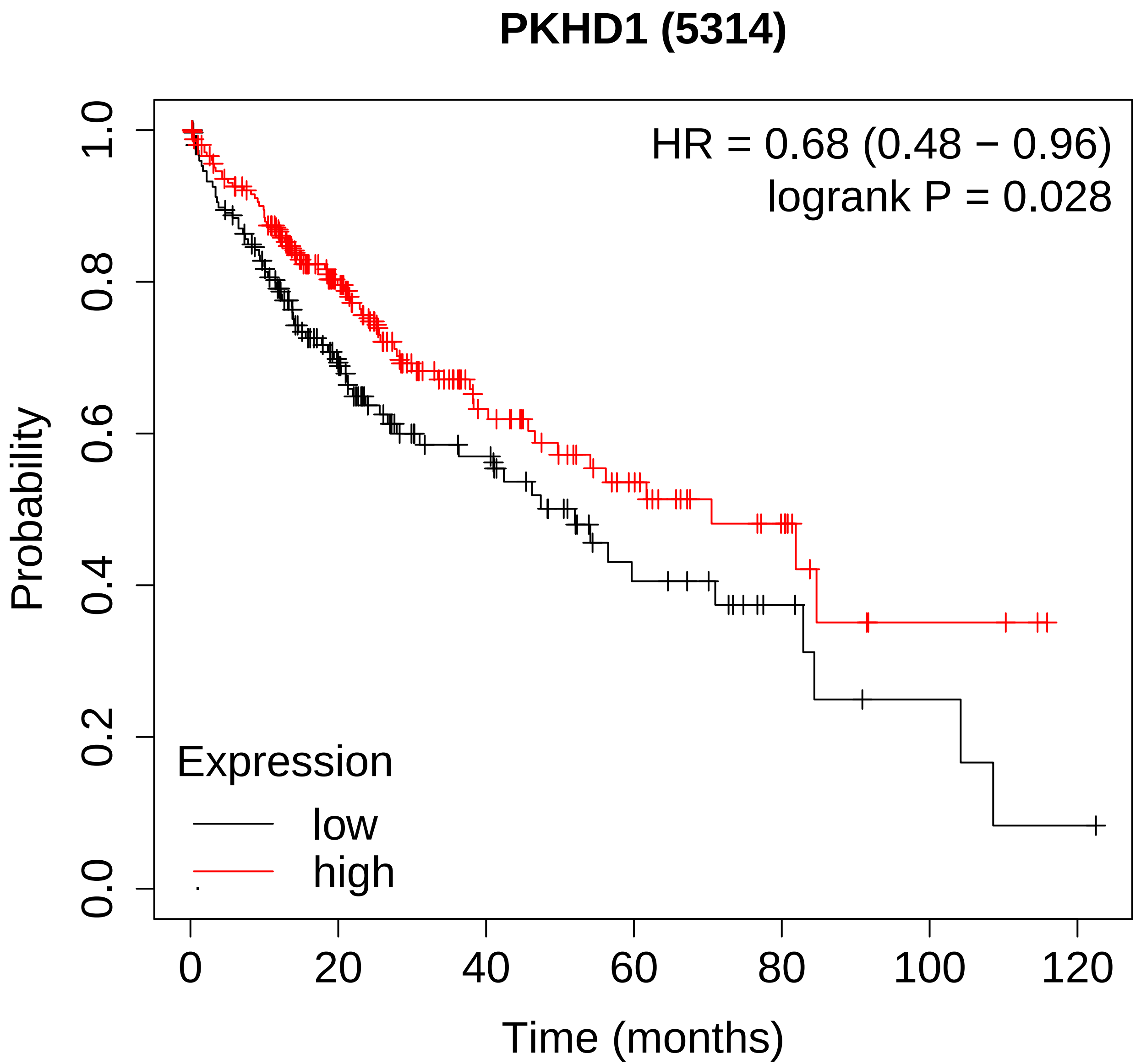

Number at risk

low	154	74	37	16	7	3	1
high	210	108	47	26	12	3	0

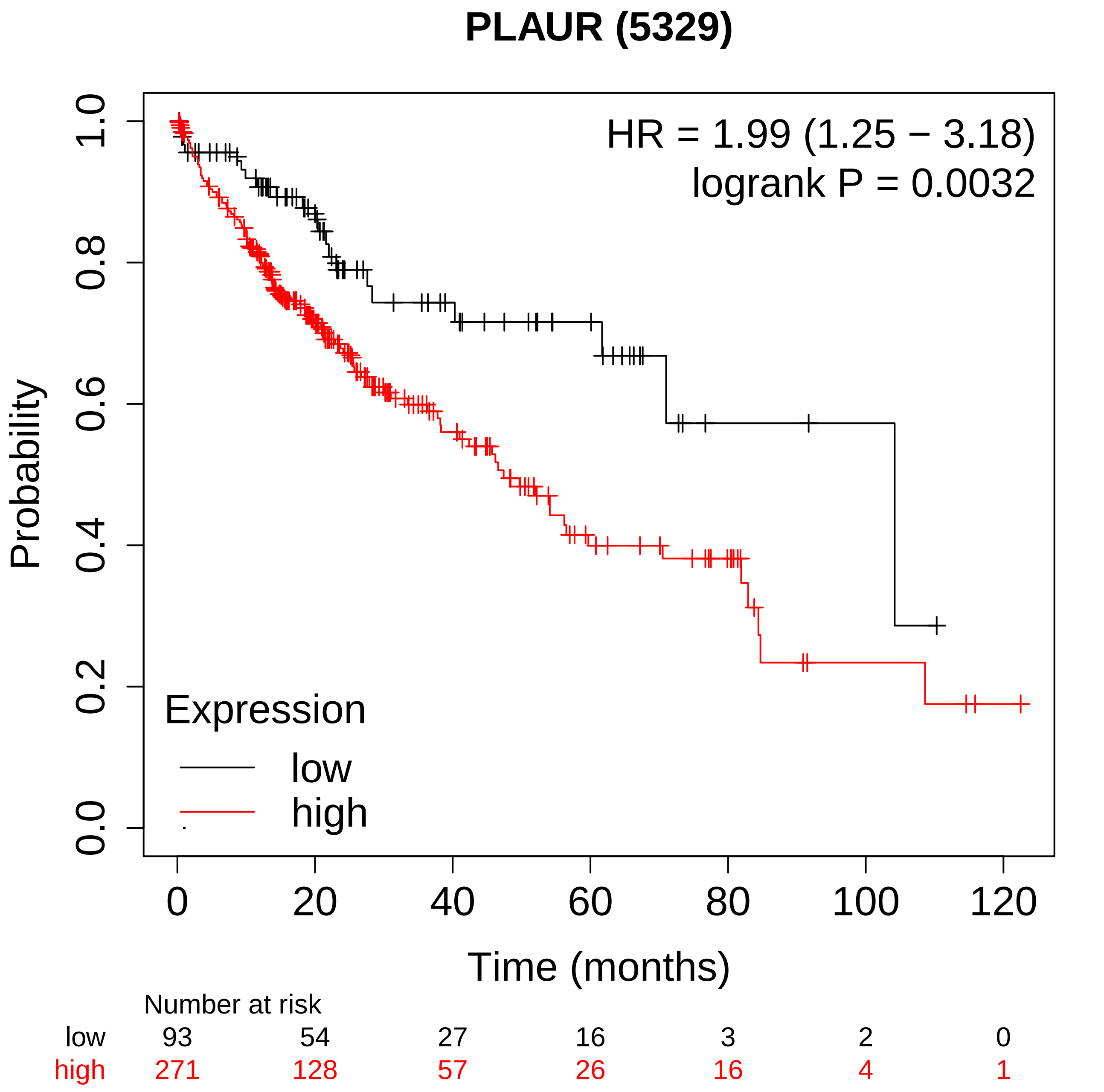
PLAUR (5329)
HR = 1.99 (1.25 − 3.18)
logrank P = 0.0032
Probability
1.0
0.8
0.6
0.4
0.2
0.0
Expression
low
high
0
20
40
60
80
100
120
Time (months)
Number at risk
low 93 54 27 16 3 2 0
high 271 128 57 26 16 4 1

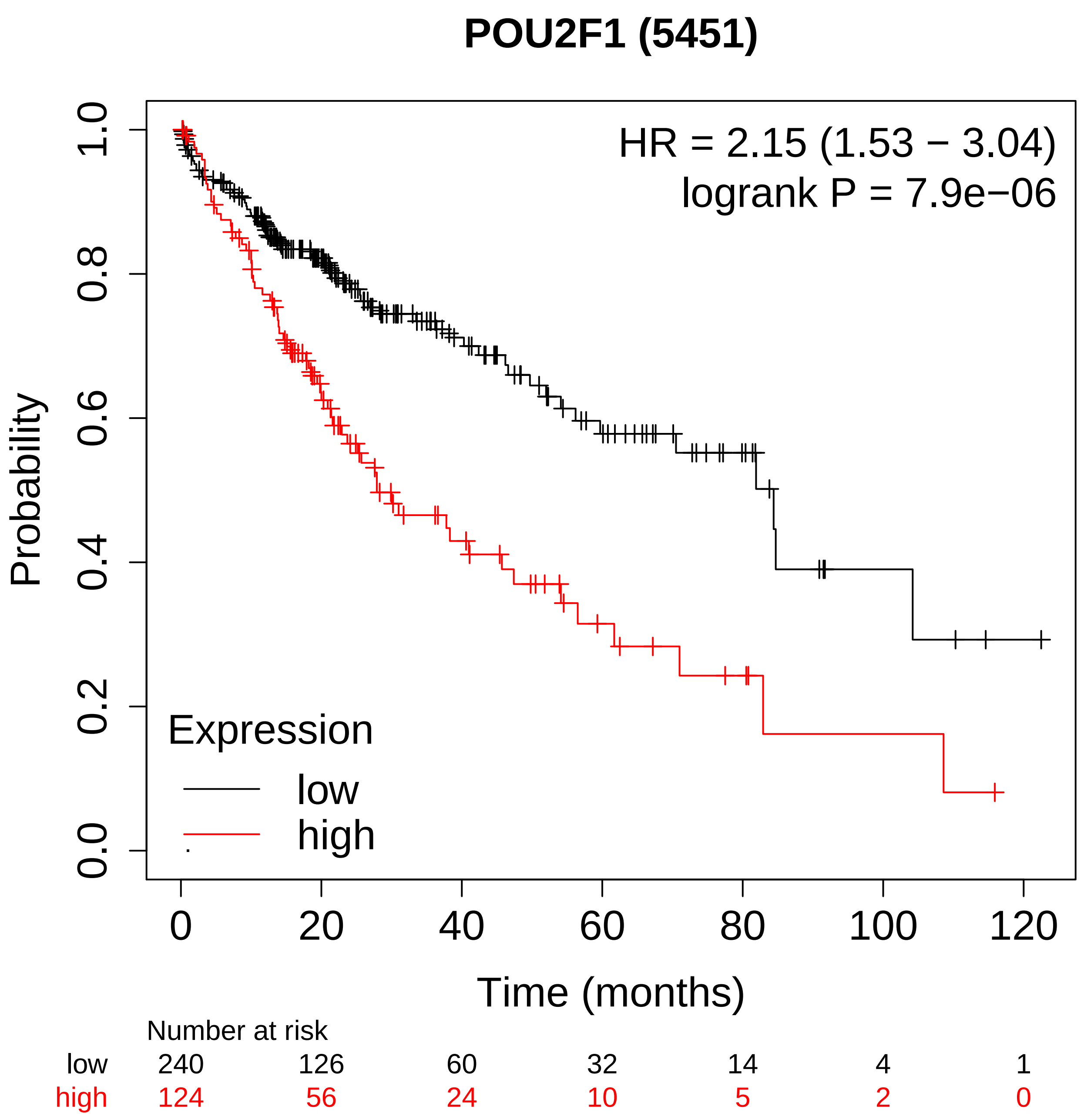
POU2F1 (5451)
HR = 2.15 (1.53 − 3.04)
logrank P = 7.9e−06
Probability
1.0
0.8
0.6
0.4
0.2
0.0
Expression
low
high
0
20
40
60
80
100
120
Time (months)
Number at risk
low 240 126 60 32 14 4 1
high 124 56 24 10 5 2 0

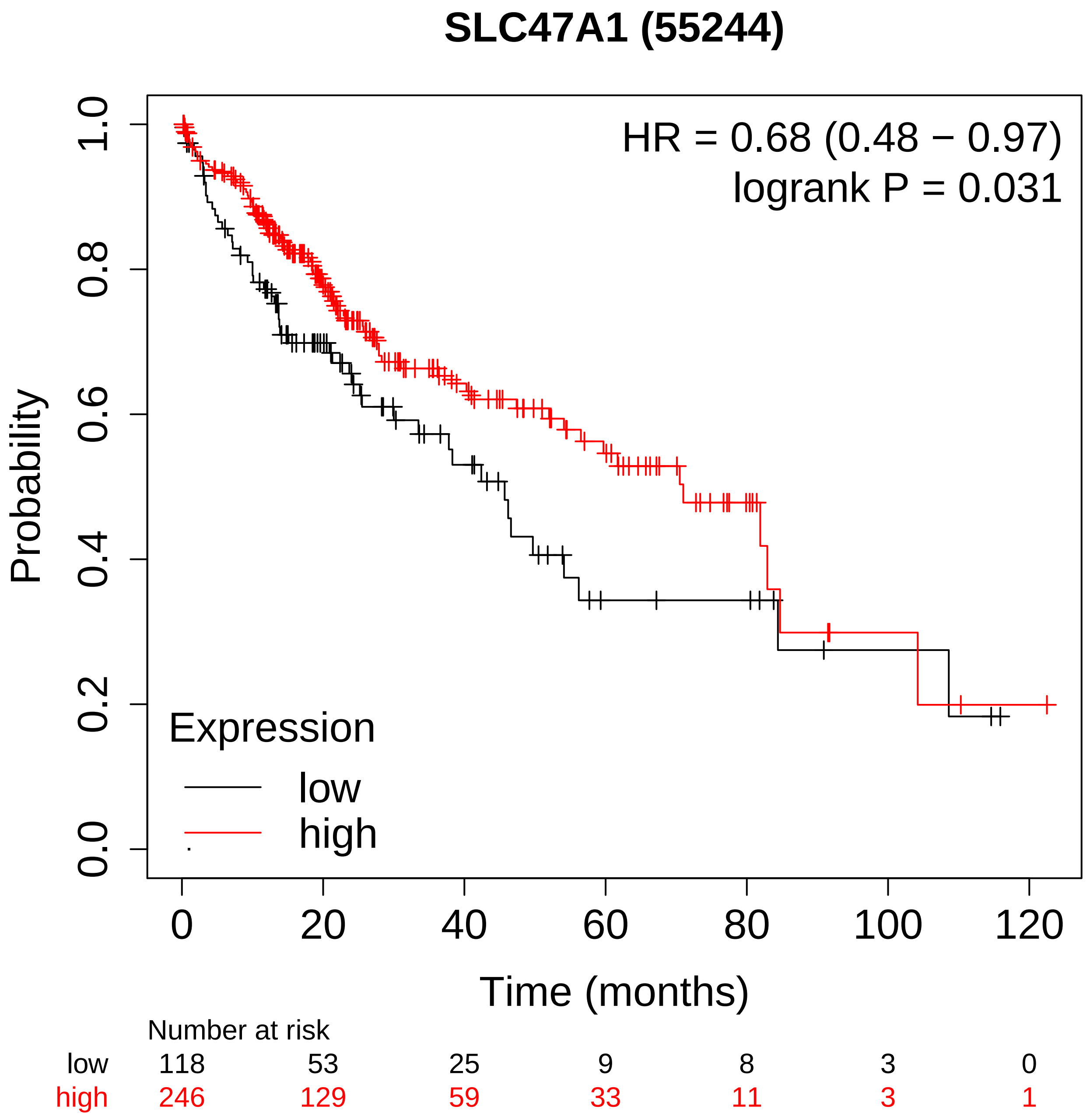
SLC47A1 (55244)
HR = 0.68 (0.48 − 0.97)
logrank P = 0.031
Probability
0.0
0.2
0.4
0.6
0.8
1.0
Time (months)
0
20
40
60
80
100
120
Expression
low
high
Number at risk
low 118 53 25 9 8 3 0
high 246 129 59 33 11 3 1

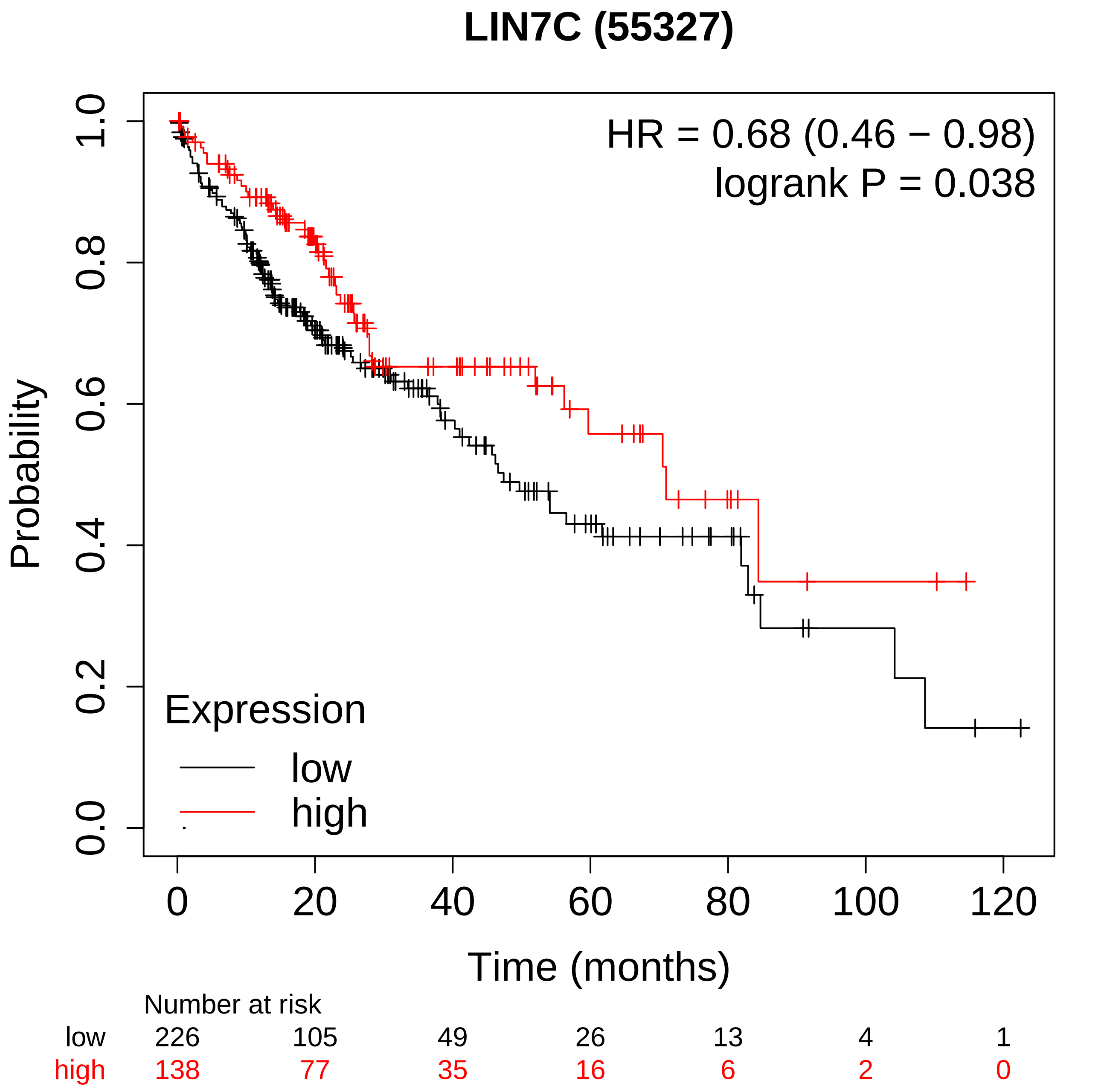
LIN7C (55327)
HR = 0.68 (0.46 − 0.98)
logrank P = 0.038
Probability
0.0
0.2
0.4
0.6
0.8
1.0
Expression
low
high
0
20
40
60
80
100
120
Time (months)
Number at risk
low 226 105 49 26 13 4 1
high 138 77 35 16 6 2 0

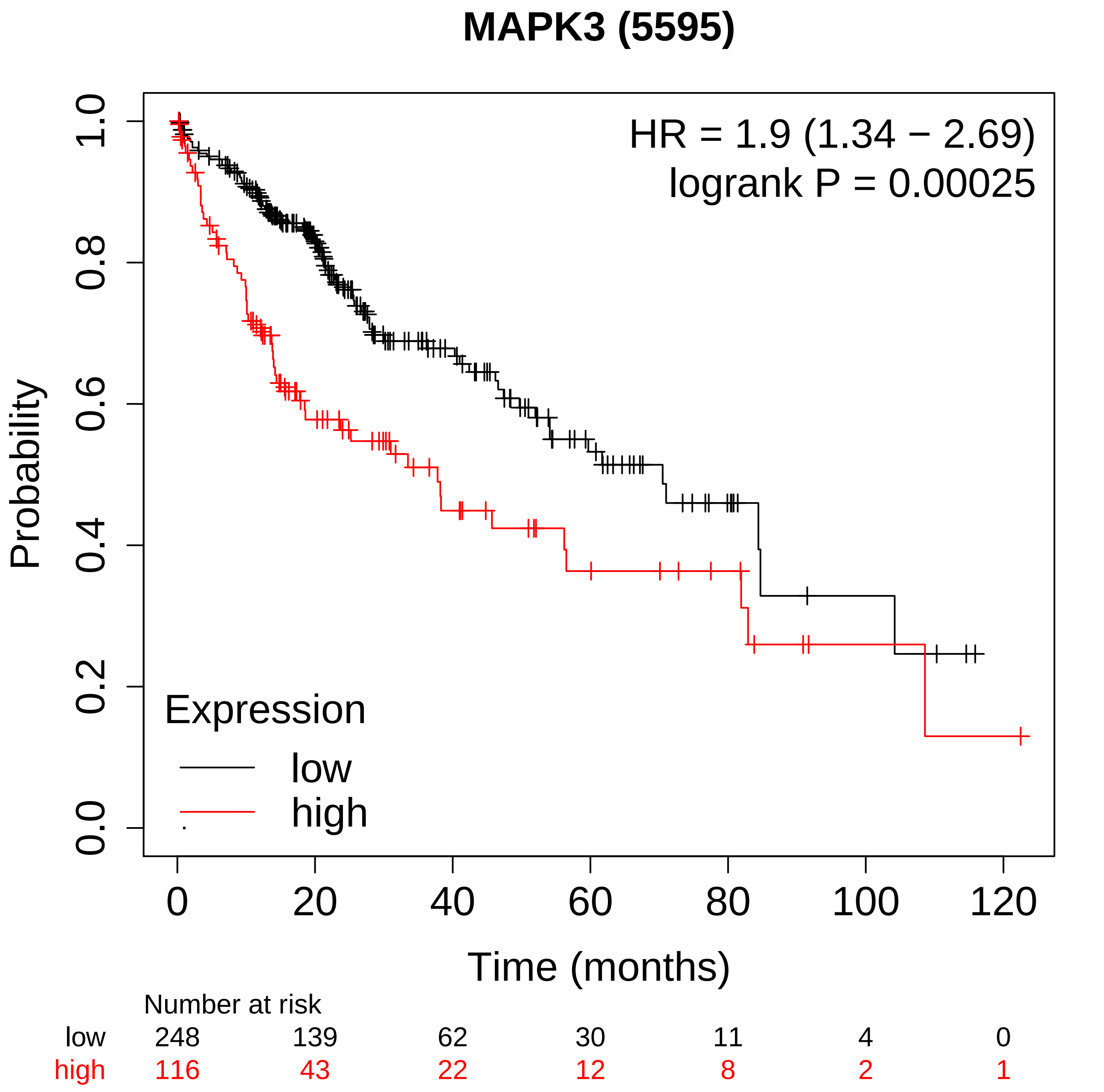
MAPK3 (5595)
HR = 1.9 (1.34 − 2.69)
logrank P = 0.00025
Probability
0.0
0.2
0.4
0.6
0.8
1.0
Expression
low
high
0
20
40
60
80
100
120
Time (months)
Number at risk
low 248 139 62 30 11 4 0
high 116 43 22 12 8 2 1

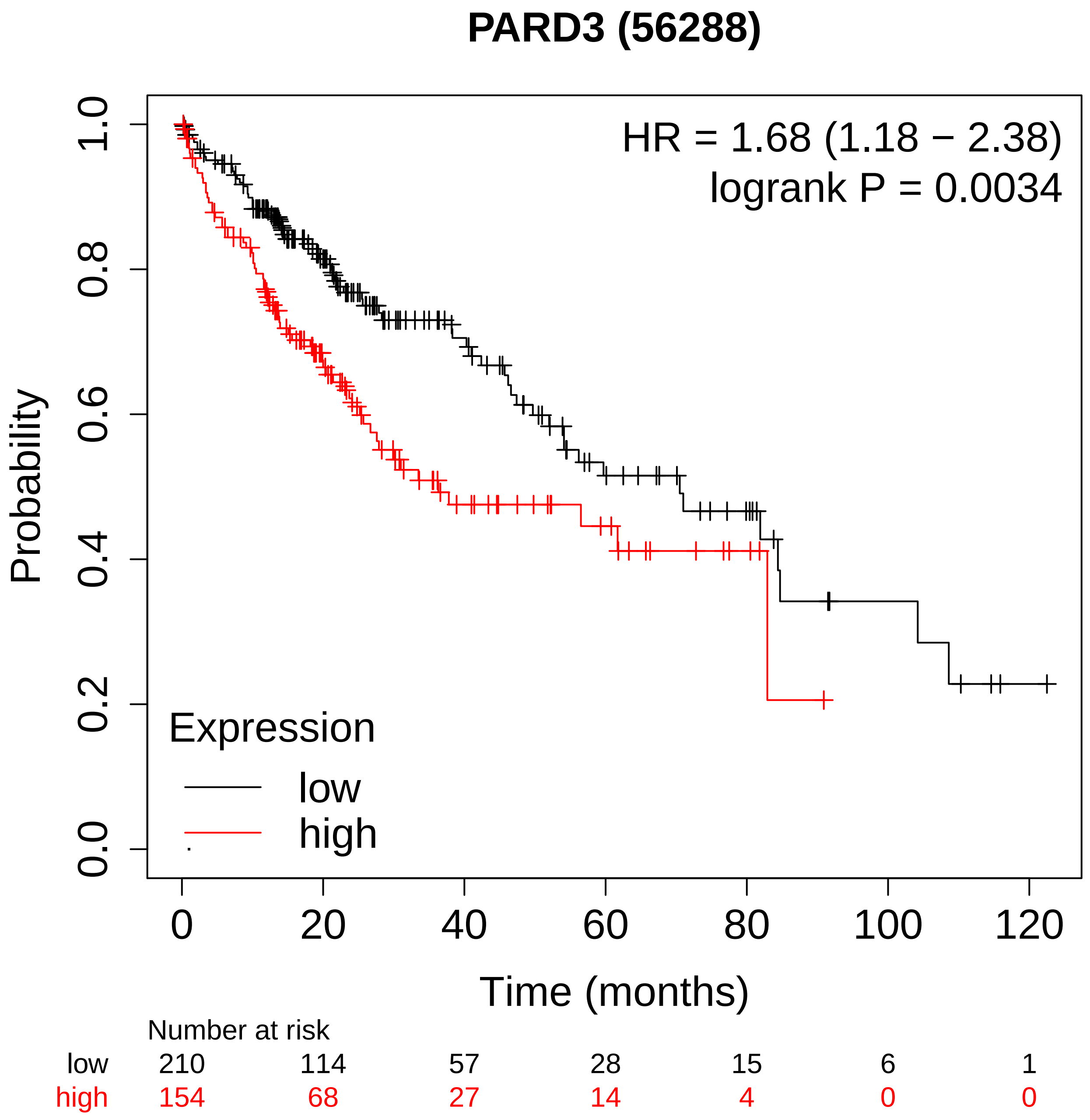
PARD3 (56288)
HR = 1.68 (1.18 − 2.38)
logrank P = 0.0034
Probability
1.0
0.8
0.6
0.4
0.2
0.0
Expression
low
high
0
20
40
60
80
100
120
Time (months)
Number at risk
low 210 114 57 28 15 6 1
high 154 68 27 14 4 0 0

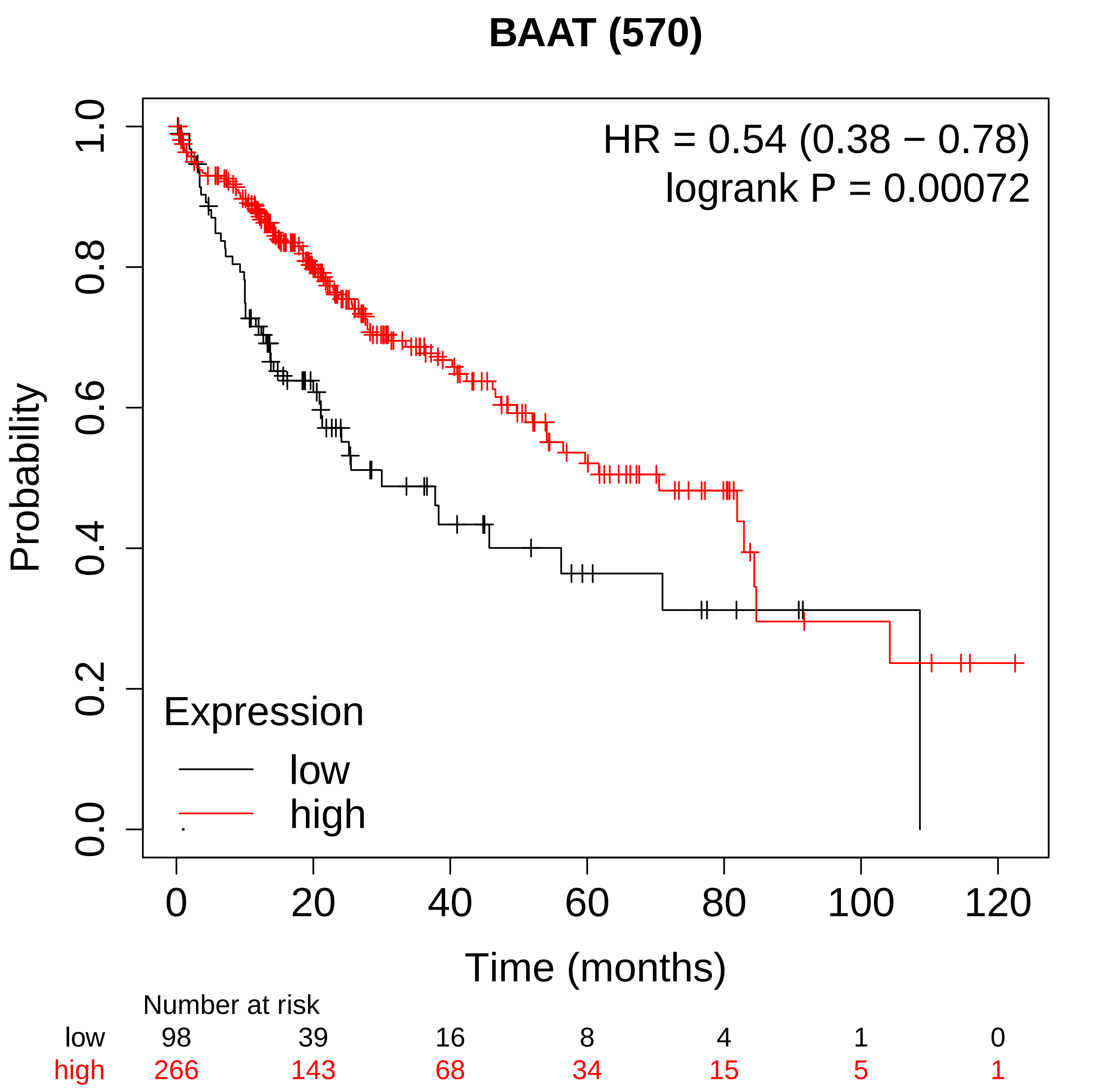
BAAT (570)
HR = 0.54 (0.38 − 0.78)
logrank P = 0.00072
Probability
0.0
0.2
0.4
0.6
0.8
1.0
Expression
low
high
0
20
40
60
80
100
120
Time (months)
Number at risk
low 98 39 16 8 4 1 0
high 266 143 68 34 15 5 1

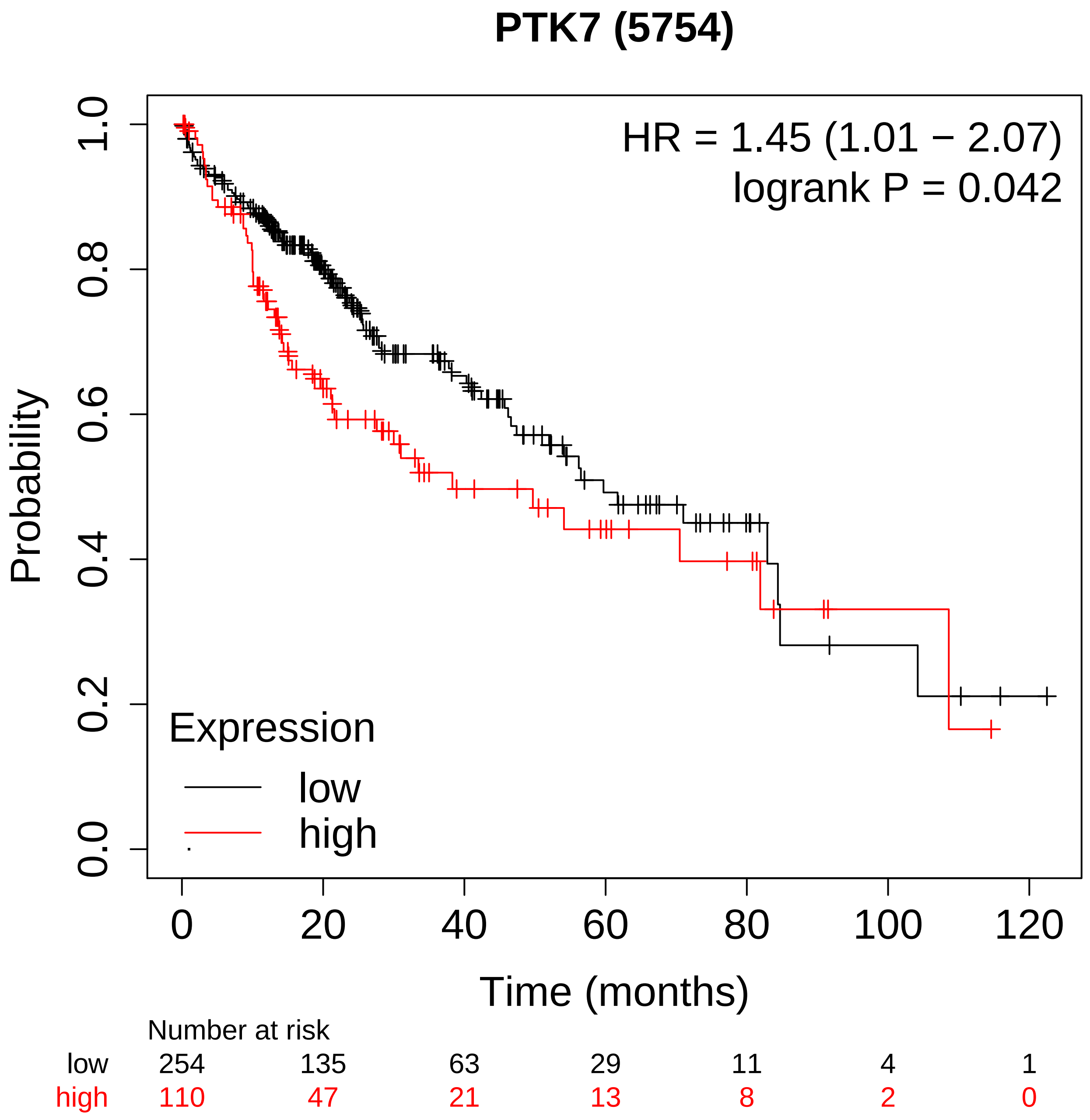
PTK7 (5754)
HR = 1.45 (1.01 − 2.07)
logrank P = 0.042
Probability
0.0
0.2
0.4
0.6
0.8
1.0
Expression
low
high
0
20
40
60
80
100
120
Time (months)
Number at risk
low 254 135 63 29 11 4 1
high 110 47 21 13 8 2 0

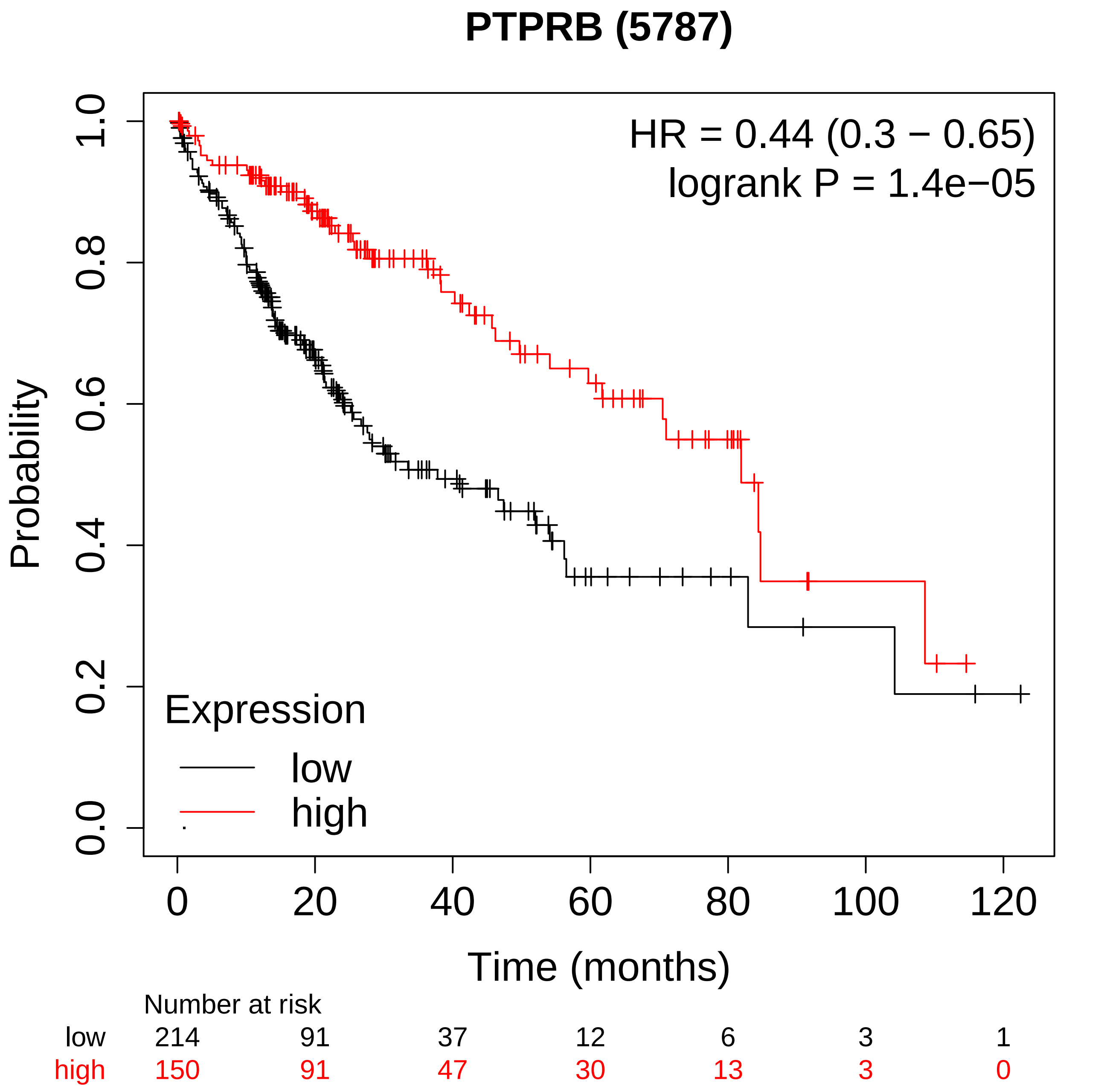
PTPRB (5787)
HR = 0.44 (0.3 − 0.65)
logrank P = 1.4e−05
Probability
0.0
0.2
0.4
0.6
0.8
1.0
Expression
low
high
0
20
40
60
80
100
120
Time (months)
Number at risk
low 214 91 37 12 6 3 1
high 150 91 47 30 13 3 0

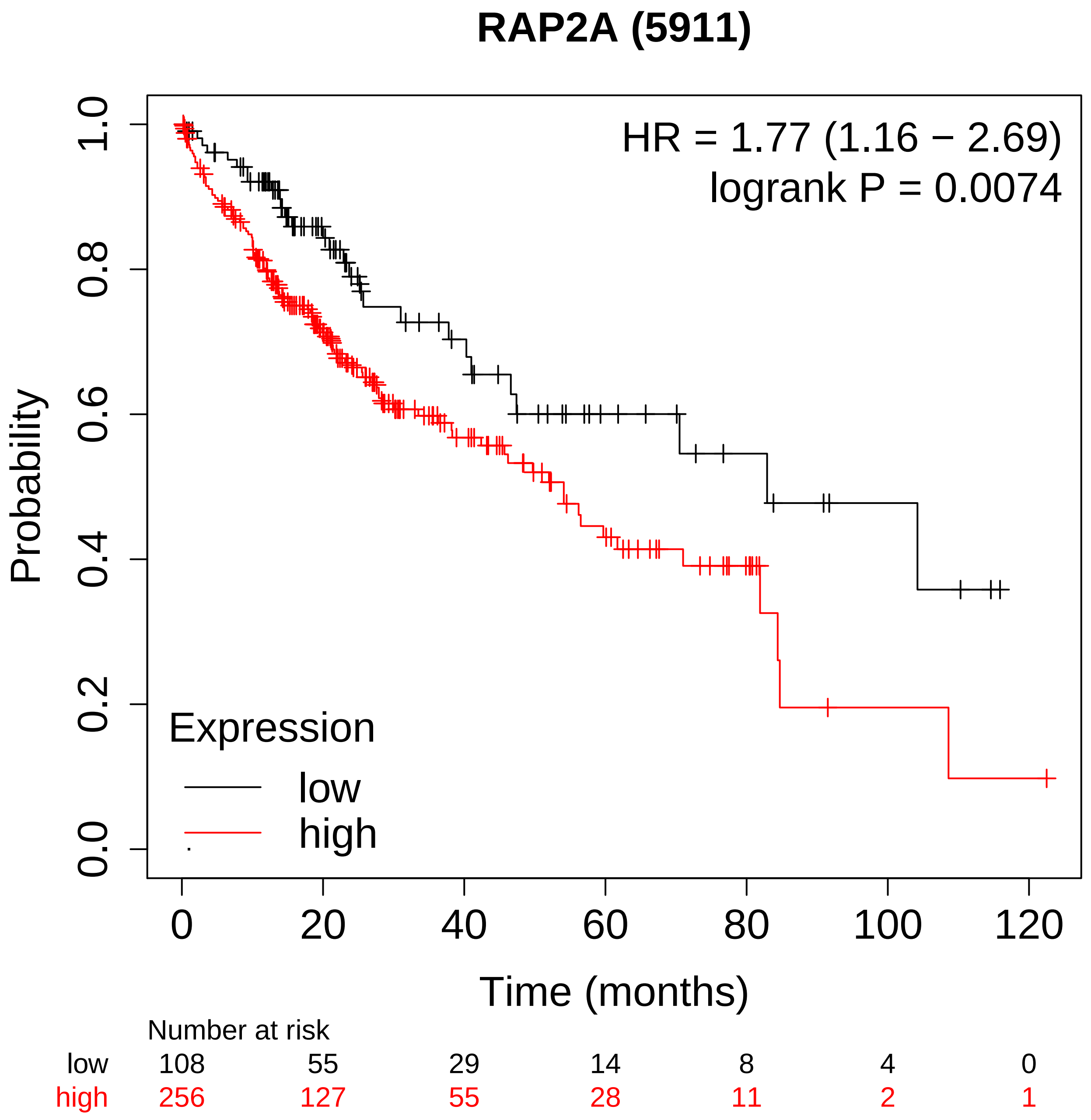

RAP2A (5911)
HR = 1.77 (1.16 − 2.69)
logrank P = 0.0074
Probability
0.0
0.2
0.4
0.6
0.8
1.0
Expression
low
high
0
20
40
60
80
100
120
Time (months)
Number at risk
low 108 55 29 14 8 4 0
high 256 127 55 28 11 2 1

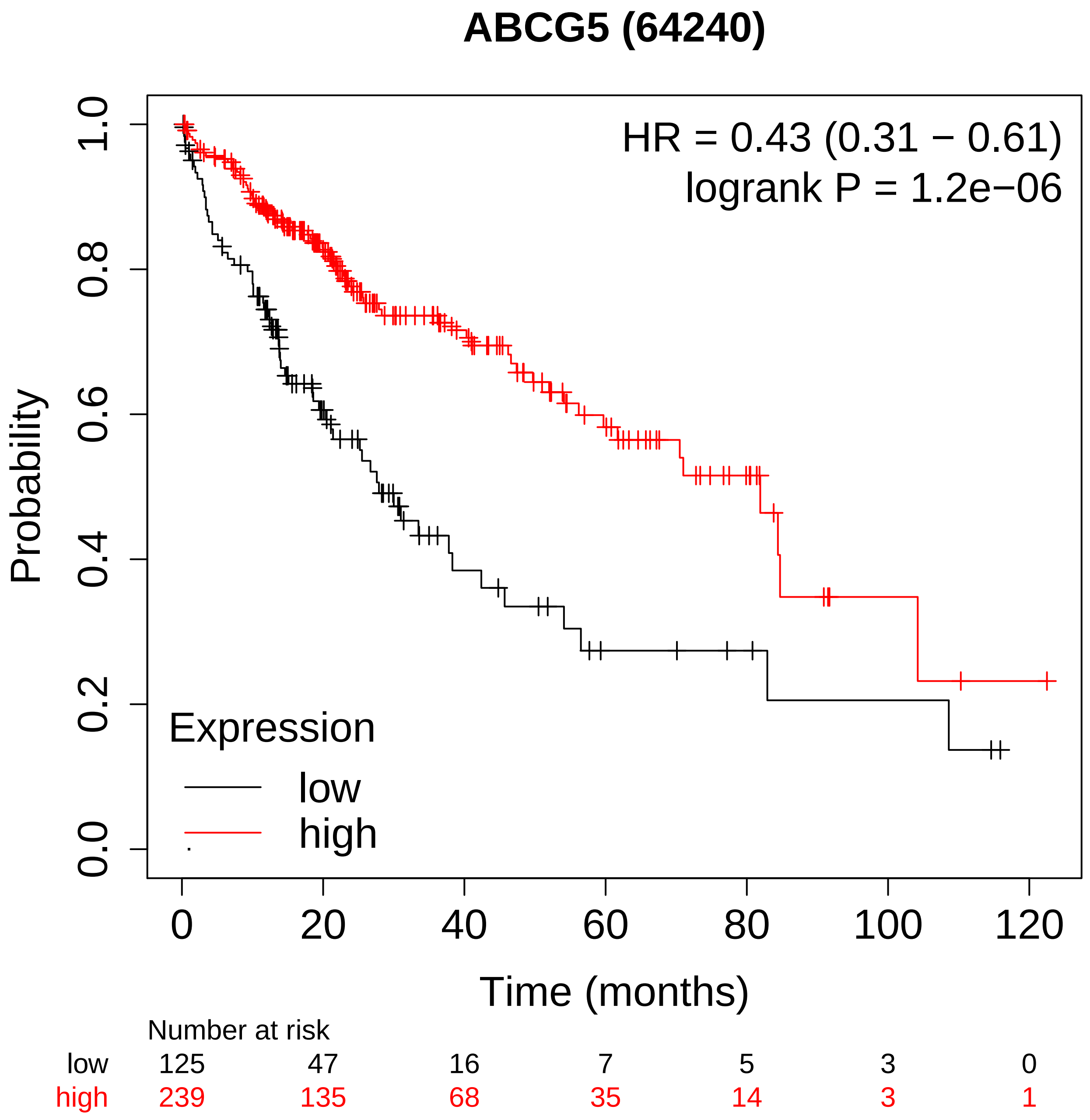
ABCG5 (64240)
HR = 0.43 (0.31 − 0.61)
logrank P = 1.2e−06
Probability
1.0
0.8
0.6
0.4
0.2
0.0
Expression
low
high
0
20
40
60
80
100
120
Time (months)
Number at risk
low 125 47 16 7 5 3 0
high 239 135 68 35 14 3 1

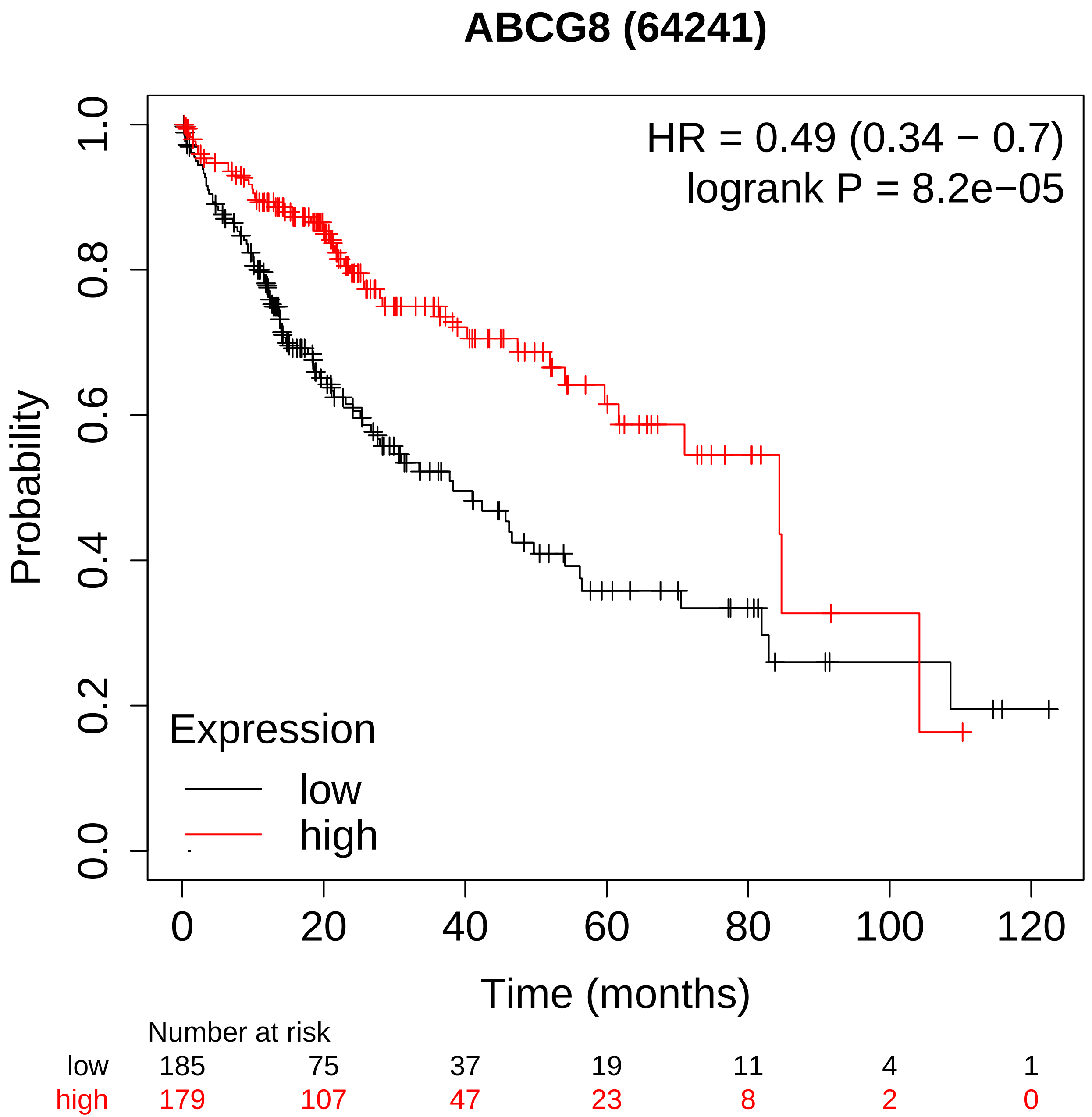
ABCG8 (64241)
HR = 0.49 (0.34 − 0.7)
logrank P = 8.2e−05
Probability
0.0
0.2
0.4
0.6
0.8
1.0
Expression
low
high
0
20
40
60
80
100
120
Time (months)
Number at risk
low 185 75 37 19 11 4 1
high 179 107 47 23 8 2 0

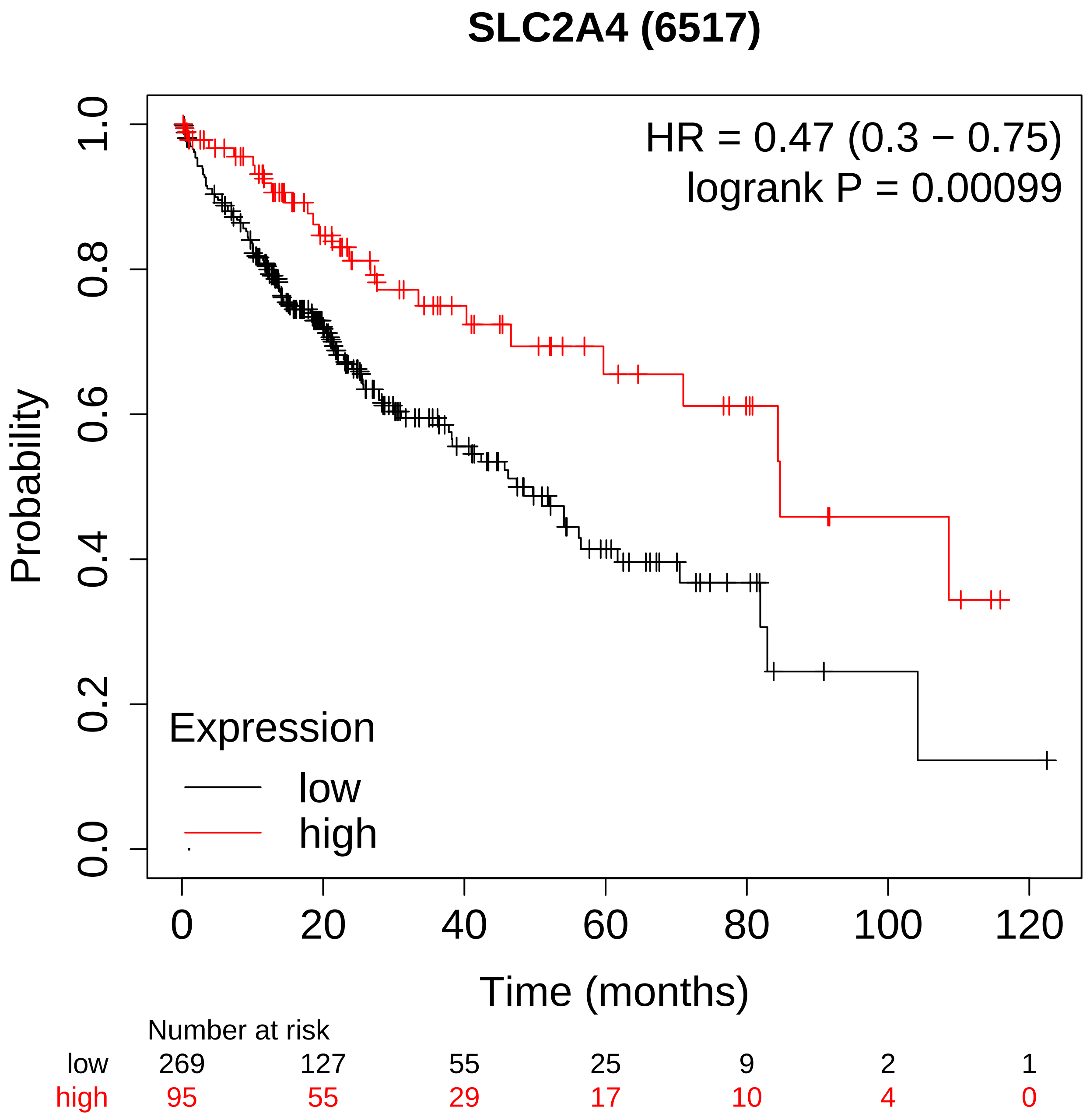

SLC2A4 (6517)
HR = 0.47 (0.3 − 0.75)
logrank P = 0.00099
Probability
1.0
0.8
0.6
0.4
0.2
0.0
Expression
low
high
0
20
40
60
80
100
120
Time (months)
Number at risk
low 269 127 55 25 9 2 1
high 95 55 29 17 10 4 0

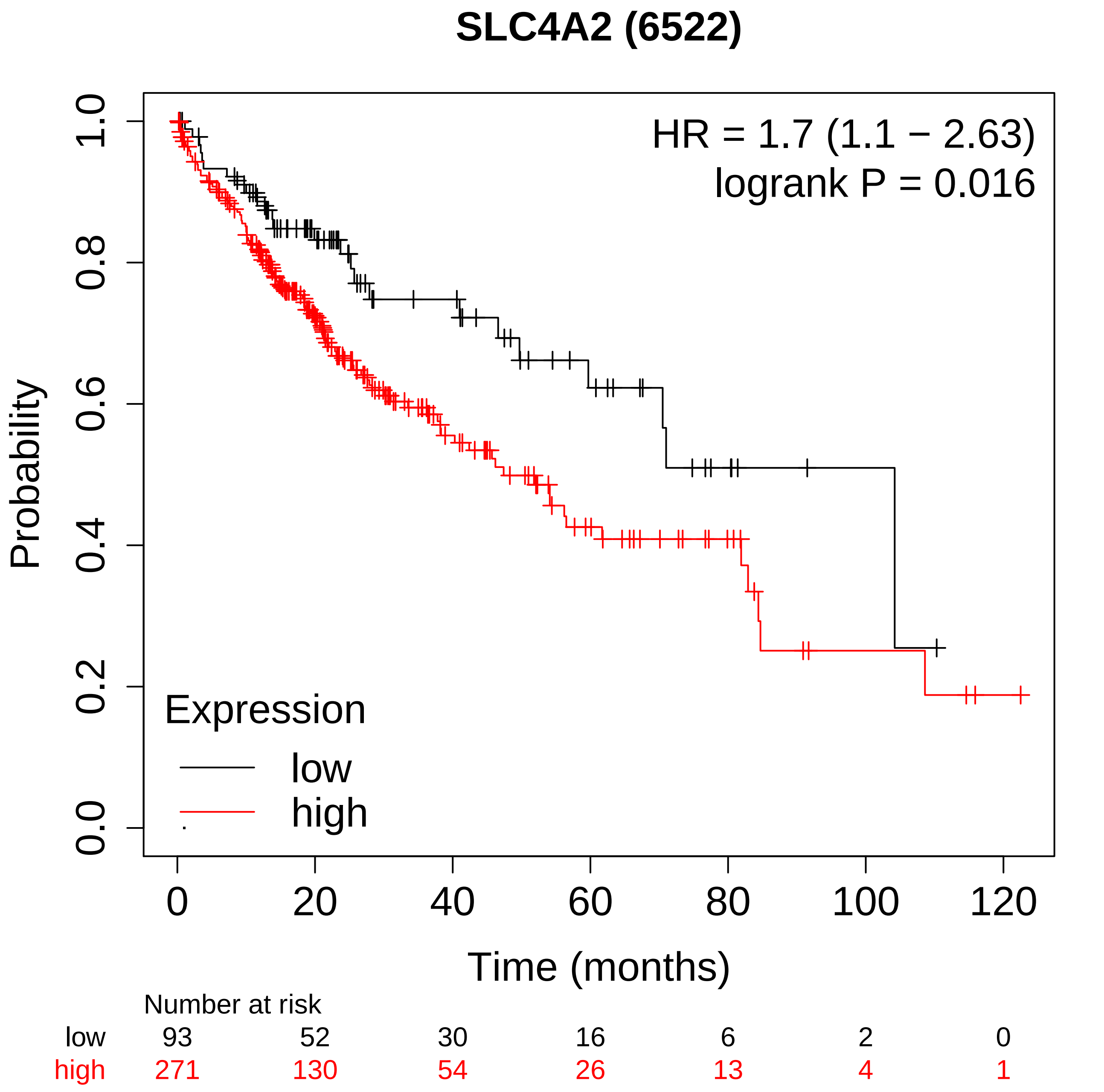
SLC4A2 (6522)
HR = 1.7 (1.1 − 2.63)
logrank P = 0.016
Probability
0.0
0.2
0.4
0.6
0.8
1.0
Expression
low
high
0
20
40
60
80
100
120
Time (months)
Number at risk
low 93 52 30 16 6 2 0
high 271 130 54 26 13 4 1

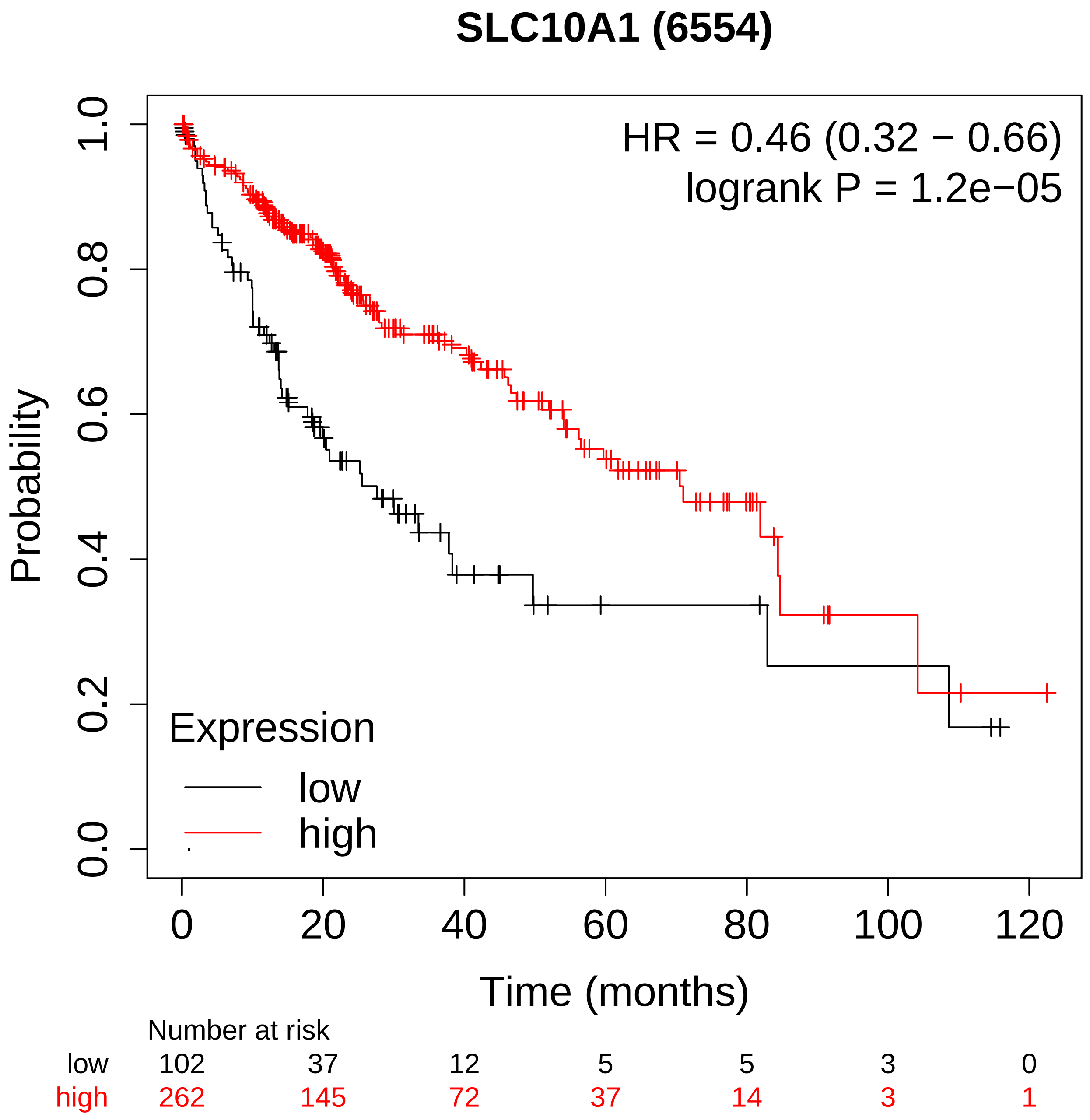
SLC10A1 (6554)
HR = 0.46 (0.32 − 0.66)
logrank P = 1.2e−05
Probability
1.0
0.8
0.6
0.4
0.2
0.0
Expression
low
high
0
20
40
60
80
100
120
Time (months)
Number at risk
low 102 37 12 5 5 3 0
high 262 145 72 37 14 3 1

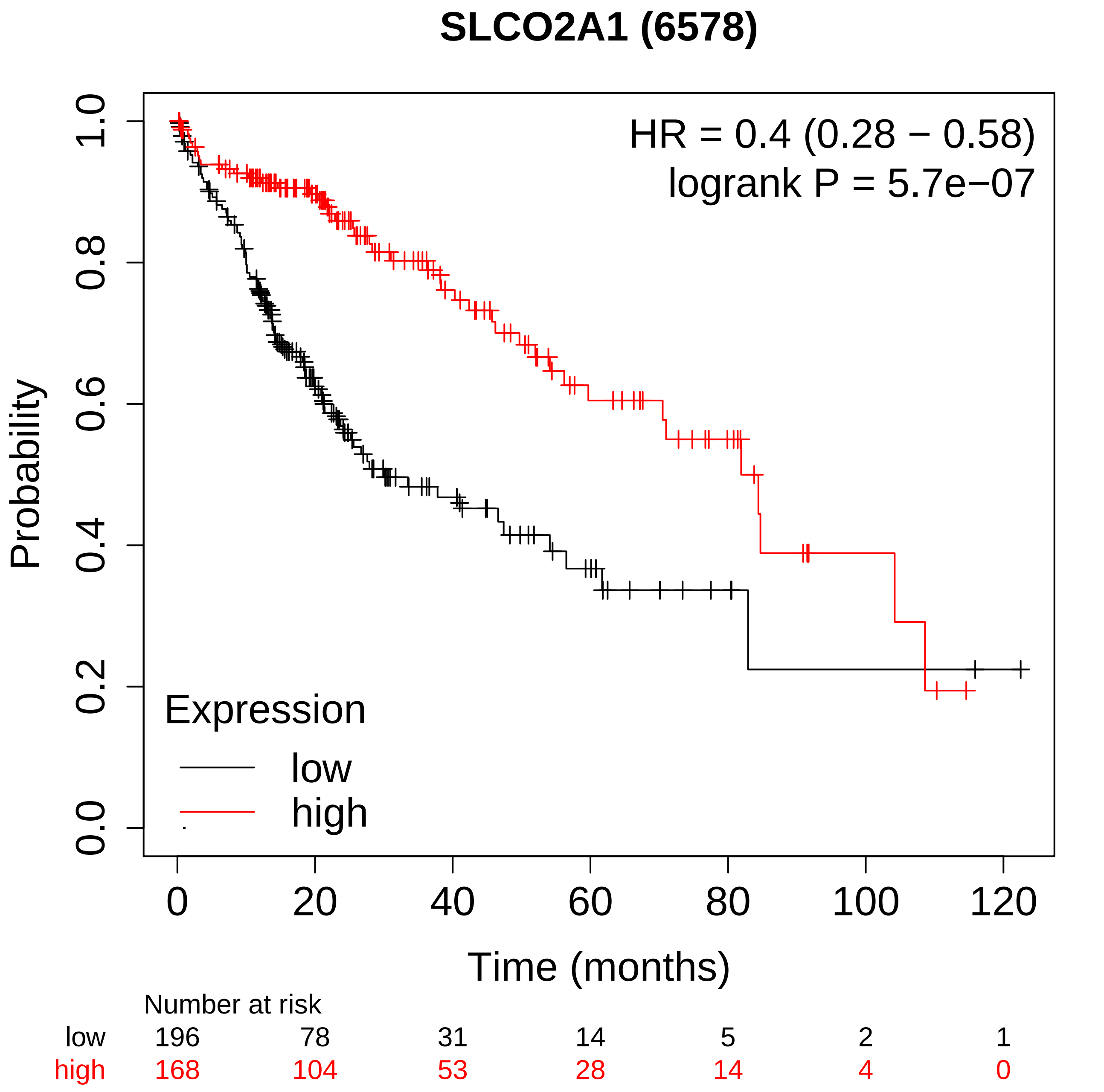
SLCO2A1 (6578)
HR = 0.4 (0.28 − 0.58)
logrank P = 5.7e−07
Probability
1.0
0.8
0.6
0.4
0.2
0.0
Expression
low
high
0
20
40
60
80
100
120
Time (months)
Number at risk
low 196 78 31 14 5 2 1
high 168 104 53 28 14 4 0

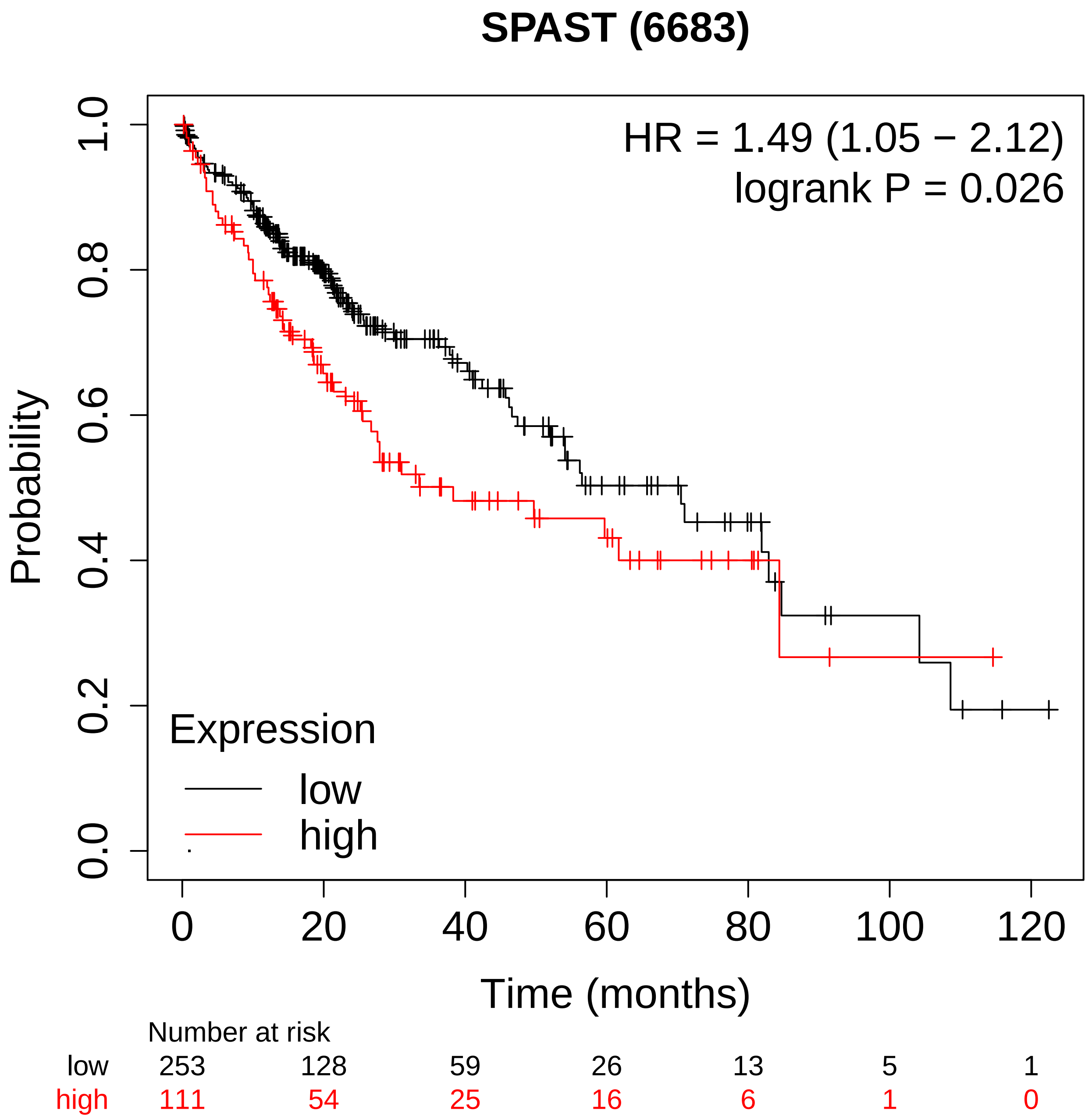
SPAST (6683)
HR = 1.49 (1.05 − 2.12)
logrank P = 0.026
Probability
0.0
0.2
0.4
0.6
0.8
1.0
Expression
low
high
0
20
40
60
80
100
120
Time (months)
Number at risk
low 253 128 59 26 13 5 1
high 111 54 25 16 6 1 0

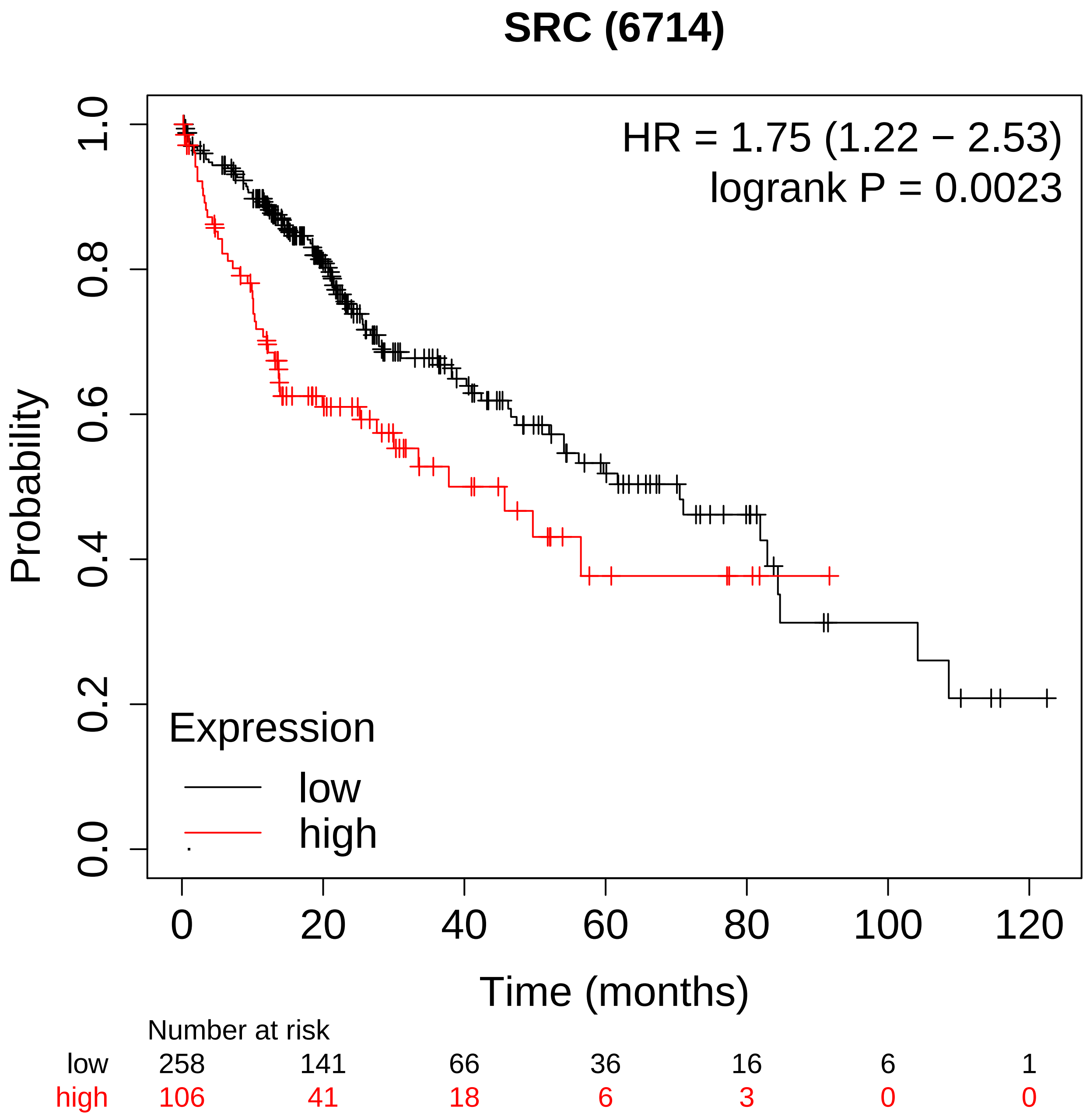

SRC (6714)
HR = 1.75 (1.22 − 2.53)
logrank P = 0.0023
Probability
1.0
0.8
0.6
0.4
0.2
0.0
Expression
low
high
0
20
40
60
80
100
120
Time (months)
Number at risk
low 258 141 66 36 16 6 1
high 106 41 18 6 3 0 0

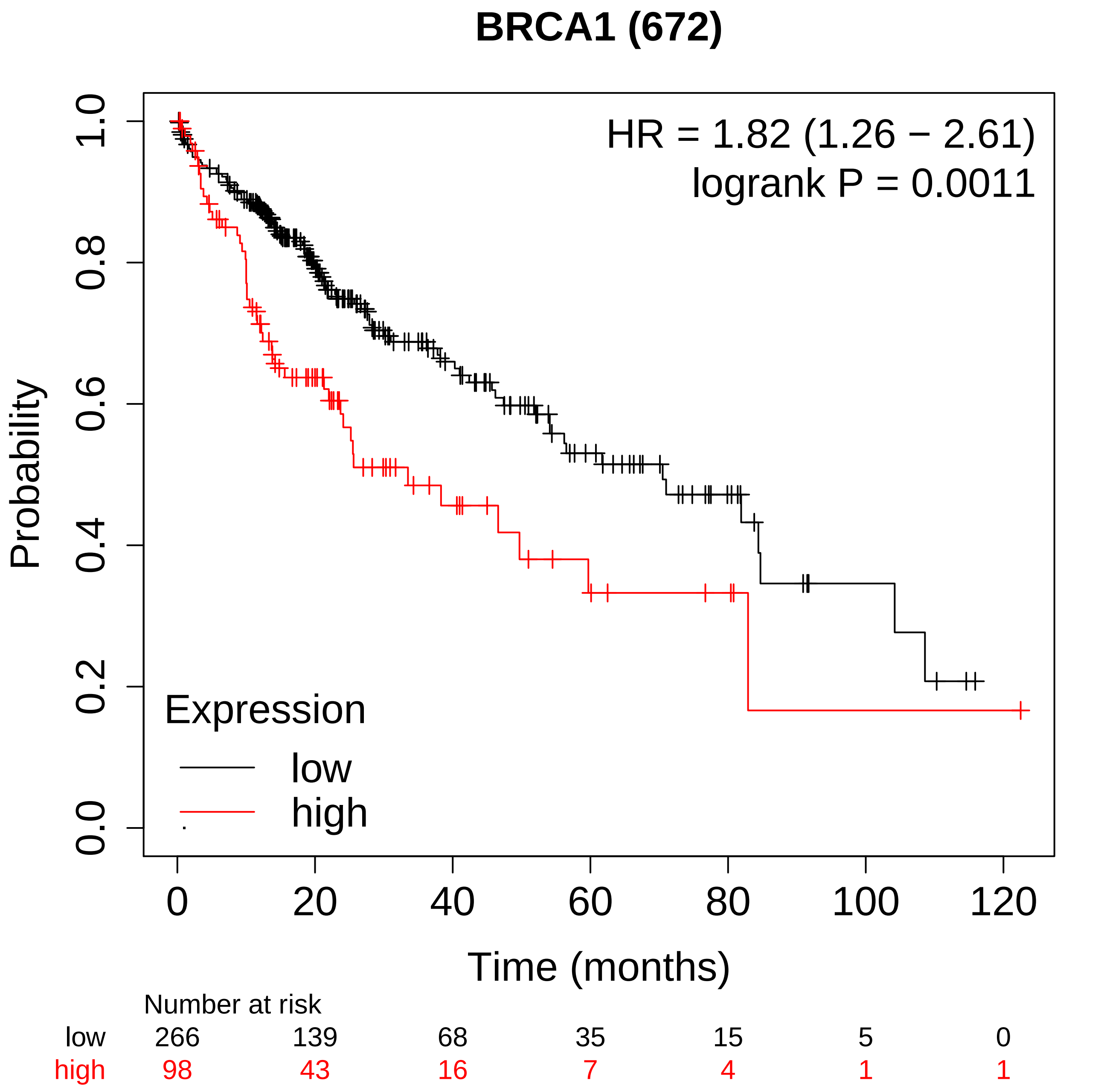
BRCA1 (672)
HR = 1.82 (1.26 − 2.61)
logrank P = 0.0011
Probability
1.0
0.8
0.6
0.4
0.2
0.0
Expression
low
high
0
20
40
60
80
100
120
Time (months)
Number at risk
low 266 139 68 35 15 5 0
high 98 43 16 7 4 1 1

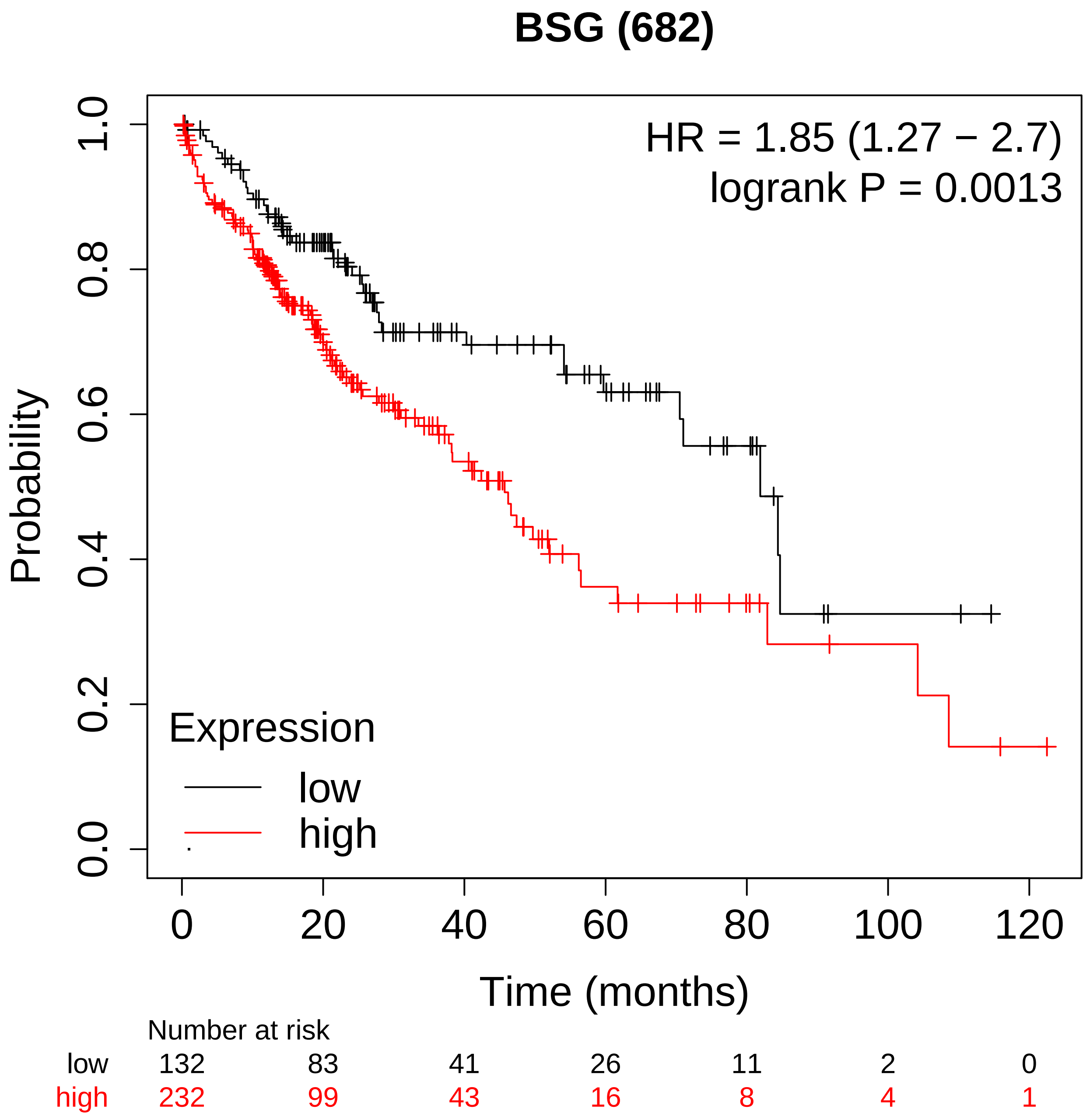

BSG (682)
HR = 1.85 (1.27 − 2.7)
logrank P = 0.0013
Probability
0.0
0.2
0.4
0.6
0.8
1.0
Expression
low
high
0
20
40
60
80
100
120
Time (months)
Number at risk
low 132 83 41 26 11 2 0
high 232 99 43 16 8 4 1

TCF15 (6939)

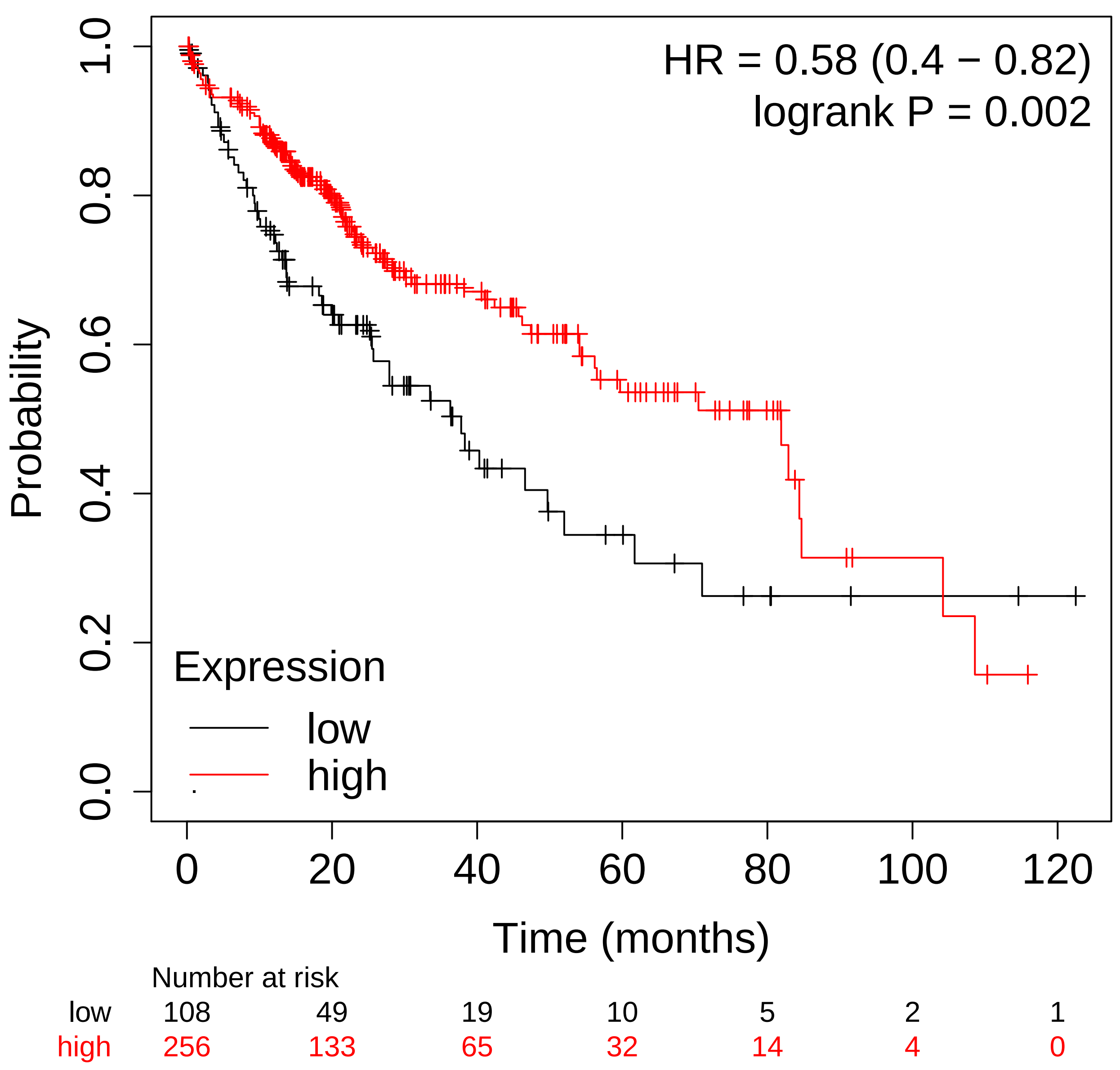
HR = 0.58 (0.4 − 0.82)
logrank P = 0.002
Probability
0.0
0.2
0.4
0.6
0.8
1.0
0
20
40
60
80
100
120
Time (months)
Expression
low
high
Number at risk
low 108 49 19 10 5 2 1
high 256 133 65 32 14 4 0

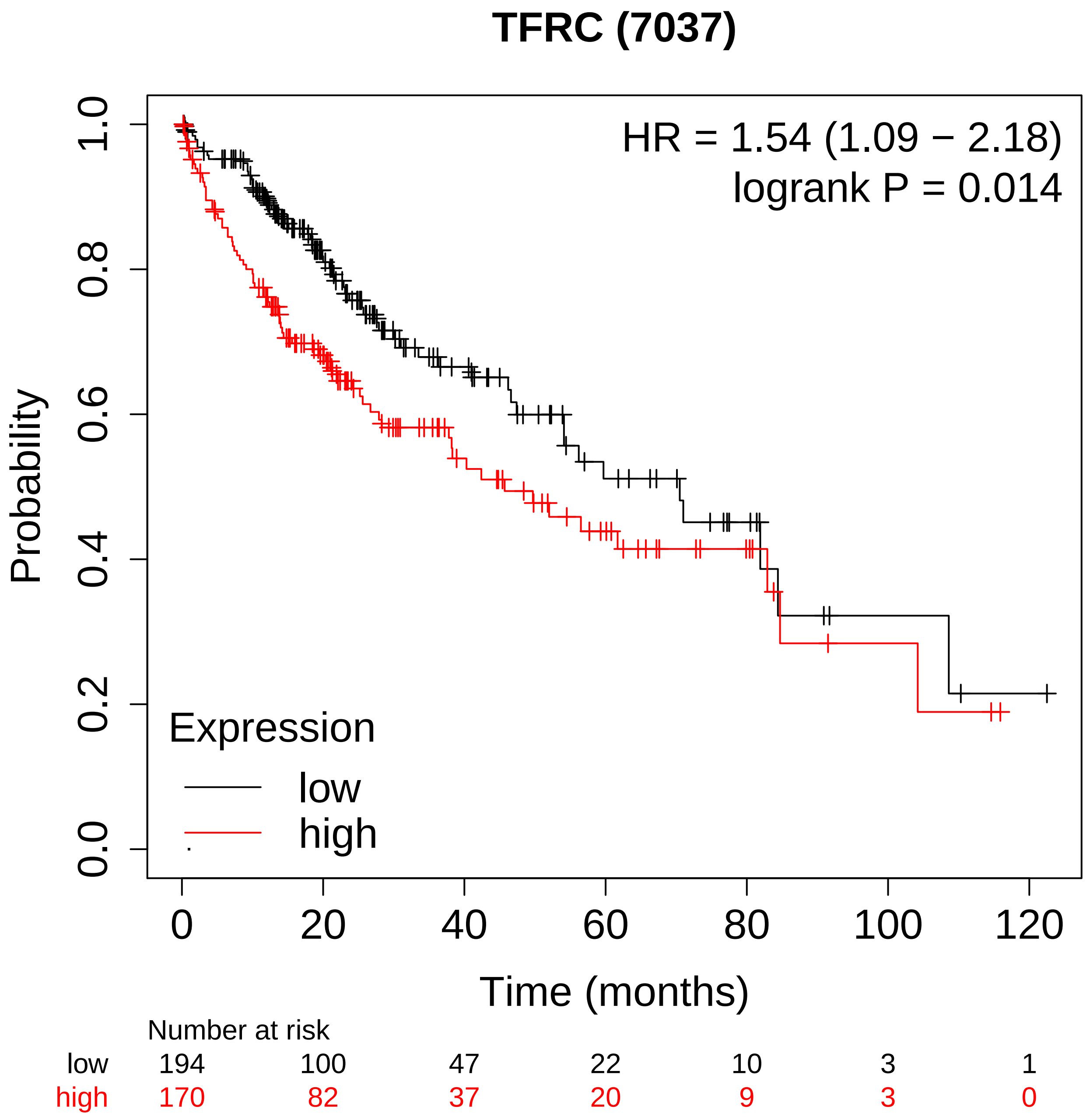
TFRC (7037)
HR = 1.54 (1.09 − 2.18)
logrank P = 0.014
Probability
1.0
0.8
0.6
0.4
0.2
0.0
Expression
low
high
0
20
40
60
80
100
120
Time (months)
Number at risk
low 194 100 47 22 10 3 1
high 170 82 37 20 9 3 0

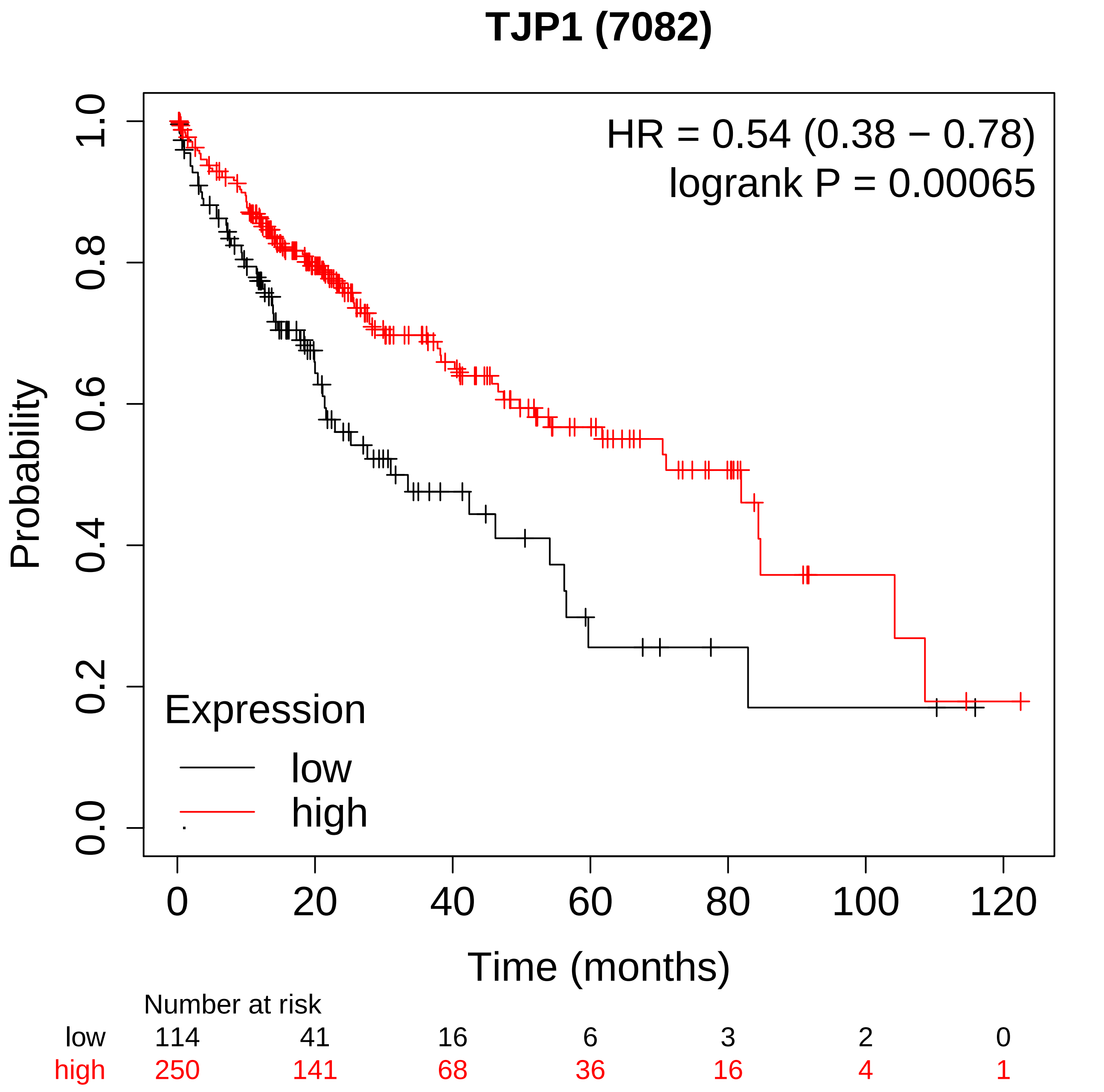

TJP1 (7082)
HR = 0.54 (0.38 − 0.78)
logrank P = 0.00065
Probability
0.0
0.2
0.4
0.6
0.8
1.0
Expression
low
high
0
20
40
60
80
100
120
Time (months)
Number at risk
low 114 41 16 6 3 2 0
high 250 141 68 36 16 4 1

VCL (7414)

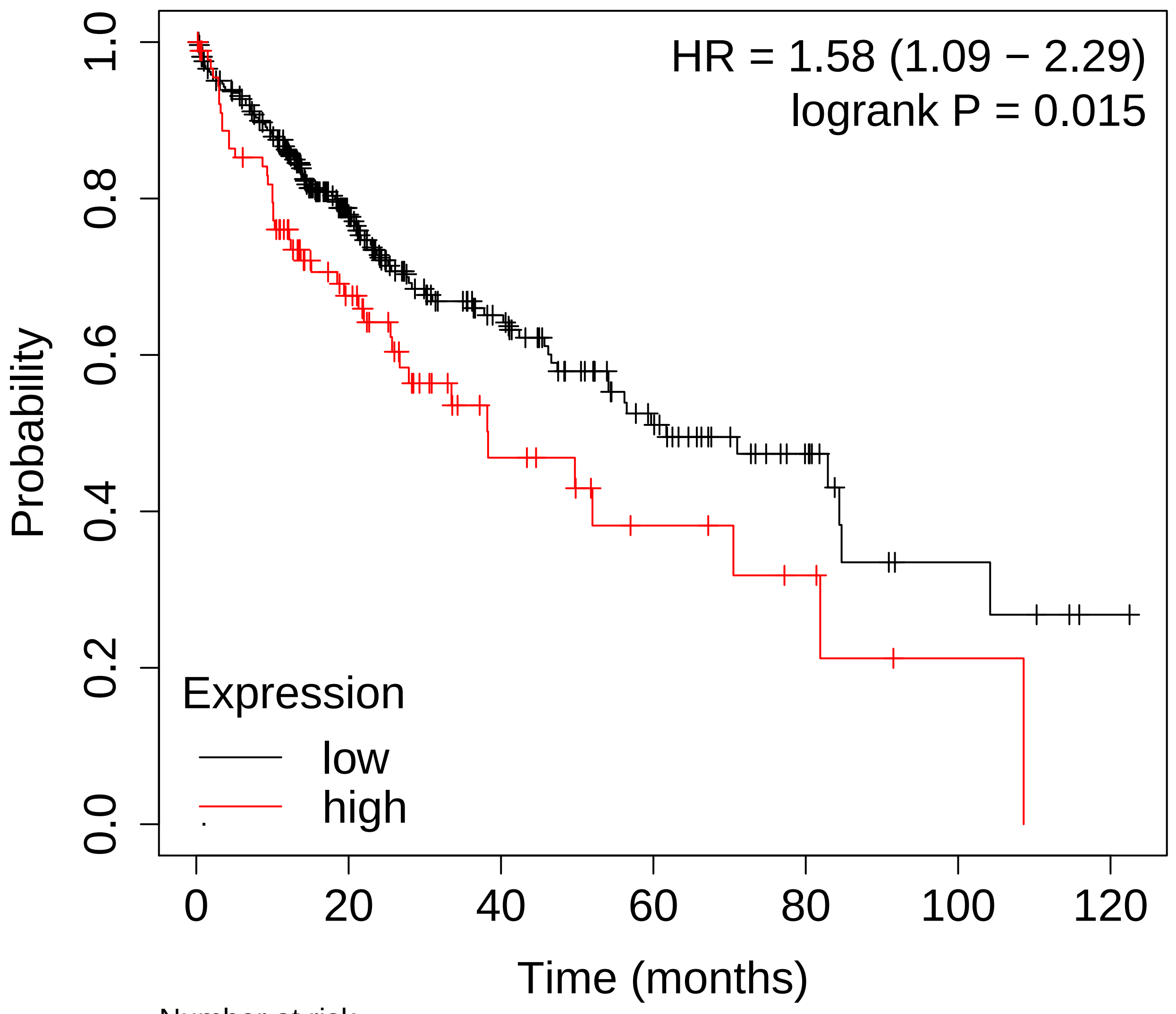

Number at risk

low	272	139	70	35	15	5	1
high	92	43	14	7	4	1	0

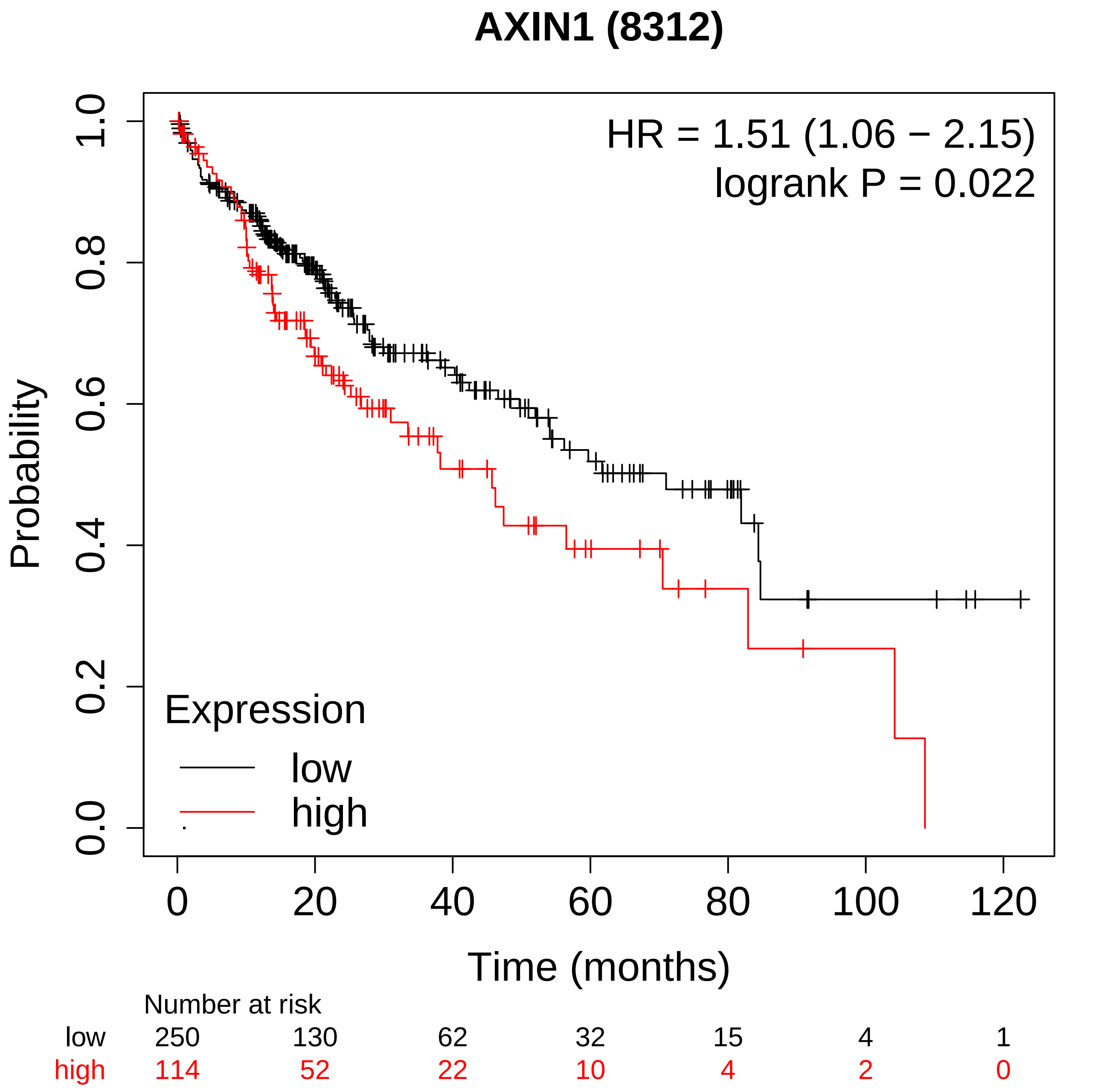
AXIN1 (8312)
HR = 1.51 (1.06 − 2.15)
logrank P = 0.022
Probability
0.0
0.2
0.4
0.6
0.8
1.0
Expression
low
high
0
20
40
60
80
100
120
Time (months)
Number at risk
low 250 130 62 32 15 4 1
high 114 52 22 10 4 2 0

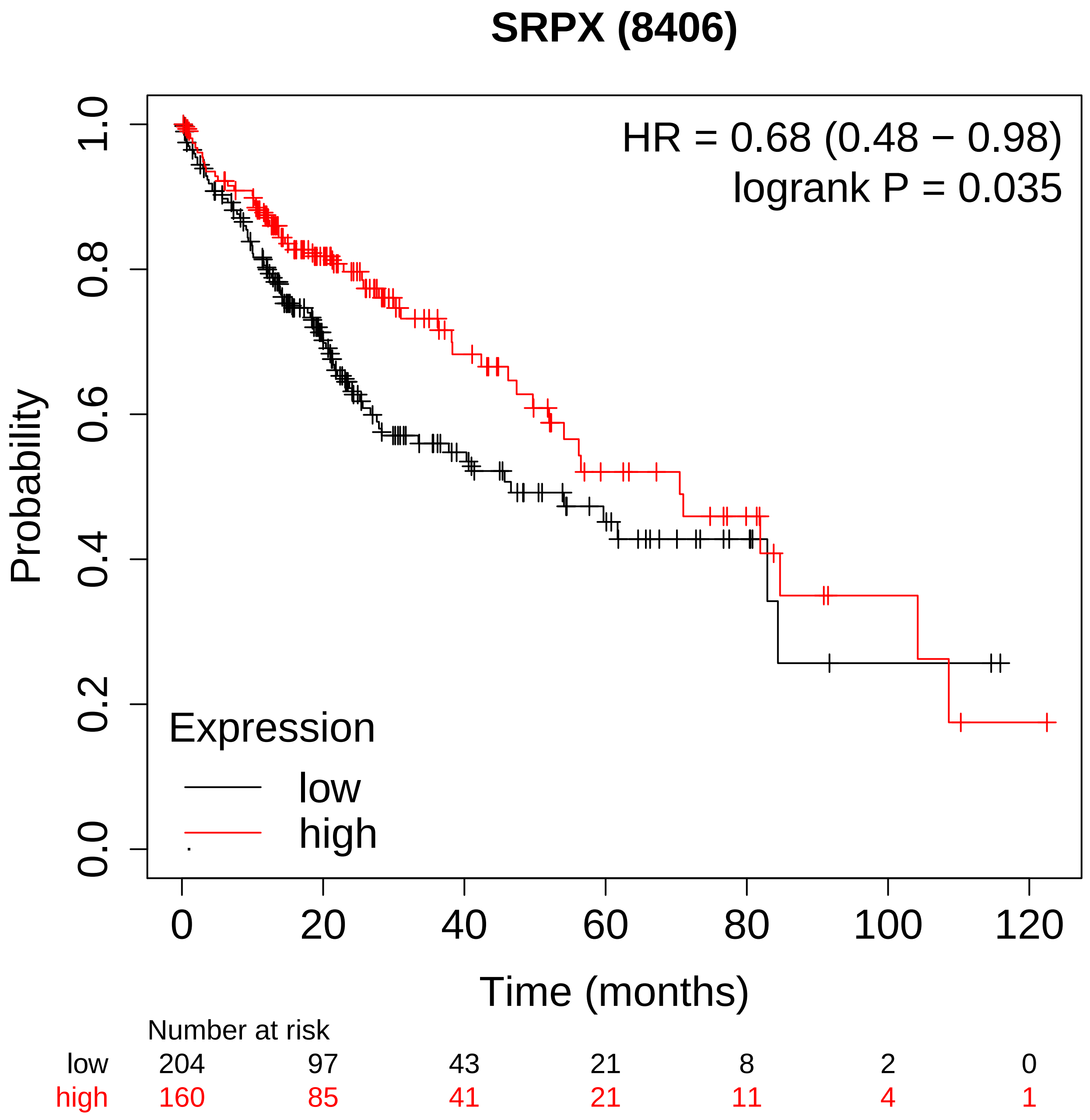
SRPX (8406)
HR = 0.68 (0.48 − 0.98)
logrank P = 0.035
Probability
0.0
0.2
0.4
0.6
0.8
1.0
Expression
low
high
0
20
40
60
80
100
120
Time (months)
Number at risk
low 204 97 43 21 8 2 0
high 160 85 41 21 11 4 1

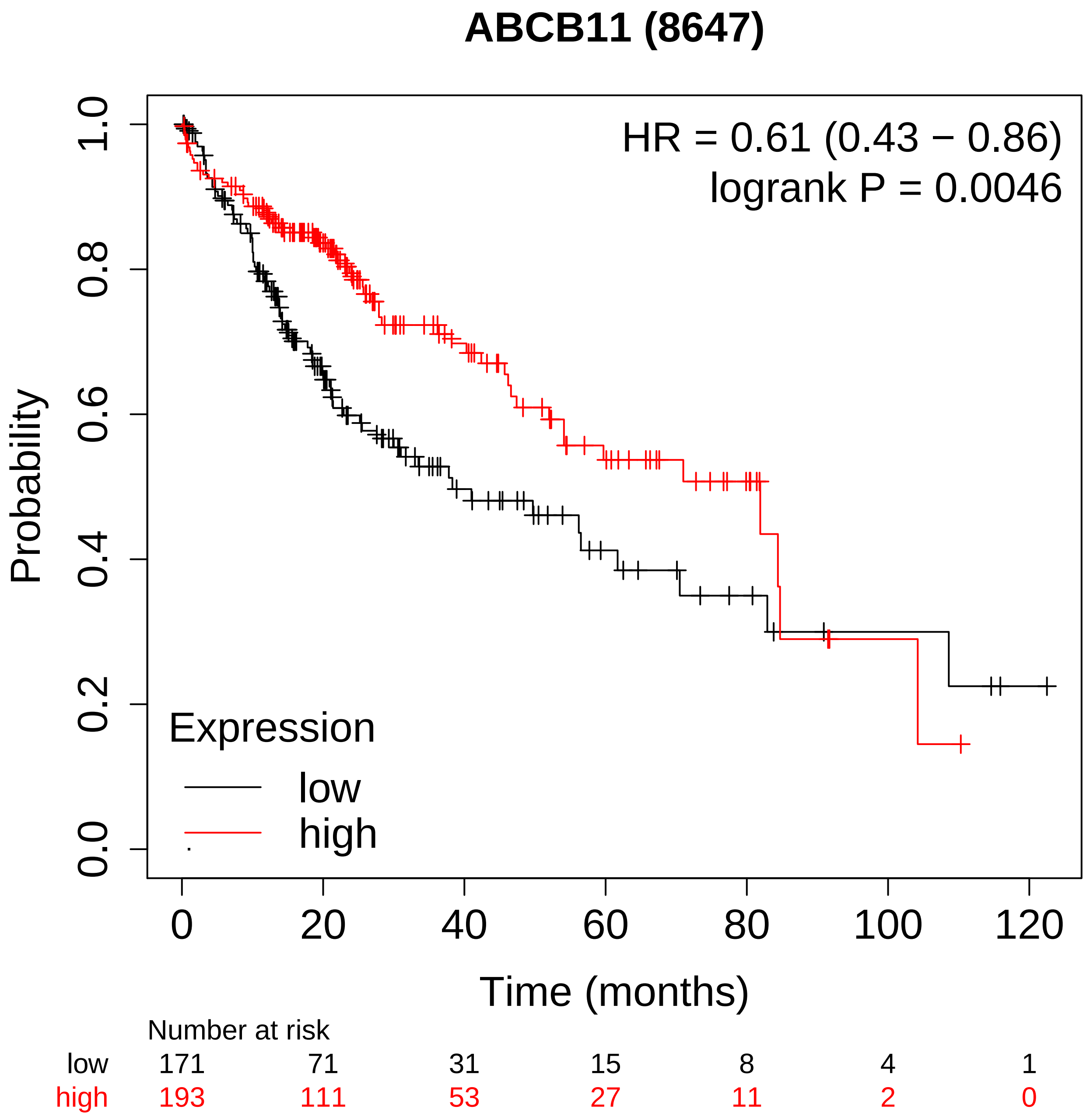
ABCB11 (8647)
HR = 0.61 (0.43 − 0.86)
logrank P = 0.0046
Probability
0.0
0.2
0.4
0.6
0.8
1.0
Expression
low
high
0
20
40
60
80
100
120
Time (months)
Number at risk
low 171 71 31 15 8 4 1
high 193 111 53 27 11 2 0

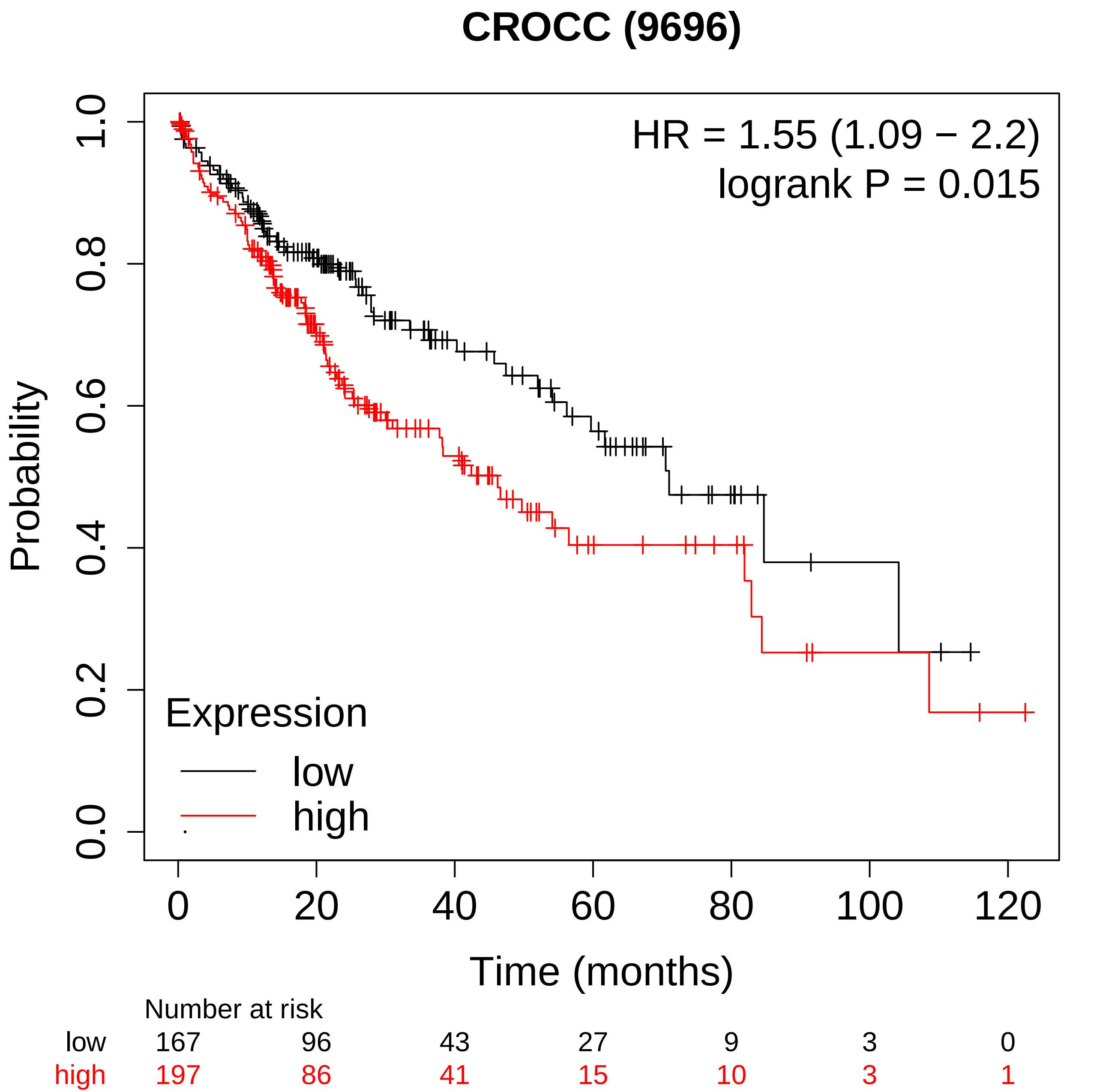
CROCC (9696)
HR = 1.55 (1.09 − 2.2)
logrank P = 0.015
Probability
0.0
0.2
0.4
0.6
0.8
1.0
Expression
low
high
0
20
40
60
80
100
120
Time (months)
Number at risk
low 167 96 43 27 9 3 0
high 197 86 41 15 10 3 1

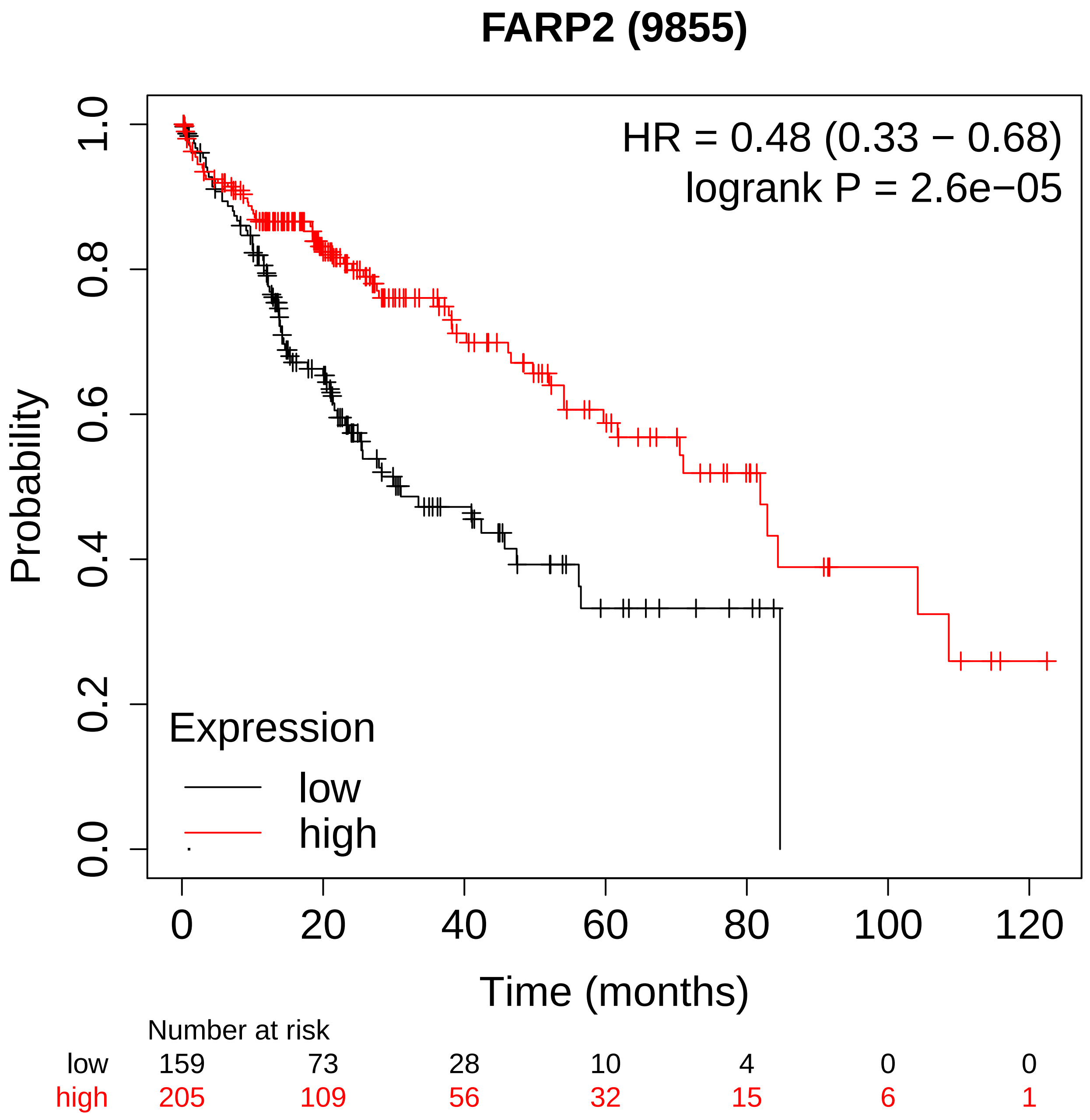

FARP2 (9855)
HR = 0.48 (0.33 − 0.68)
logrank P = 2.6e−05
Probability
1.0
0.8
0.6
0.4
0.2
0.0
Expression
low
high
0
20
40
60
80
100
120
Time (months)
Number at risk
low 159 73 28 10 4 0 0
high 205 109 56 32 15 6 1

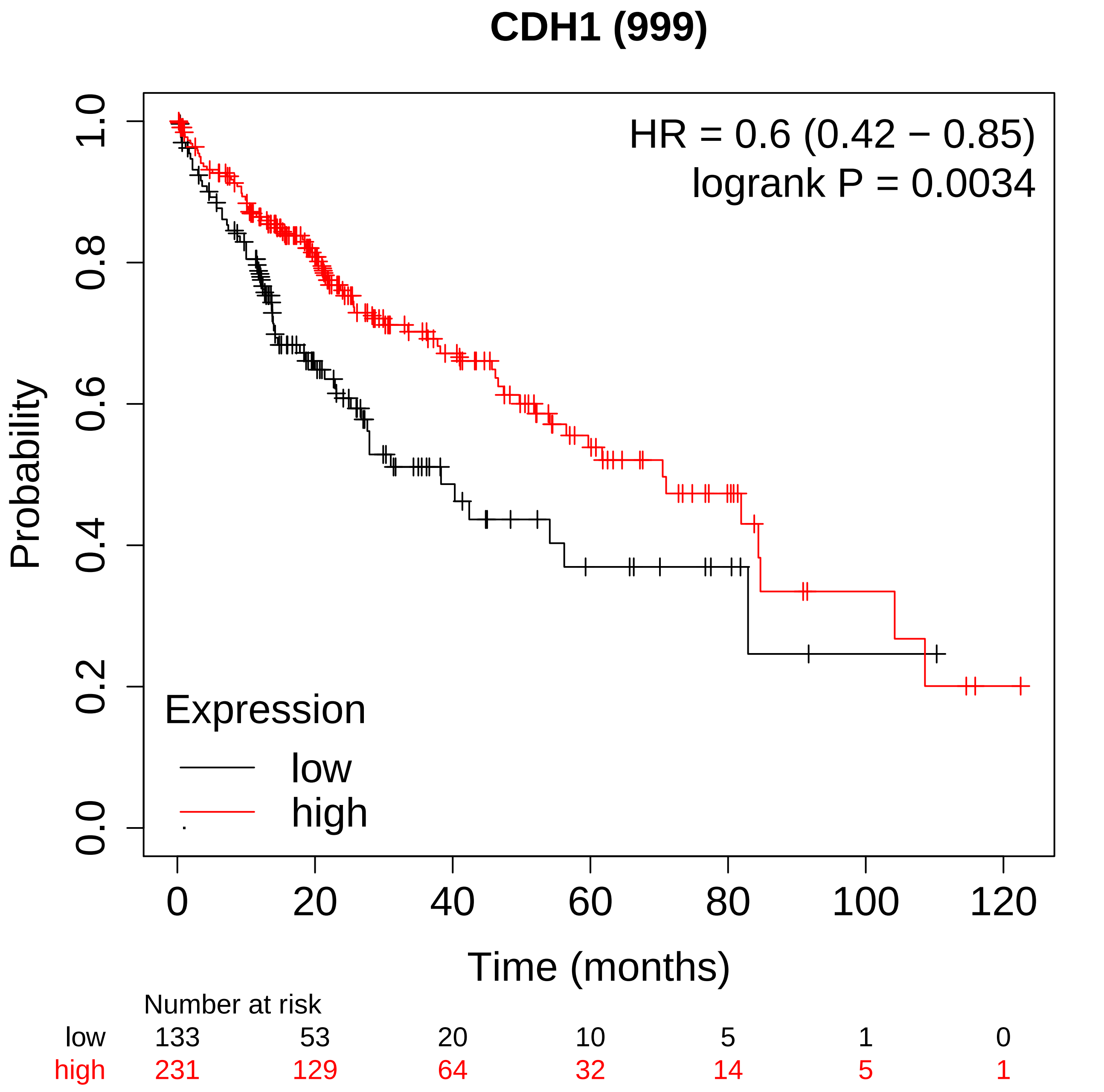

CDH1 (999)
HR = 0.6 (0.42 − 0.85)
logrank P = 0.0034
Probability
0.0
0.2
0.4
0.6
0.8
1.0
Expression
low
high
0
20
40
60
80
100
120
Time (months)
Number at risk
low 133 53 20 10 5 1 0
high 231 129 64 32 14 5 1
