# Supplementary material for: Expression, Prognostic Value, and Functional Mechanism of Polarity-Related Genes in Hepatocellular Carcinoma
Source: Int J Mol Sci. 2022 Oct 24;23(21):12784. doi: 10.3390/ijms232112784 (PMC9655479; doi:10.3390/ijms232112784)
Supplement: Supplementary file 1 [file ijms-23-12784-s001.zip › Supplementary Figure_S4_features of elastic net.pdf]

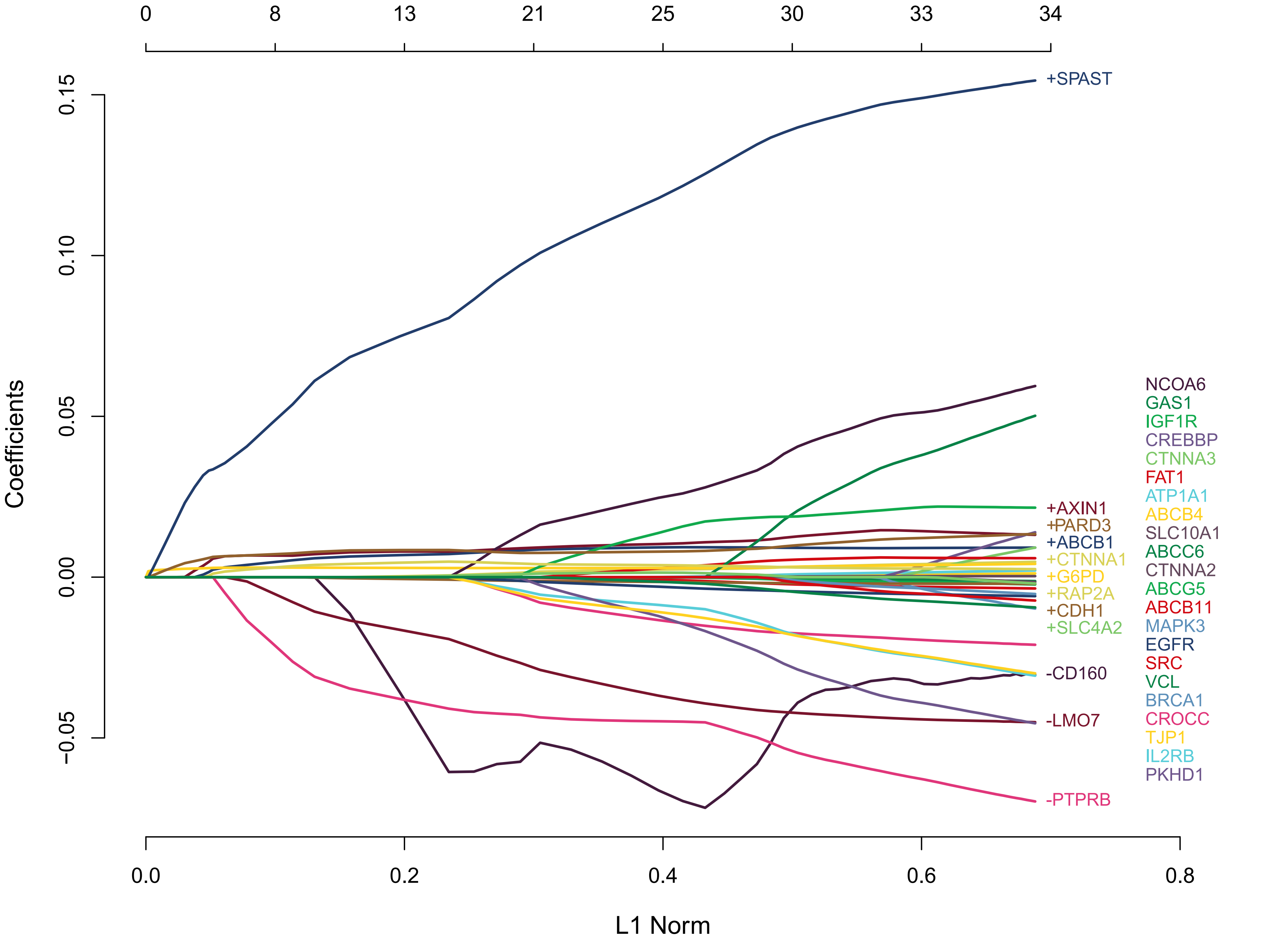

0
8
13
21
25
30
33
34
Coefficients
0.15
0.10
0.05
0.00
−0.05
+SPAST
+AXIN1
+PARD3
+ABCB1
+CTNNA1
+G6PD
+RAP2A
+CDH1
+SLC4A2
-CD160
-LMO7
-PTPRB
NCOA6
GAS1
IGF1R
CREBBP
CTNNA3
FAT1
ATP1A1
ABCB4
SLC10A1
ABCC6
CTNNA2
ABCG5
ABCB11
MAPK3
EGFR
SRC
VCL
BRCA1
CROCC
TJP1
IL2RB
PKHD1
0.0
0.2
0.4
0.6
0.8
L1 Norm
