# Supplementary figures and images for: Expression, Prognostic Value, and Functional Mechanism of Polarity-Related Genes in Hepatocellular Carcinoma
Source: Int J Mol Sci. 2022 Oct 24;23(21):12784. doi: 10.3390/ijms232112784 (PMC9655479; doi:10.3390/ijms232112784)

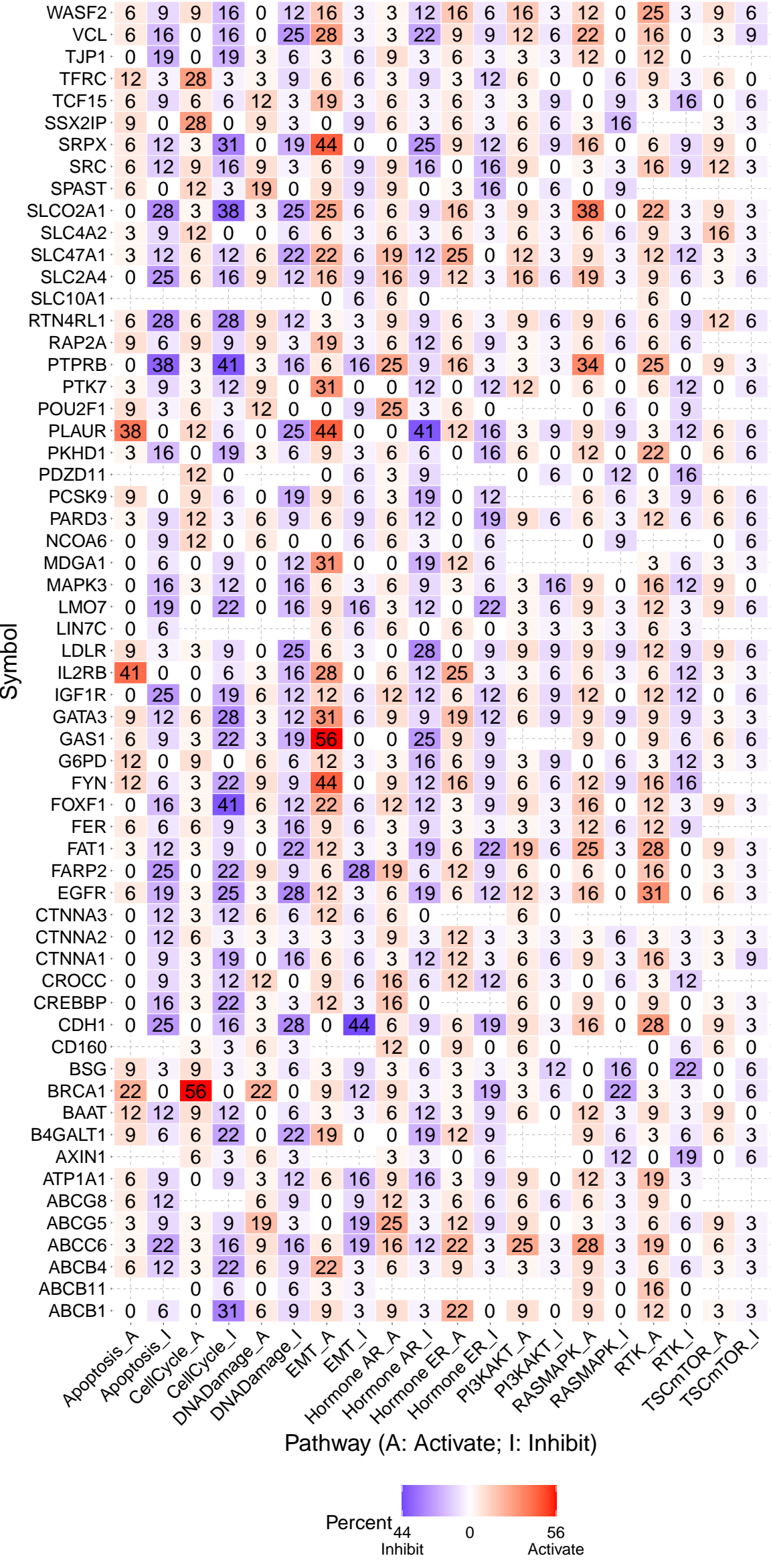

Supplement: Supplementary file 1 [file ijms-23-12784-s001.zip › Supplementary Figure_S1_Pancancer alteration of cancer pathways.pdf]

# SNV percentage heatmap

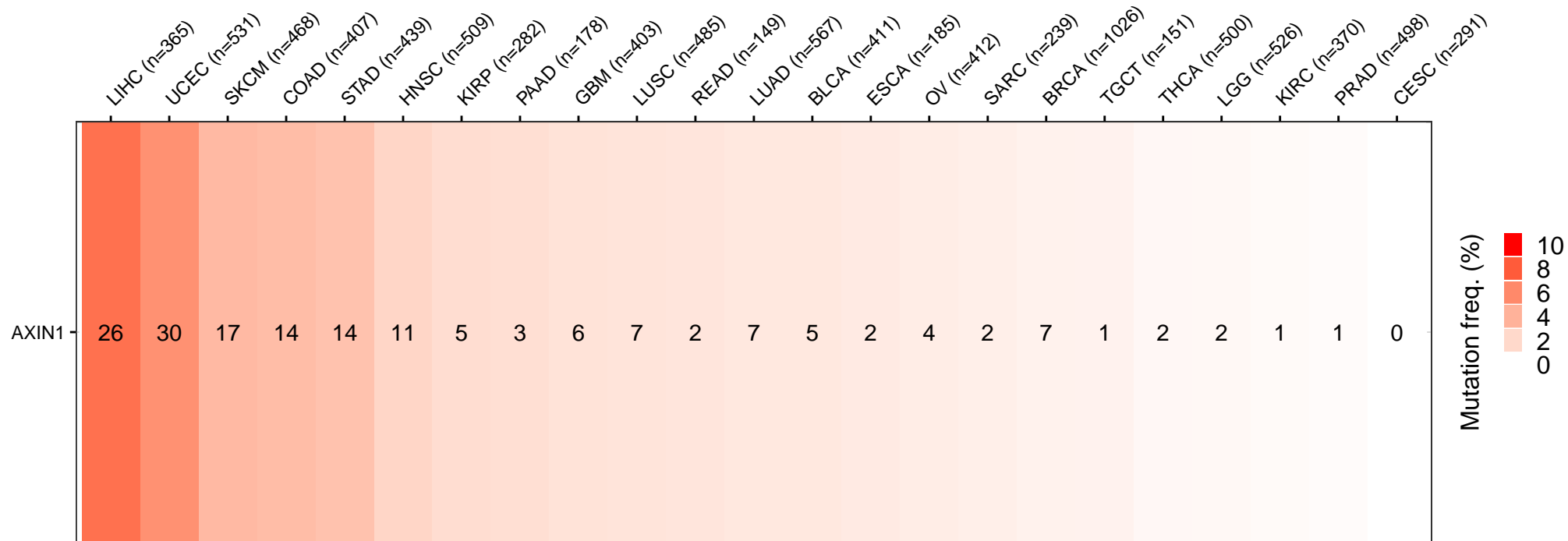

Supplement: Supplementary file 1 [file ijms-23-12784-s001.zip › Supplementary Figure_S3_Pancancer mutation of AXIN1.pdf]

Partial Likelihood Deviance

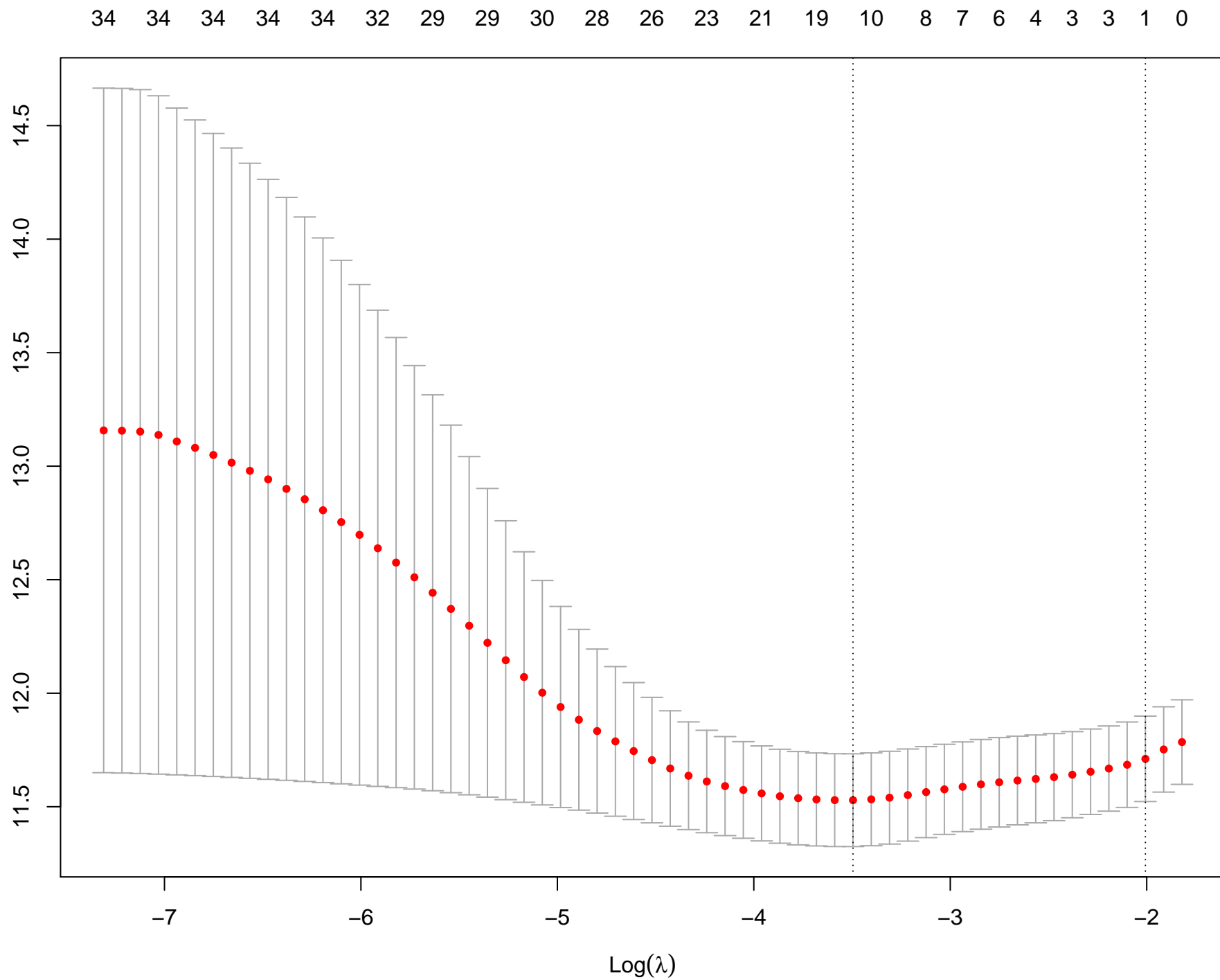

Supplement: Supplementary file 1 [file ijms-23-12784-s001.zip › Supplementary Figure_S5_lamda curve of elastic net.pdf]

GSE76427

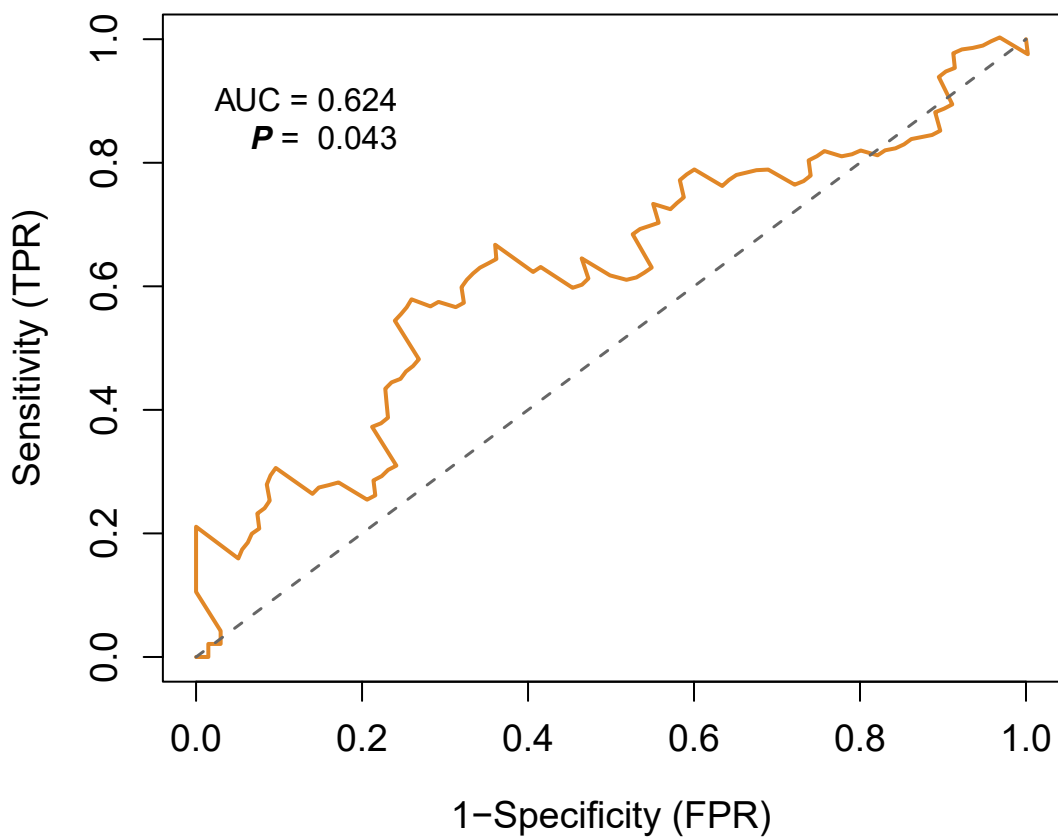

GSE10143

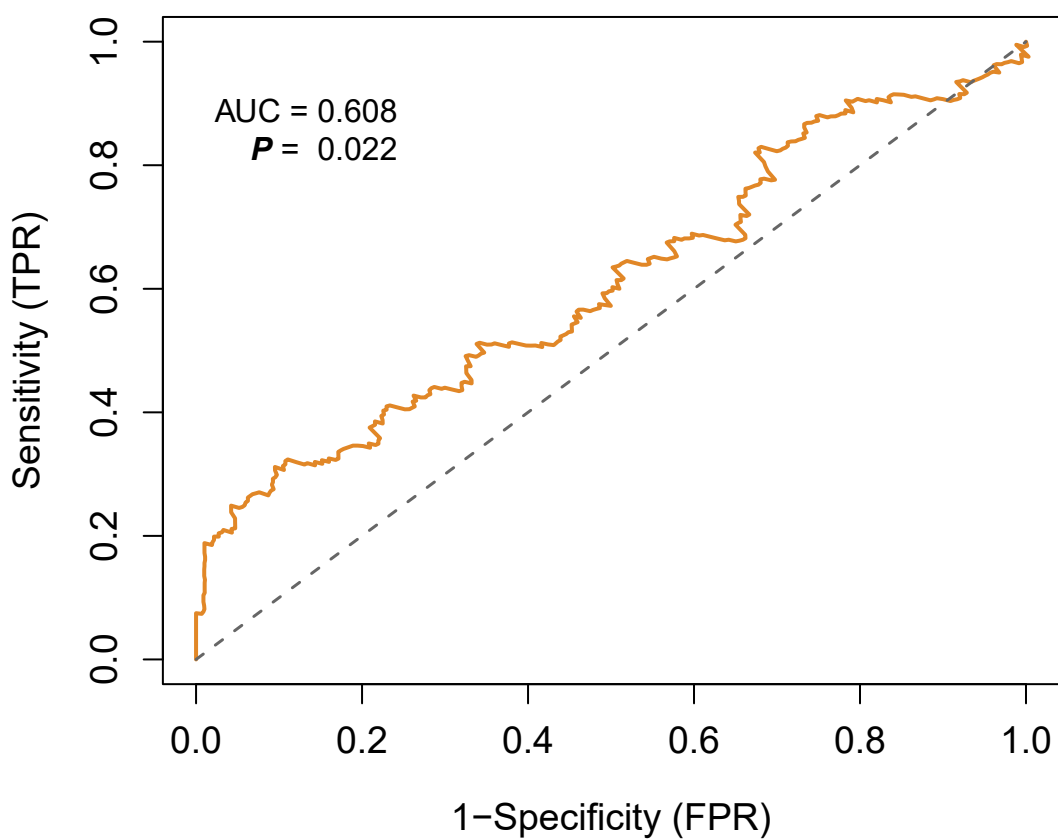

Supplement: Supplementary file 1 [file ijms-23-12784-s001.zip › Supplementary Figure_S6_Model validation of GSE76427 and GSE10143.pdf]

- $\leq 0.05$
- $> 0.05$

- ☐ 0.05
- ☐ 0.01
- ☐ 0.001
- ☐  $\leq 0.0001$

Correlation

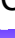

-0.4

0.0

0.5

Supplement: Supplementary file 1 [file ijms-23-12784-s001.zip › Supplementary Figure_S7_Drug sensitivity for CTRP drugs.pdf]
